# Why do hospital prescribers continue antibiotics when it is safe to stop? Results of a choice experiment survey

**DOI:** 10.1186/s12916-020-01660-4

**Published:** 2020-07-30

**Authors:** Laurence S. J. Roope, James Buchanan, Liz Morrell, Koen B. Pouwels, Katy Sivyer, Fiona Mowbray, Lucy Abel, Elizabeth L. A. Cross, Lucy Yardley, Tim Peto, A. Sarah Walker, Martin J. Llewelyn, Sarah Wordsworth

**Affiliations:** 1 Health Economics Research Centre, Nuffield Department of Population Health, University of Oxford, Old Road Campus, Headington, Oxford, OX3 7LF UK; 2 NIHR Oxford Biomedical Research Centre, John Radcliffe Hospital, University of Oxford, Oxford, UK; 3 NIHR Health Protection Research Unit (HPRU) in Healthcare Associated Infections and Antimicrobial Resistance at the University of Oxford in partnership with Public Health England (PHE), Oxford, UK; 4 Centre for Clinical and Community Applications of Health Psychology, University of Southampton, Southampton, UK; 5 Nuffield Department of Primary Care Health Sciences, University of Oxford, Oxford, UK; 6 Department of Microbiology and Infection, Brighton and Sussex University Hospitals NHS Trust, Eastern Road, Brighton, UK; 7 School of Psychological Science, University of Bristol, Clifton, UK; 8 Nuffield Department of Medicine, John Radcliffe Hospital, University of Oxford, Oxford, UK; 9 Oxford University Hospitals NHS Trust, Oxford, UK; 10 Brighton and Sussex Medical School, Brighton, UK

**Keywords:** Antibiotic prescribing, Antibiotic stewardship, Hospitals

## Abstract

**Background:**

Deciding whether to discontinue antibiotics at early review is a cornerstone of hospital antimicrobial stewardship practice worldwide. In England, this approach is described in government guidance (‘Start Smart then Focus’). However, < 10% of hospital antibiotic prescriptions are discontinued at review, despite evidence that 20–30% could be discontinued safely. We aimed to quantify the relative importance of factors influencing prescriber decision-making at review.

**Methods:**

We conducted an online choice experiment, a survey method to elicit preferences. Acute/general hospital prescribers in England were asked if they would continue or discontinue antibiotic treatment in 15 hypothetical scenarios. Scenarios were described according to six attributes, including patients’ presenting symptoms and whether discontinuation would conflict with local prescribing guidelines. Respondents’ choices were analysed using conditional logistic regression.

**Results:**

One hundred respondents completed the survey. Respondents were more likely to continue antibiotics when discontinuation would ‘strongly conflict’ with local guidelines (average marginal effect (AME) on the probability of continuing + 0.194 (*p* < 0.001)), when presenting symptoms more clearly indicated antibiotics (AME of urinary tract infection symptoms + 0.173 (*p* < 0.001) versus unclear symptoms) and when patients had severe frailty/comorbidities (AME = + 0.101 (*p* < 0.001)). Respondents were less likely to continue antibiotics when under no external pressure to continue (AME = − 0.101 (*p* < 0.001)). Decisions were also influenced by the risks to patient health of continuing/discontinuing antibiotic treatment.

**Conclusions:**

Guidelines that conflict with antibiotic discontinuation (e.g. pre-specify fixed durations) may discourage safe discontinuation at review. In contrast, guidelines conditional on patient factors/treatment response could help hospital prescribers discontinue antibiotics if diagnostic information suggesting they are no longer needed is available.

## Background

Antimicrobial resistance (AMR) is a growing global public health problem that threatens to undermine many advances of modern medicine [1]. Reducing unnecessary antibiotic prescribing and thus selective pressure on bacteria to develop resistance is a key strategy to combat AMR [2, 3].

In the English National Health Service (NHS), reductions in antibiotic use have been achieved in primary care by raising the threshold for starting patients on antibiotics [4, 5]. Given the need to initiate antibiotic therapy urgently in life-threatening infection, limiting antibiotic overuse in hospitals depends on prescribers undertaking an early antibiotic prescription ‘review and revise’ at around 48–72 h after a patient starts antibiotic treatment. In England, this approach is supported by ‘Start Smart then Focus’ guidance [6], and in the USA, by ‘Antibiotic Time Outs’ [7]. However, antibiotic prescribing has continued to increase in NHS hospitals, both overall and in terms of certain broad-spectrum, intravenous agents (e.g. quinolones and 3rd, 4th and 5th generation cephalosporins) [5]. Although hospitals only account for around 1/5 of total antibiotic prescribing, this increase is a major concern because this is where most broad-spectrum agents are used. Such agents have the greatest potential to drive resistance and cause adverse drug reactions [8].

The ability to ‘review and revise’ antibiotic prescriptions is a key competency for healthcare professionals who prescribe antibiotics in hospitals [9]. Prescribers may choose to change route or duration of therapy, but only a decision to stop treatment quantitatively reduces antibiotic exposure. In current practice, prescribers frequently do not select this option when it would be safe to do so. For example, while up to 20–30% of initial prescriptions amongst acute medical admissions could be stopped safely, in routine practice, fewer than 10% are typically stopped [10–12]. In a systematic review of in-hospital antibiotic prescribing decisions (including both initial prescribing and de-escalation decisions), sociocultural and behavioural factors, such as fear of adverse health outcomes for patients, intolerance of uncertainty, professional hierarchy and beliefs on the applicability of antibiotic prescribing guidelines, were found to be likely to play a role in these decisions [13]. However, it is likely that the determinants of decisions to start antibiotic treatment and decisions taken at review differ, so that decisions specifically to de-escalate antibiotic treatment are a subset of these factors. Furthermore, there is a lack of quantitative evidence on the relative weights of such determinants, knowledge of which could enable efficient design and implementation of stewardship interventions [13].

This study therefore aimed to quantify the relative importance of key factors influencing prescribers’ decisions on whether to stop or continue antibiotics at ‘review and revise’. To do so, we conducted an online choice experiment, a survey method widely used in health economics [14–16]. The method involves eliciting preferences for alternative healthcare options by asking respondents to make trade-offs between key attributes of these options.

## Methods

### Survey development

The choice survey was developed in accordance with good practice guidelines [17]. The sampling frame was healthcare professionals in the UK, who make antibiotic prescribing decisions in acute/general hospital medicine. There were no other inclusion criteria. We recruited participants through the Society for Acute Medicine (SAM), the national representative body for all clinicians working in acute medical units, and through three of the thirteen postgraduate deaneries, which manage postgraduate medical education in England. We selected one in the South of England (Kent Surrey and Sussex), one in Central England (Thames Valley) and one in the North of England (North East and Cumbria).

The selection of key factors (‘attributes’) likely to influence ‘review and revise’ prescribing decisions was informed by a literature review that we undertook (Additional file 1) and qualitative interviews undertaken as part of the ARK-Hospital research programme [10]. The ARK-Hospital programme is developing a complex behavioural intervention that aims to safely increase antibiotic stopping rates for acute/general medical patients admitted to the hospital. Ultimately, to conduct our choice experiment, we needed to compile a list of between around 4 and 8 attributes that captured the essence of the ‘review and revise’ decision. As in all choice experiments, deciding this final list of attributes, and the precise wording of those attributes, inevitably required an element of judgement [17]. Based on the ARK-Hospital qualitative interviews and the literature review, and drawing on many decades of experience of antibiotic de-escalation decisions within the team, we identified six attributes that we believed captured the central issues driving ‘review and revise’ prescribing decisions (Table 1). We deliberately focused on attributes that were either known at patient presentation in the hospital or were generalizable across patients. This was because individual responses to treatment are very specific (as reflected in a large number of different aspects of response mentioned in respondent comments, Additional file 10), and such heterogeneity could not be adequately captured in the attribute descriptions. Levels were identified for each attribute, informed by the qualitative interviews and clinical expertise within the study team (Table 1).

**Table 1:** Attributes and levels presented to respondents for each choice alternative

Factor	Levels			Coding
	1	2	3	
Patient’s presenting symptoms [SYMPTOMS]	*Symptoms indicating a urinary tract infection, with kidney pain*.	*Fever, cough and possible pulmonary infiltrates on chest X-ray*.	*Off-legs and confused. Possible urinary tract infection, possible lower respiratory tract infection. Might have experienced a fall* [base level so variable not named]^1^.	Categorical variable—effects coded
Whether early discontinuation of antibiotic treatment within 72 h of treatment initiation would be in conflict with local antibiotic guidelines [CONFLICT]	*Early discontinuation would strongly conflict with local antibiotic guidelines.*	*Early discontinuation would somewhat conflict with local antibiotic guidelines*.	*Early discontinuation would not conflict with local antibiotic guidelines* [base level so variable not named]^1^.	Categorical variable—effects coded
Risk of significant harm arising from continued antibiotic treatment [CONTINUE RISK]	*Likely. In 30 cases out of every 100 like this, the patient will experience an adverse effect from continued antibiotic treatment.*	*Somewhat likely. In 10 cases out of every 100 like this, the patient will experience an adverse effect from continued antibiotic treatment.*	*Negligible. In 1 case out of every 100 like this, the patient will experience an adverse effect from continued antibiotic treatment.*	Assumed to be linear—coded as a continuous variable
Risk of significant harm arising from discontinuing antibiotic treatment [STOP RISK]	*Likely. In 30 cases out of every 100 like this, the patient will have a relapse, recurrence or readmission if antibiotic treatment is discontinued.*	*Somewhat likely. In 10 cases out of every 100 like this, the patient will have a relapse, recurrence or readmission if antibiotic treatment is discontinued.*	*Negligible. In 1 case out of every 100 like this, the patient will have a relapse, recurrence or readmission if antibiotic treatment is discontinued.*	Assumed to be linear—coded as a continuous variable
Premorbid condition of the patient [PREMORBID]	*The patient has severe frailty and comorbidities.*	*The patient has moderate frailty and comorbidities.*	*The patient was previously fit and well* [base level so variable not named]^1^.	Categorical variable—effects coded
Level of external pressure to continue antibiotic treatment [EXTERNAL PRESSURE]	*There is no external pressure to continue antibiotic treatment.*	*There is some external pressure to continue antibiotic treatment.*	*There is heavy external pressure to continue antibiotic treatment* [base level so variable not named]^1^.	Categorical variable—effects coded

^1^Ngene codes the top level as the base variable, rather than the bottom variable. Factor names are in square brackets

### Constructing the choice questions

**Fig. 1:**
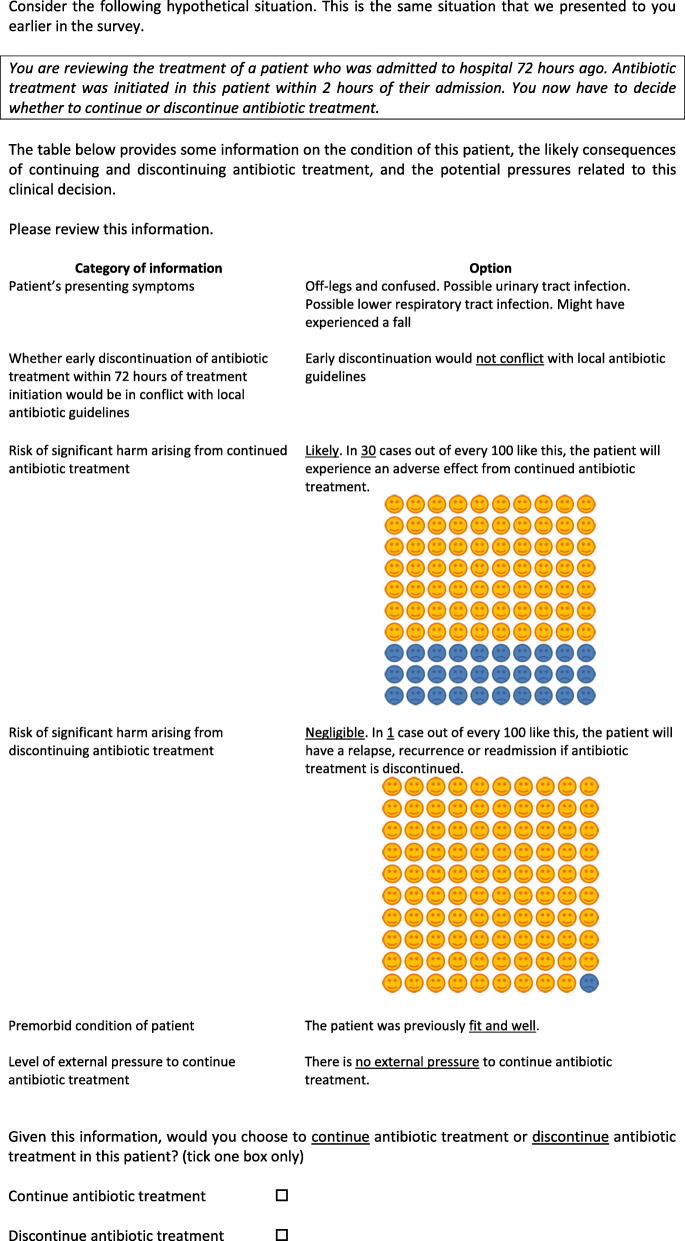
Hypothetical choice situation presented to respondents—practice question

In each choice question, respondents were presented with the same hypothetical situation (Fig. 1), in which they were asked to imagine they were reviewing the treatment of a patient admitted to hospital 72 h ago and in whom antibiotic treatment had been initiated within 2 h of their hospital admission.

Respondents were asked to review a table of information on the status of the patient, the likely consequences of continuing and discontinuing antibiotic treatment, and the potential pressures related to this clinical decision (i.e. the six attributes and their levels). They were asked whether, given this information, they would choose to continue or discontinue antibiotic treatment (to state their preference). Respondents were presented with a total of 15 choice questions (considered an acceptable number) [14, 18, 19], with the levels of attributes varying across each question. The attribute levels presented in each question were generated using experimental design computer software, Ngene (Additional file 2) [20].

### Survey presentation

The choice experiment was conducted as an online survey (Additional file 3). Respondents viewed a participant information page and provided informed consent by answering a series of questions affirmatively (Additional file 3). They were then presented with background information, instructions on how to complete the survey and a description of the attributes and levels (described in the survey as ‘categories of information’). Next, respondents were asked to rank the six attributes in terms of their relative importance when making their decisions and to complete a practice choice question (Fig. 1). This practice question was designed so that the levels presented for each factor should push respondents as far as possible in the direction of choosing to discontinue antibiotics. Respondents then completed the 15 choice questions and undertook a second ranking exercise to investigate whether their preferences had changed during survey completion. Information was also collected on respondent characteristics, including age, gender, primary specialty and clinical grade. Finally, respondents were given an opportunity to offer comments about the survey.

### Piloting and data collection

The survey was piloted amongst twelve healthcare professionals who met the inclusion criteria prior to launch. Respondent choices in the pilot were used to develop a preliminary model of review and revise decisions, in order to fine-tune the experimental design to maximise the information provided whilst minimising participant burden. The pilot data were not included in the final analysis.

Members of SAM and trainees in acute/general medicine registered with the postgraduate deaneries were contacted by email with information about the study, a request to participate and a link to the survey (Additional file 4). The survey was generated and data collected using LimeSurvey, a statistical survey web application. Data collection took place through June 2018–January 2019. We aimed to recruit at least 100 respondents, which is the minimum number indicated by a widely used rule of thumb [21].

### Data analysis

Data analysis used Stata version 15. We estimated a conditional logistic regression model, which includes a fixed effect at the level of the respondent, capturing any unobserved heterogeneity between respondents. However, this model also assumes that attribute coefficients are the same across respondents. As a robustness check, we therefore estimated a mixed effects logistic model, which allows the attribute coefficients to vary across individuals, i.e. to have ‘random effects’.

Both models estimated the impact of the different attributes and levels on the probability of choosing to continue antibiotic treatment. To help with interpretation, the average marginal effect (AME) of each attribute level on the probability of choosing ‘continue’ was calculated. For the categorical attribute levels (levels for presenting symptoms, whether discontinuation would conflict with local prescribing guidelines, premorbid condition of patient, level of external pressure to continue antibiotics), the AME estimates how much higher/lower the probability of continuing was at this attribute level than the probability at the attribute’s base level. For the continuous variables (risk of continuing/risk of discontinuing), the AME estimates how much higher/lower the probability of continuing was for a 1% higher risk.

Respondent characteristics cannot be included in a binary logistic model with respondent-level fixed effects. However, they may be associated with the total number of occasions respondents chose to ‘continue’ versus ‘discontinue’ antibiotics. We investigated this using ordered probit models, where the dependent variable was the number of choice questions in which respondents chose to ‘continue’ antibiotics.

Free-text comments were analysed using inductive content analysis—that is, the categories were suggested by the text rather than an imposed framework [22].

## Results

The survey was completed by 101 people, one of whom was not based in the UK and so was excluded in our analysis. Eighty-two of the 100 included respondents were aged 25–44 years, and acute/general medicine was the primary specialty for 57/99 (58%) (Table 2). Further respondent characteristics are shown in Table 2 and Additional file 5: Personality questions [23]. The respondents listed as ‘non-medical prescriber’ and ‘other’ were mostly specialists in acute/general medicine, and all reported that they made antibiotic review decisions multiple times per month. It therefore seemed reasonable to include them in the sample.

**Table 2:** Descriptive statistics

Variable	*n* (%)
Male	49 (49%)
Age of respondents (*n* = 100)	
Under 25 years	3 (3%)
25 to 34 years	49 (49%)
35 to 44 years	33 (33%)
45 to 54 years	14 (14%)
55 to 64 years	1 (1%)
65 years and over	0 (0%)
Number of beds in hospital (*n* = 98)	
Less than 500	21 (21%)
500–1000	47 (48%)
More than 1000	30 (31%)
Main occupation (*n* = 98)	
Consultant	30 (31%)
Staff grade or associate specialist	4 (4%)
Pre-registration doctors	9 (9%)
Core medical trainee	16 (16%)
Specialty registrars	35 (36%)
Non-medical prescriber (e.g. nurse or pharmacist)	1 (1%)
Other occupations	3 (3%)
Primary clinical specialty (*n* = 98)	
Acute or general medicine	57 (58%)
Microbiology or infectious diseases	14 (14%)
Non-infection related medical specialty	9 (9%)
No primary clinical specialty	3 (3%)
Other	15 (15%)
Risk score out of 10, mean (SD) (*n* = 97)^1^	6.1 (1.8)

^1^*SD* standard deviation. Respondents were asked to rate their attitude to risk on a 0 to 10 scale, where 0 means ‘risk averse’ and 10 means ‘fully prepared to take risks’

There were minor differences in how respondents ranked the attributes before and after completing the choice questions (Additional file 6). Beforehand, the most important attribute was ‘patient’s presenting symptoms’, followed by ‘risk of significant harm arising from discontinuing antibiotic treatment’. The least important attribute was ‘level of external pressure to continue antibiotic treatment’. The attribute rankings remained the same after completing the survey, except that the order of the first and second most important reversed.

### Choice experiment results

**Fig. 2:**
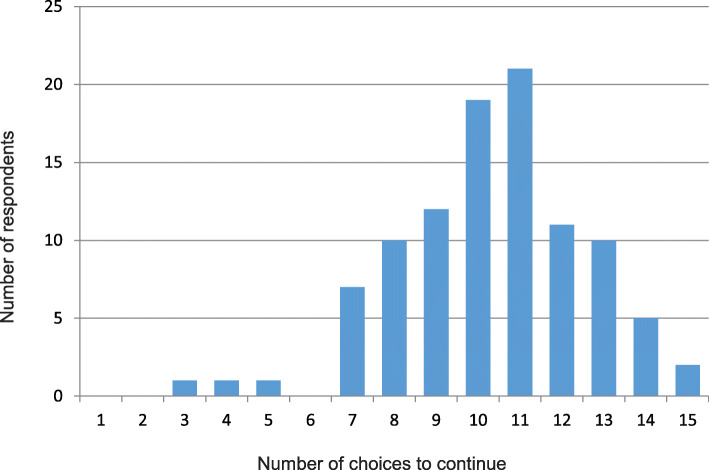
Histogram of number of questions in which respondents chose to continue antibiotics

All respondents completed all 15 choice questions, yielding 1500 choices in total. ‘Continue’ was selected in 1032 (69%) choices. The minimum number of times any respondent chose to ‘continue’ antibiotics across the 15 choice questions was 3, and the maximum was 15 (2 respondents) (Fig. 2). The number of choices to continue/discontinue varied by question from 99/1 to 29/71, with some close to 50/50 (Additional file 7: Table S3).

The conditional logistic model for choosing to continue versus discontinue antibiotics with respondent-level fixed effects (Table 3) had a McFadden pseudo-*R*-squared value of 0.320, indicating an ‘excellent fit’ ([24], p. 35), with all coefficients having the expected sign indicating face validity. For each attribute, at least one level was highly significant, with sizable AMEs. The largest AME came from conflict with local guidelines, with respondents more likely to continue antibiotics when early discontinuation would ‘strongly conflict’ with local guidelines (AME on the probability of continuing + 0.194 (*p* < 0.001) versus ‘no conflict’). However, they were no more/less likely to continue antibiotics when there was only ‘some conflict’. The next highest AME was for the ‘presenting symptoms’ level most strongly supporting the need for antibiotics (AME of typical symptoms of urinary tract infection + 0.173 (*p* < 0.001) versus unclear symptoms). Respondents were also more likely to continue antibiotics when patients had severe frailty and comorbidities (AME = + 0.101 (*p* < 0.001) versus patients previously fit and well). Conversely, they were less likely to continue antibiotics when there was no external pressure to do so (AME = − 0.101 (*p* < 0.001) versus heavy pressure). Decisions were also influenced by the competing risks of continuing treatment (e.g. antibiotic resistance) (AME of 1% point higher risk − 0.013 (*p* < 0.001)) versus discontinuing (e.g. treatment failure) (AME of 1% point higher risk + 0.026 (*p* < 0.001)). Sensitivity analyses using mixed effects logistic models were broadly similar, with comparable AMEs but poorer model fit (Additional file 8).

**Table 3:** Conditional logistic regression analysis

Attribute^2^	Coefficient	SE	Lower CI	Upper CI	Average marginal effect (AME)^1^	*p*
SYMPTOMS						
UTI symptoms with kidney pain	1.132	0.155	0.829	1.436	0.173	< 0.001
Fever, cough and possible pulmonary infiltrates on chest X-ray	0.246	0.108	0.034	0.459	0.038	0.023
Unclear symptoms^3^	− 1.379	–	–	–	–	–
CONFLICT WITH GUIDELINES						
Strongly conflict	1.275	0.131	1.018	1.532	0.194	< 0.001
Somewhat conflict	0.073	0.089	− 0.101	0.248	0.011	0.411
No conflict^3^	− 1.348	–	–	–	–	–
CONTINUE RISK	− 0.085	0.012	− 0.108	− 0.061	− 0.013	< 0.001
STOP RISK	0.172	0.017	0.139	0.205	0.026	< 0.001
PREMORBID CONDITION						
Severe frailty and comorbidities	0.660	0.138	0.390	0.930	0.101	< 0.001
Moderate frailty and comorbidities	0.300	0.098	0.108	0.492	0.046	0.002
Fit and well^3^	− 0.960	–	–	–	–	–
EXTERNAL PRESSURE						
No pressure	− 0.660	0.097	− 0.851	− 0.469	− 0.101	< 0.001
Some pressure	− 0.104	0.094	− 0.289	0.080	− 0.016	0.268
Heavy pressure^3^	0.764	–	–	–		–
McFadden’s *R*^2^ (pseudo-*R*^2^)	0.320					
AIC/BIC	976.767/1029.697					
Log-likelihood	− 478.384					
*N*	1470					

^1^AME is the average marginal effect of each factor level on the probability of choosing to continue. For the categorical factor levels, this indicates how much higher/lower the probability of continuing was at this attribute level than the probability at the factor’s base level. For the continuous variables (risk of continuing/risk of discontinuing), the AME indicates how much higher/lower the probability of continuing was for a 1% higher risk. *AIC* Akaike Information Criterion, *BIC* Bayesian Information Criterion, *CI* 95% confidence interval. *p p* value of coefficient, *SE* standard error clustered at the respondent level

^2^Attribute descriptions: SYMPTOMS = patient’s presenting symptoms (1 = UTI and kidney, 2 = fever cough and funny X-ray, 3 = unclear [base level]); CONFLICT WITH GUIDELINES = whether early discontinuation of antibiotic treatment within 72 h of treatment initiation would be in conflict with local antibiotic guidelines (1 = strongly conflict, 2 = somewhat conflict, 3 = not conflict [base level]); CONTINUE RISK = risk of significant harm arising from continued antibiotic treatment, expressed as a percentage; STOP RISK = risk of significant harm arising from discontinuing antibiotic treatment, expressed as a percentage; PREMORBID CONDITION = premorbid condition of the patient (1 = severe frailty and comorbidities, 2 = moderate frailty and comorbidities, 3 = fit and well [base level]); and EXTERNAL PRESSURE = level of external pressure to continue antibiotic treatment (1 = no pressure, 2 = some pressure, 3 = heavy pressure [base level]). The sample size arises from 98 respondents times 15 choice questions. The 2 respondents who chose ‘continue’ in all 15 choice questions were omitted because of the lack of variation in their responses

^3^These attributes were effects-coded. The coefficients of the base levels (unclear symptoms, no conflict, fit and well and heavy pressure) were calculated as the negative sum of the coefficients of the other levels

Being a consultant was associated with being less likely to continue prescribing antibiotics, i.e. in terms of the total number of times respondents chose to continue antibiotics across the 15 choice questions (Additional file 9). However, we did not find evidence for modification of the effects of attributes in the conditional logistic model in Table 3 by being a consultant versus other grades (interaction *p* > 0.06 for all attribute levels). Prescriber personality traits (Additional file 5) were also associated with prescribing choices across the 15 questions. Extraversion was associated with being more likely to choose to continue antibiotics; agreeableness was associated with being less likely to continue antibiotics (Additional file 9).

### Comments from respondents

Most respondents found the survey neither easy nor difficult to complete: the median (IQR) difficulty score was 4 (3–5) on a 1–7 scale (where 1 = very easy and 7 = very difficult).

One third of the respondents left feedback on the survey (Additional file 10), falling into several categories (Table 4). The most common category stressed the importance to review and revise decisions of additional clinical information not captured in our experiment, particularly regarding response to treatment, and whether the patient was improving or deteriorating at the ‘review and revise’ stage.

**Table 4:** Categories of respondent comments

Category	Description of category content
Importance of clinical information, especially response to treatment	This category highlighted the importance to review and revise decisions of signs that patients are improving or deteriorating following treatment. Such comments referred, for example, to the importance of the information provided by clinical assessment and observations, such as temperature, culture results and inflammatory markers.
Somebody else’s problem	This category expressed the view that antibiotic use in secondary care contributes relatively little to antibiotic resistance and that the focus of antibiotic stewardship should be elsewhere, such as primary care, agriculture or in other countries.
Critique of the study	Comments in this category criticised elements of the choice questions. Examples included an assertion that chest X-rays produce clear results and that describing the results in our factor-level description as indicating ‘possible infiltrates’ was ‘daft’.
Didactic guidelines	This category contained comments on the guidelines being too risk averse and reluctance not to follow the guidelines ‘when the stakes are high’ unless advised by a senior colleague.
The role of external pressure depends on the context and on where the pressure is coming from	A comment suggested that whether external pressure to continue antibiotics had an effect on review and revise decisions depends on where the pressure is coming from—for example, pressure from a consultant would have much more impact than pressure from patients’ relatives.
In real-life levels of harm from continuing/discontinuing antibiotics are harder to quantify	This category expressed the view that in real clinical practice, the actual risk levels of continuing/discontinuing antibiotics are not clear-cut as presented in our choice experiment. Instead, they are ambiguous and must be inferred, for example, the degree of confidence in the diagnosis.

## Discussion

Using a choice experiment, we have quantified the relative importance of attributes likely to play a key role in decisions to (dis) continue antibiotics at the time of ‘review and revise’ amongst acute/general medical patients. The choice of attributes included was informed by literature review and qualitative interviews, combined with experience within the team. The attribute level with the largest marginal effect on prescribing was ‘early discontinuation of antibiotics being in ‘strong conflict’ with local guidelines’. However, early discontinuation ‘somewhat conflicting’ with local guidelines was not significant. This suggests interventions to make guidelines less prescriptive about antibiotic continuation could help increase appropriate early discontinuation.

In NHS hospitals, antibiotic guidelines do not generally acknowledge the uncertainty that exists prior to the ‘review and revise’ decision, but make didactic statements stipulating durations, such as ‘duration: 7 days’, which are not likely to be appropriate for many patients [25]. Furthermore, quality improvement assessments of antibiotic prescribing routinely audit how often prescriptions match guidelines. Prescribers are therefore confronted with a situation in which a patient has been started on antibiotics, with an indication recorded for which guidelines specify a duration, and hence, stopping antibiotics early will lead to a poor audit outcome. Guidelines might better support bedside stewardship decisions if they made duration recommendations only for patients in whom ‘review and revise’ has determined that antibiotics are truly indicated. They could also recognise that clinical response should be considered, for example, replacing ‘duration: 7 days’ with ‘duration: up to 7 days depending on patient factors and treatment response’. This could provide guidance to (often relatively junior) clinicians on duration decisions, minimising potential for poor patient outcomes, without unnecessarily overriding their clinical judgement. The content analysis highlighted the importance of treatment response, whether patients are improving or deteriorating. This suggests respondents are already using clinical judgement when making decisions; guidelines which recognise this might empower them to discontinue antibiotics more often.

Consultant respondents were significantly more likely to discontinue antibiotics, consistent with previous studies suggesting junior doctors focus more on conservative prescribing practice to avoid censure by senior colleagues [26]. However, there was no significant interaction between consultant status and any of the attribute levels in the prescribing decision. This suggests that consultants are more prone to discontinue antibiotics in general, rather than any of these six attributes influencing their decisions differently to other grades.

The competing risks of continuing versus discontinuing antibiotics both played an important role in review decisions. However, a higher risk of discontinuing affected decisions about twice as much as the same higher risk from continuing. This supports the view that the risk from continuing antibiotics is much less salient in clinical practice than the risk from discontinuing them [27]. One interpretation might be that it may be valuable to make risks of continuing unnecessary antibiotics, e.g. from *Clostridioides difficile* diarrhoea or adverse drug reactions [8], more salient to clinicians. However, as several respondents noted, in the choice experiment, the risks from continuing and discontinuing antibiotics were quantified and made explicit, whereas in clinical practice, these risks are harder to quantify. Without this information, factors such as presenting symptoms and response to treatment may be used to infer these risks.

We found respondents were much less likely to continue antibiotics when there was no external pressure to do so—10% less likely than when faced with ‘heavy’ external pressure. This contrasts with responses to the attribute ranking exercise, where external pressure was ranked as the least important attribute. The ability of choice experiments to better capture underlying preferences—and thus actual decision-making—versus direct questions about preferences is considered one of their key strengths [28]. It is possible that respondents were either unaware of the extent to which pressure is an influence or, due to social desirability bias, did not want to admit it in the ranking exercise. This raises questions as to whether external pressure to continue antibiotics can be reduced, or whether the way prescribers respond to external pressure can be changed, e.g. through training. One limitation however is that different respondents may have interpreted pressure in different ways. One respondent commented that the relevance of this attribute depends on where the pressure is coming from; it would be extremely important if it was coming from within the team, especially a consultant, but much less so if from patients’ relatives.

Previous studies have identified sociocultural and behavioural factors likely to play a role in hospital antibiotic prescribing decisions, including attitudes to guidelines and tolerance of risk of undertreating infections [13]. Quantitative evidence on the relative importance of different factors can support the design and implementation of more efficient stewardship interventions, by directing interventions to target the most influential factors [13]. To our knowledge, this is the first study to quantify the relative importance of such factors.

Our study also has limitations. First, though the research question is of wider relevance, the setting for the study was the NHS in England. In other settings, the factors driving early discontinuation of antibiotics may vary. For example, in the USA and elsewhere, procalcitonin measurements are used widely to guide discontinuation decisions in some clinical scenarios [29, 30]. This is not true currently of UK practice outside critical care units. Second, reported actions in a survey may not reflect actual choices in clinical practice, though asking respondents to make trade-offs rather than state absolute preferences may partially mitigate this limitation. Third, our sample size may be considered small, but choice experiments such as this one typically require smaller sample sizes than many other types of regression-based analyses. In essence, this is because each respondent completes a number of choice tasks (15 in this study), which multiplies the amount of information captured from each respondent. Thus, sample sizes of 100 or lower are not unusual [31]. Fourth, though informed by qualitative interviews and literature review, deciding which factors to include in the choice experiment inevitably required an element of judgement, and the factors we used to characterise the ‘review and revise’ decision are necessarily a simplification. Several respondents said that in clinical practice, they would base their decisions on treatment response evidence, e.g. from clinical assessment, observations, cultures and inflammatory markers. However, one important aspect of clinical management is that antibiotics are generally initiated by the admitting clinical team, with a different clinical team responsible for ‘review and revise’ 48–72 h later. The lack of clear understanding about the reason that antibiotics were initially started is one specific aspect that the ARK-Hospital programme is designed to address [32]. Some respondents also said that, in real life, levels of harm from continuing/discontinuing antibiotics are harder to quantify and felt that the availability of the explicit percentage risks was an oversimplification. Only a third of respondents provided qualitative data however, and they may not have been typical of the whole sample. Though it is noteworthy that consultants chose to continue prescribing less often than non-consultants, an analysis of which professional factors are related to continuing antibiotics when they should actually be discontinued was beyond the scope of this study. This would potentially be a valuable avenue for future research.

## Conclusions

In this study we have quantified the relative importance of factors likely to play a key role in ‘review and revise’ decisions on (dis) continuing antibiotics in hospitals. Our results are suggestive of several potential barriers to safe early discontinuation of antibiotics, with early discontinuation ‘strongly conflicting’ with local guidelines being key. Revising guidelines to be less prescriptive about duration, and instead making duration conditional on patient factors and treatment response, could help to safely increase the frequency of early discontinuation of antibiotics. Addressing the other barriers to early discontinuation suggested by our results may be more challenging. More research is urgently needed to identify and quantify barriers and facilitators of safe early discontinuation of antibiotics in other healthcare settings.

## Supplementary information

**Additional file 1:** Literature review.

**Additional file 2:** Experimental design.

**Additional file 3:** Full text of final choice experiment survey.

**Additional file 4:** Recruitment email text.

**Additional file 5:** Personality questions. **Table S1.** Mean scores for the five key personality traits.

**Additional file 6:** Attribute rankings and responses to the practice question. **Table S2.** Attribute rankings.

**Additional file 7:** Continue/Discontinue split across the 15 choice questions. **Table S3.** Continue/Discontinue split.

**Additional file 8:** Robustness Analysis: Mixed effects logistic regression analysis. **Table S4.** Mixed effects logistic regression analysis.

**Additional file 9:** Association of choices with respondent characteristics. **Table S5.** Ordered Probit Models of Continuing Antibiotics.

**Additional file 10:** Respondent comments on survey.

## Data Availability

The datasets generated and analysed during the current study are available from the corresponding author on reasonable request.

## Abbreviations

AME: Average marginal effect
AMR: Antimicrobial resistance
IQR: Interquartile range
NHS: National Health Service
SAM: Society for Acute Medicine
