# Supplementary material for: Why do hospital prescribers continue antibiotics when it is safe to stop? Results of a choice experiment survey
Source: BMC Med. 2020 Jul 30;18:196. doi: 10.1186/s12916-020-01660-4 (PMC7391515; doi:10.1186/s12916-020-01660-4)
Supplement: Supplementary file 1 — Additional file 1: Literature review. [file 12916_2020_1660_MOESM1_ESM.docx]

**Additional file 1: Literature review**

**Methods**

We performed a structured literature review to ensure that we did not overlook any attribute that might conceivably be important in antibiotic (dis)continuation decisions. Though the determinants of antibiotic (dis)continuation decisions are likely to differ in important respects from antibiotic initiation decisions, we believed it to be likely that there may be some overlap. We therefore adopted a very conservative approach whereby we sought to include all studies that could provide information about preferences for antibiotic prescribing decisions, broadly conceived.

A secondary pragmatic reason for taking a broad approach was that the literature review was also intended to support further choice experiments on antibiotic prescribing in different contexts. The approach therefore was to generate a long-list of attributes that could potentially influence clinicians, patients, or members of the public, in giving, seeking or stopping antibiotic treatment for any condition, , with of a view to ensuring that we would not miss anything relevant to ‘review and revise’ decisions.”

The search strategy combined terms related to antibiotics, or antimicrobial resistance, with terms related to preferences or DCEs. The search syntax was:

(antibacterial OR anti-bacterial OR antibiotic* OR anti-infective OR antimicrobial* OR anti-microbial* OR AMR) AND (preference* OR DCE OR conjoint* OR best-worst* OR BWS OR discrete choice*)

Searches were restricted to studies in humans. The search syntax was applied in four databases between the following dates:

|  | PubMed | Embase | Econlit | PsychInfo |
| --- | --- | --- | --- | --- |
| Date range searched | 01/01/2005-12/02/2017 | 01/01/2005-12/02/2017 | 01/01/2005-12/02/2017 | 01/01/2005-13/07/2017 |

The literature search was undertaken in July 2017 by LSJR and LA. Following deduplication, publications were scanned for relevance by title and abstract, and clearly irrelevant publications were excluded. A number of papers not identified by the search but already known by the study team and considered potentially relevant were also included. The full texts of the remaining publications were then evaluated for relevance. This involved carefully reading the papers and tabulating in a Word document a brief description of any factors that the papers suggested may be related to antibiotic prescribing preferences, broadly conceived, and might therefore be relevant to any of our planned choice experiments, including this one. Factors that were identical, or almost identical, were grouped together. This resulted in a table with a ‘long-list’ of potential attributes. This completed long-list was then reviewed by LSJR, LA and JB to check that the groupings of potential attributes were consistent, e.g. that any identical potential attributes were grouped together.

As part of the literature review, we also produced a summary document briefly describing the objectives, setting (e.g. primary care versus secondary care) and preference perspective (e.g. patients, clinicians or patients) of all the papers included in the review.

**Results**

The search identified 3,066 papers. After removing duplicates, screening titles and abstracts, and then assessing full-text papers for eligibility, 89 papers were identified that met the inclusion criteria. In addition, 23 papers were identified from other sources (for example, papers that were already known to the study team). Overall, 112 papers were included in the review.

116 potential attributes were identified in these papers. Of these, 46 appeared in only one paper, whereas 20 appeared in 10 or more papers.

**‘Long List’ of potential attributes identified**

|  | Attribute | Count | Source |
| --- | --- | --- | --- |
| 1. | Recommended in guidelines | 18 | 1, 2, 7,20,24,31,32,37,40,43,50,51,72,82,91,99,102,107 |
| 2. | Originally prescribed by senior colleague | 2 | 1,17 |
| 3. | Drug choice of hospital committee / protocol / computer decision support system | 9 | 1,10,17,20,32,55,70,71,79 |
| 4. | Recommended by peer | 10 | 1,51,55,71,77,91,93,94,95,97 |
| 5. | Indication for drug is documented on drug chart | 1 | 1 |
| 6. | Duration of drug is documented on drug chart | 1 | 1 |
| 7. | Recommended/preferred by senior colleague or specialist | 6 | 1,7,20,50,55,82 |
| 8. | Risk of infection (inc previous infection as indicator) | 13 | 2,8,9,10,11,12,13,18,20,23,24,27,74 |
| 9. | Likelihood that bacterial infection is present | 2 | 58,65 |
| 10. | Risk of future bacterial infection (e.g. with/without prophylaxis) (inc previous infection as indicator of increased susceptability) | 10 | 39,40,42,43,47,56,57,83,88,89 |
| 11. | Indicated in literature | 10 | 2,32,38,43,51,55,59,71,77,91 |
| 12. | Personal preference | 1 | 2 |
| 13. | Influence from education | 1 | 2 |
| 14. | Fear of litigation | 3 | 2,26,76 |
| 15. | Risk of complications or adverse events | 10 | 3,8,12,13,14,18,20,22,23,24 |
| 16. | Risk/fear of complications | 14 | 51,66,85,91,92,99,100,101,102,103,104,105,106,107 |
| 17. | Risk of adverse events (e.g. toxicity) | 12 | 38, 39,40,47,57,60,66,70,83,85,87,90 |
| 18. | Risk of death | 4 | 3,39,40,45 |
| 19. | Life expectancy with treatment | 3 | 38,49,90 |
| 20. | Risk of sickness/symptoms returning | 2 | 3,24 |
| 21. | Likelihood of needing anaesthesia | 3 | 3,24,83 |
| 22. | Likelihood of needing surgery | 1 | 3 |
| 23. | Having a scar(s) | 1 | 3 |
| 24. | Having to stay in the hospital (e.g. for IV antibiotics) | 2 | 3,48 |
| 25. | Non-clinical patient factors, e.g. missing days of work/school, socio-economic background | 7 | 3,91,92,93,99,105,108 |
| 26. | Missing days taking care of family/children | 1 | 3 |
| 27. | Cost (including financial incentives) | 29 | 3,16,24,38,39,40,46,47,51,57,59,60,66,67,70,76,78,80,81,84,91,92,93,99,102,105,106,110,111 |
| 28. | Not being able to eat a normal diet | 1 | 3 |
| 29. | Not being able to do normal/usual activities | 1 | 3 |
| 30. | Discharge instructions easy to understand | 1 | 4 |
| 31. | Severity of pain, infection or other signs/symptoms | 25 | 3,5,10,12,13,19,20,24,31,32,33,49,51,52,53,57,64,70,98,99,100,102,105,107,111 |
| 32. | Complexity of surgical procedure being performed | 1 | 57 |
| 33. | Patient has comorbidities | 13 | 5,20,27,35,52,53,57,58,85,89,99,102,112 |
| 34. | Patient’s age | 16 | 5,9,19,24,26,32,34,35,39,52,57,58,86,99,102,108 |
| 35. | Patient gender | 2 | 34,58 |
| 36. | Patient social status/category | 2 | 34,58 |
| 37. | Patient has antibiotic allergy or contraindications | 12 | 5,11,14,20,57,60,62,72,78,99,102,112 |
| 38. | Drug has few contraindications | 1 | 70 |
| 39. | Breadth of spectrum of action | 1 | 70 |
| 40. | Probability of crossed resistance with other antibiotics | 1 | 70 |
| 41. | Activity of antibiotic against specific bacteria | 1 | 70 |
| 42. | Antibiotic half-life | 1 | 70 |
| 43. | Frequency of reactions at place of drug administration | 1 | 70 |
| 44. | Volume of saline solution needed to dilute antibiotic for IV administration | 1 | 70 |
| 45. | Mode or route of treatment (e.g. oral; IV) | 14 | 5,11,14,15,16,24,28,38,39,40,48,62,76,81 |
| 46. | Frequency of treatment | 4 | 39,40,61,73 |
| 47. | Duration of treatment | 5 | 61,67,70,73,81 |
| 48. | Length of hospital stay or follow-up treatment needed | 3 | 5,12,60 |
| 49. | Drug safety (real or perceived) | 6 | 5,12,36,59,80,90 |
| 50. | Personal experience of drug (clinician) | 2 | 5,70 |
| 51. | Personal experience of drug (patient) | 1 | 76 |
| 52. | Anticipated patient adherence | 5 | 5,24,39,60,88 |
| 53. | Patient or family preferences / expectations / requests | 27 | 6,12,13,19,21,22,24,26,28,30,33,37,44,46,50,51,53,54,60,61,65,76, 81,91,92,96,98 |
| 54. | Risk of AMR developing (patient or pop level) | 10 | 8, 12,13,40,47,57,66,83,87,88 |
| 55. | Risk of AMR developing – population level | 4 | 39,60,99,101 |
| 56. | Risk of patient developing a resistant infection | 1 | 39 |
| 57. | Indicated (or not indicated) by cultures or other diagnostic test | 18 | 10,12,19,29,32,35,37,44,51,53,54,60,65,70,74,78,82,91 |
| 58. | Interacting medications | 1 | 11 |
| 59. | Organ function (e.g. Renal insufficiency) | 4 | 11,32,72,75 |
| 60. | Resistant / MDR infection | 4 | 11,20,25,62 |
| 61. | Likely treatment efficacy (real or perceived) | 13 | 12,13,22,24,46,66,80,83,87,88,90,91,93 |
| 62. | Fever | 6 | 13,19,24,76,99,105 |
| 63. | GP/health professional advises treatment | 6 | 13,22,28,76,84,90 |
| 64. | Treatment advised on website | 1 | 13 |
| 65. | Treatment advised by mum or other family member | 1 | 13 |
| 66. | Have received ABs before for similar symptoms | 2 | 13,30 |
| 67. | Treatment time consuming (e.g. stay in hospital or pill burden) | 3 | 16,27,83 |
| 68. | Likely presence/severity of drug side effects | 10 | 16,22,23,38,39,40,46,61,73,84 |
| 69. | Patient’s ethnicity | 1 | 21 |
| 70. | Treatment uncomfortable, painful or distressing | 3 | 23,28,83 |
| 71. | Response to current drug | 1 | 24 |
| 72. | Inflammatory markers | 2 | 24,36 |
| 73. | GP has good relationship/rapport with patient | 1 | 30 |
| 74. | Type of infection | 1 | 32 |
| 75. | Length of time patient has been in hospital | 1 | 32 |
| 76. | Risk of infectious disease spreading | 1 | 32 |
| 77. | Patient’s history /medical records | 3 | 32,82,85 |
| 78. | Information visualisation tool | 1 | 32 |
| 79. | Ease of administration | 3 | 36,46,84 |
| 80. | Clinical response to antibiotics | 2 | 36,84 |
| 81. | Patient convenience (e.g. if difficult for patient to return to the surgery, or many visits needed) | 6 | 37,38,45,60,80,84 |
| 82. | Patient’s or (family’s) attitude to risk | 1 | 38 |
| 83. | Patient’s or (family’s) time preference (e.g. willingness to trade current health for future health) | 1 | 38 |
| 84. | Patient’s (or family’s) knowledge of condition and/or management options | 5 | 38,48,51,60,68 |
| 85. | Patient’s (or family’s) preferences for treatment decision | 1 | 38 |
| 86. | Patient’s specific clinical circumstances | 4 | 38,89,99,112 |
| 87. | Anticipated quality of life | 2 | 38,40 |
| 88. | Likelihood of readmission | 2 | 38,45 |
| 89. | Likelihood of ICU admission | 2 | 38,45 |
| 90. | Fear of shortcomings in negative microbiological results | 1 | 41 |
| 91. | Drug quality | 1 | 50 |
| 92. | Ease of explaining prescription decision in consultation time (Relates to time pressure; e.g. takes longer to explain antibiotic not needed) | 8 | 51,54,91,92,93,99,106,109 |
| 93. | Discoloured sputum/phlegm | 5 | 53,64,99,100,107 |
| 94. | Abnormal lung sounds | 3 | 33,53,63 |
| 95. | Influenced by pharmaceutical representative/company | 4 | 55,58,99,106 |
| 96 | Time of year | 1 | 58 |
| 97. | Lack of clarity over legality of prescribing | 1 | 60 |
| 98. | Probability of treatment succeeding | 1 | 61 |
| 99. | Patient is a smoker | 1 | 64 |
| 100. | Patient is taking an anti-inflammatory drug | 1 | 64 |
| 101. | How well treatment alleviates symptoms | 1 | 69 |
| 102. | Patient’s immune system is compromised | 2 | 75,85 |
| 103. | Whether patient has health insurance/sufficient funds | 4 | 76,77,99,111 |
| 104. | Colour of nasal discharge | 1 | 81 |
| 105. | Dose | 1 | 81 |
| 106. | Medication availability | 1 | 82 |
| 107. | Information on resistance patterns/rates (e.g. from antibiogram) | 2 | 25,91 |
| 108. | Treatment feels like action is being taken or offering something tangible | 2 | 83,91 |
| 109. | Open versus closed fracture | 1 | 87 |
| 110. | Uncertainty over diagnosis | 12 | 33,91,92,99,102,103,104,105,106,107,110,111 |
| 111. | Local antibiotic prescribing rates for similar condition | 1 | 91 |
| 112. | GP’s uncertainty about access to patient for review | 2 | 91,92 |
| 113. | Whether prescribing decision will affect GP workload | 2 | 51,91 |
| 114. | GP’s fear of losing patient as a result of treatment decision (e.g. not prescribing antibiotics) | 3 | 99,105,106 |
| 115. | Treatment decision is seen as a ‘quick fix’ (by either GP or patient) | 7 | 99,102,104,105,106,108,109 |
| 116. | Patient is anxious | 2 | 99,108 |

References in Literature Review

1. Panesar P, Jones A, Aldous A, Kranzer K, Halpin E, Fifer H, et al. (2016) Attitudes and Behaviours to Antimicrobial Prescribing following Introduction of a Smartphone App. PLoS ONE 11(4): e0154202. <https://doi.org/10.1371/journal.pone.0154202>

2. Marecek GS, Earhart JS, Gardner MJ, Davis J, Merk BR. Surgeon preferences regarding antibiotic prophylaxis for ballistic fractures. Archives of orthopaedic and trauma surgery. 2016 Jun 1;136(6):751-4.

3. Kadera SP, Mower WR, Krishnadasan A, Talan DA. Patient perspectives on antibiotics for appendicitis at one hospital. Journal of Surgical Research. 2016 Apr 30;201(2):253-7.

4. Olives TD, Patel RG, Thompson HM, Joing S, Miner JR. Seventy-two-hour antibiotic retrieval from the ED: a randomized controlled trial of discharge instructional modality. The American journal of emergency medicine. 2016 Jun 30;34(6):999-1005.

5. Lane S, Johnston K, Sulham KA, Syed I, Pollack CV, Holland T, Nathwani D. Identification of patient characteristics influencing setting of care decisions for patients with acute bacterial skin and skin structure infections: results of a discrete choice experiment. Clinical therapeutics. 2016 Mar 31;38(3):531-44.

6. Ang GC, Zhang D, Lim KH. Differences in attitudes to end-of-life care among patients, relatives and healthcare professionals. Singapore medical journal. 2016 Jan;57(1):22.

7. Chow AL, Ang A, Chow CZ, Ng TM, Teng CD, Ling LM, Ang BS, Lye DC. Implementation hurdles of an interactive, integrated, point-of-care computerised decision support system for hospital antibiotic prescription. International journal of antimicrobial agents. 2016 Feb 29;47(2):132-9.

8. Reinhardt JP, Boerner K, Downes D. The Positive Association of End-of-Life Treatment Discussions and Care Satisfaction in the Nursing Home. Journal of social work in end-of-life & palliative care. 2015 Oct 2;11(3-4):307-22.

9. McNulty CA, Lecky DM, Hawking MK, Quigley A, Butler CC. Delayed/back up antibiotic prescriptions: what do the public think?. BMJ open. 2015 Nov 1;5(11):e009748.

10. Paño-Pardo JR, Schüffelmann-Gutiérrez C, Escosa-García L, Laplaza-González M, Moreno-Ramos F, Gómez-Gil R, López JD, Jordán I, Téllez C, de la Oliva P. Opportunities to improve antimicrobial use in paediatric intensive care units: a nationwide survey in Spain. Clinical Microbiology and Infection. 2016 Feb 29;22(2):171-7.

11. Trevino SE, Babcock HM, Henderson JP, Lane MA, Beekmann SE, Polgreen PM, Marschall J. Perceptions and behaviours of infectious diseases physicians when managing urinary tract infections due to MDR organisms. Journal of Antimicrobial Chemotherapy. 2015 Sep 7:dkv271.

12. Arlen AM, Cooper CS. Controversies in the management of vesicoureteral reflux. Current urology reports. 2015 Sep 1;16(9):1-6.

13. Hansen MP, Howlett J, Del Mar C, Hoffmann TC. Parents’ beliefs and knowledge about the management of acute otitis media: a qualitative study. BMC family practice. 2015 Jul 7;16(1):82.

14. Dersch R, Freitag MH, Schmidt S, Sommer H, Rauer S, Meerpohl JJ. Efficacy and safety of pharmacological treatments for acute Lyme neuroborreliosis–a systematic review. European journal of neurology. 2015 Sep 1;22(9):1249-59.

15. Li HK, Agweyu A, English M, Bejon P. An unsupported preference for intravenous antibiotics. PLoS Med. 2015 May 19;12(5):e1001825.

16. Mohamed AF, Johnson FR, Balp MM, Calado F. Preferences and stated adherence for antibiotic treatment of cystic fibrosis pseudomonas infections. The Patient-Patient-Centered Outcomes Research. 2016 Feb 1;9(1):59-67.

17. Shah N, Castro-Sánchez E, Charani E, Drumright LN, Holmes AH. Towards changing healthcare workers' behaviour: a qualitative study exploring non-compliance through appraisals of infection prevention and control practices. Journal of Hospital Infection. 2015 Jun 30;90(2):126-34.

18. Wetzel RJ, Minhas SV, Patrick BC, Janicki JA. Current practice in the management of type I open fractures in children: a survey of POSNA membership. Journal of Pediatric Orthopaedics. 2015 Oct 1;35(7):762-8.

19. Llor C, Bjerrum L, Munck A, Cots JM, Hernández S, Moragas A. Access to point-of-care tests reduces the prescription of antibiotics among antibiotic-requesting subjects with respiratory tract infections. Respiratory care. 2014 Dec 1;59(12):1918-23.

20. Chow A, Lye DC, Arah OA. Psychosocial determinants of physicians’ acceptance of recommendations by antibiotic computerised decision support systems: a mixed methods study. International journal of antimicrobial agents. 2015 Mar 31;45(3):295-304.

21. Fleming-Dutra KE, Shapiro DJ, Hicks LA, Gerber JS, Hersh AL. Race, otitis media, and antibiotic selection. Pediatrics. 2014 Nov 1:peds-2014.

22. Broniatowski DA, Klein EY, Reyna VF. Germs are germs, and why not take a risk? Patients’ expectations for prescribing antibiotics in an inner-city emergency department. Medical Decision Making. 2015 Jan;35(1):60-7.

23. Mitchell SL, Shaffer ML, Loeb MB, Givens JL, Habtemariam D, Kiely DK, D’Agata E. Infection management and multidrug-resistant organisms in nursing home residents with advanced dementia. JAMA internal medicine. 2014 Oct 1;174(10):1660-7.

24. Schroeder AR, Ralston SL. Intravenous antibiotic durations for common bacterial infections in children: When is enough enough?. Journal of hospital medicine. 2014 Sep 1;9(9):604-9.

25. Spiekerman K.M., Patel S.J., Patel R., Kociolek L.K. Availability, perceptions, and characteristics of antibiograms among Illinois pediatricians. Infection and Drug Resistance. 9 (pp 269-274), 2016. Date of Publication: 05 Dec 2016

26. Scales K., Zimmerman S., Reed D., Beeber A.S., Kistler C.E., Preisser J.S., Weiner B.J., Ward K., Fann A., Sloane P.D. Nurse and Medical Provider Perspectives on Antibiotic Stewardship in Nursing Homes. Journal of the American Geriatrics Society. 65 (1) (pp 165-171), 2017. Date of Publication: 01 Jan 2017.

27. Ruta DJ, Kadakia AR, Irwin TA. What are the patterns of prophylactic postoperative oral antibiotic use after foot and ankle surgery?. Clinical Orthopaedics and Related Research. 2014 Oct 1;472(10):3204-13.

28. Rosati P, Di Salvo V, Crudo S, D'amico R, Carlino C, Marchili MR, Gonfiantini M, Di Ciommo V. Are parents of children hospitalized with severe community‐acquired pneumonia more satisfied with care when physicians allow them to share decisions on the antibiotic route?. Health Expectations. 2015 Dec 1;18(6):2278-87.

29. Nussenblatt V, Avdic E, Berenholtz S, Daugherty E, Hadhazy E, Lipsett PA, Maragakis LL, Perl TM, Speck K, Swoboda SM, Ziai W. Ventilator-associated pneumonia: overdiagnosis and treatment are common in medical and surgical intensive care units. Infection Control & Hospital Epidemiology. 2014 Mar 1;35(03):278-84.

30. Mustafa M, Wood F, Butler CC, Elwyn G. Managing expectations of antibiotics for upper respiratory tract infections: a qualitative study. The Annals of Family Medicine. 2014 Jan 1;12(1):29-36.

31. Elemraid MA, Rushton SP, Thomas MF, Spencer DA, Eastham KM, Gennery AR, Clark JE. Changing clinical practice: management of paediatric community‐acquired pneumonia. Journal of evaluation in clinical practice. 2014 Feb 1;20(1):94-9.

32. Forsman J, Anani N, Eghdam A, Falkenhav M, Koch S. Integrated information visualization to support decision making for use of antibiotics in intensive care: design and usability evaluation. Informatics for Health and Social Care. 2013 Dec 1;38(4):330-53.

33. Coenen S, Michiels B, Renard D, Denekens J, Van Royen P. Antibiotic prescribing for acute cough: the effect of perceived patient demand. Br J Gen Pract. 2006 Mar 1;56(524):183-90.

34. Blommaert A, Coenen S, Gielen B, Goossens H, Hens N, Beutels P. Patient and prescriber determinants for the choice between amoxicillin and broader-spectrum antibiotics: a nationwide prescription-level analysis. Journal of antimicrobial chemotherapy. 2013 May 16;68(10):2383-92.

35. Daneman N, Gruneir A, Bronskill SE, Newman A, Fischer HD, Rochon PA, Anderson GM, Bell CM. Prolonged antibiotic treatment in long-term care: role of the prescriber. JAMA internal medicine. 2013 Apr 22;173(8):673-82.

36. Marschall J, Lane MA, Beekmann SE, Polgreen PM, Babcock HM. Current management of prosthetic joint infections in adults: results of an Emerging Infections Network survey. International journal of antimicrobial agents. 2013 Mar 31;41(3):272-7.

37. Velasco E, Noll I, Espelage W, Ziegelmann A, Krause G, Eckmanns T. A survey of outpatient antibiotic prescribing for cystitis. Deutsches Ärzteblatt International. 2012 Dec;109(50):878.

38. Sung L, Regier DA. Decision making in pediatric oncology: Evaluation and incorporation of patient and parent preferences. Pediatric blood & cancer. 2013 Apr 1;60(4):558-63.

39. Diorio C, Tomlinson D, Boydell KM, Regier DA, Ethier MC, Alli A, Alexander S, Gassas A, Taylor J, Kellow C, Mills D. Attitudes toward infection prophylaxis in pediatric oncology: a qualitative approach. PloS one. 2012 Oct 24;7(10):e47815.

40. Regier DA, Diorio C, Ethier MC, Alli A, Alexander S, Boydell KM, Gassas A, Taylor J, Kellow C, Mills D, Sung L. Discrete choice experiment to evaluate factors that influence preferences for antibiotic prophylaxis in pediatric oncology. PloS one. 2012 Oct 17;7(10):e47470.

41. Shmilev TI, Yankov IV. Ventilator-associated pneumonias in children (ii)-prophylaxis and treatment. Folia medica. 2012 Oct 1;54(1):12-8.

42. Ulvik Ø, Ulvik NM. Diversity in urologists' personal preferences in the ureteroscopic management of ureteral calculi in Norway. Scandinavian journal of urology. 2013 Apr 1;47(2):126-30.

43. Hasan K, Racano A, Deheshi B, Farrokhyar F, Wunder J, Ferguson P, Holt G, Schwartz H, Petrisor B, Bhandari M, Ghert M. Prophylactic antibiotic regimens in tumor surgery (PARITY) survey. BMC musculoskeletal disorders. 2012 Jun 7;13(1):91.

44. Coenen S. Infectious diseases in primary care; managing the interface between the person and the community. The European journal of general practice. 2012 Jun 1;18(2):117-21.

45. Sung L, Alibhai SM, Ethier MC, Teuffel O, Cheng S, Fisman D, Regier DA. Discrete choice experiment produced estimates of acceptable risks of therapeutic options in cancer patients with febrile neutropenia. Journal of clinical epidemiology. 2012 Jun 30;65(6):627-34.

46. KwON HH, Yoon HS, Suh DH, Yoon JY, Park SK, Lee ES, Lee JH, Kim NI, Kye YC, Ro YS, Lee SJ. A nationwide study of acne treatment patterns in Korea: analysis of patient preconceived notions and dermatologist suggestion for treatment. Acta dermato-venereologica. 2012 May 15;92(3):236-40.

47. Han DC, Chee SP. Survey of practice preference pattern in antibiotic prophylaxis against endophthalmitis after cataract surgery in Singapore. International ophthalmology. 2012 Apr 1;32(2):127-34.

48. Bamford KB, Desai M, Aruede MJ, Lawson W, Jacklin A, Franklin BD. Patients' views and experience of intravenous and oral antimicrobial therapy: room for change. Injury. 2011 Dec 1;42:S24-7.

49. Hickman SE, Nelson CA, Moss AH, Tolle SW, Perrin NA, Hammes BJ. The Consistency Between Treatments Provided to Nursing Facility Residents and Orders on the Physician Orders for Life‐Sustaining Treatment Form. Journal of the American Geriatrics Society. 2011 Nov 1;59(11):2091-9.

50. García C, Llamocca LP, García K, Jiménez A, Samalvides F, Gotuzzo E, Jacobs J. Knowledge, attitudes and practice survey about antimicrobial resistance and prescribing among physicians in a hospital setting in Lima, Peru. BMC clinical pharmacology. 2011 Nov 15;11(1):18.

51. Tonkin-Crine S, Yardley L, Coenen S, Fernandez-Vandellos P, Krawczyk J, Touboul P, Verheij T, Little P. GPs' views in five European countries of interventions to promote prudent antibiotic use. Br J Gen Pract. 2011 May 1;61(586):e252-61.

52. Zehtabchi S, Yadav K, Brothers E, Khan F, Singh S, Wilcoxson RD, Malhotra S. Prophylactic antibiotics for simple hand lacerations: time for a clinical trial?. Injury. 2012 Sep 30;43(9):1497-501.

53. Jakobsen KA, Melbye H, Kelly MJ, Ceynowa C, Mölstad S, Hood K, Butler CC. Influence of CRP testing and clinical findings on antibiotic prescribing in adults presenting with acute cough in primary care. Scandinavian journal of primary health care. 2010 Dec 1;28(4):229-36.

54. Cals JW, Ament AJ, Hood K, Butler CC, Hopstaken RM, Wassink GF, Dinant GJ. C‐reactive protein point of care testing and physician communication skills training for lower respiratory tract infections in general practice: economic evaluation of a cluster randomized trial. Journal of evaluation in clinical practice. 2011 Dec 1;17(6):1059-69.

55. Minen MT, Duquaine D, Marx MA, Weiss D. A survey of knowledge, attitudes, and beliefs of medical students concerning antimicrobial use and resistance. Microbial Drug Resistance. 2010 Dec 1;16(4):285-9.

56. Berry DJ, Bozic KJ. Current practice patterns in primary hip and knee arthroplasty among members of the American Association of Hip and Knee Surgeons. The Journal of arthroplasty. 2010 Sep 30;25(6):2-4.

57. Hsieh MH, Wildenfels P, Gonzales ET. Surgical antibiotic practices among pediatric urologists in the United States. Journal of pediatric urology. 2011 Apr 30;7(2):192-7.

58. Mousquès J, Renaud T, Scemama O. Is the “practice style” hypothesis relevant for general practitioners? An analysis of antibiotics prescription for acute rhinopharyngitis. Social science & medicine. 2010 Apr 30;70(8):1176-84.

59. Chang DF, Braga-Mele R, Mamalis N, Masket S, Miller KM, Nichamin LD, Packard RB, Packer M, ASCRS Cataract Clinical Committee. Prophylaxis of postoperative endophthalmitis after cataract surgery: results of the 2007 ASCRS member survey. Journal of Cataract & Refractive Surgery. 2007 Oct 31;33(10):1801-5.

60. Cameron ST, Melvin L, Glasier A, Scott G, Johnstone A, Young H. Willingness of gynaecologists, doctors in family planning, GPs, practice nurses and pharmacists to adopt novel interventions for treating sexual partners of women with chlamydia. BJOG: An International Journal of Obstetrics & Gynaecology. 2007 Dec 1;114(12):1516-21.

61. McGregor JC, Harris AD, Furuno JP, Bradham DD, Perencevich EN. Relative influence of antibiotic therapy attributes on physician choice in treating acute uncomplicated pyelonephritis. Medical Decision Making. 2007 Jul;27(4):387-94.

62. Vercillo M, Patzakis MJ, Holtom P, Zalavras CG. Linezolid in the treatment of implant-related chronic osteomyelitis. Clinical orthopaedics and related research. 2007 Aug 1;461:40-3.

63. Jenkins J, Shields M, Patterson C, Kee F. Decision making in asthma exacerbation–a clinical judgement analysis. Archives of disease in childhood. 2007 Apr 11.

64. Miravitlles M, Llor C. Determining factors in the prescription of moxifloxacin in exacerbations of chronic bronchitis in the primary-care setting. Clinical drug investigation. 2007 Feb 1;27(2):95-104.

65. Sheeder J, Stevens-Simon C, Lezotte D, Glazner J, Scott S. Cervicitis: to treat or not to treat? The role of patient preferences and decision analysis. Journal of adolescent health. 2006 Dec 31;39(6):887-92.

66. Sanabria A. Decision-making analysis for selection of antibiotic treatment in intra-abdominal infection using preference measurements. Surgical infections. 2006 Oct 1;7(5):453-62.

67. Chenevier DG, LeLorier J. A willingness-to-pay assessment of parents’ preference for shorter duration treatment of acute otitis media in children. Pharmacoeconomics. 2005 Dec 1;23(12):1243-55.

68. Merenstein D, Diener-West M, Krist A, Pinneger M, Cooper LA. An assessment of the shared-decision model in parents of children with acute otitis media. Pediatrics. 2005 Dec 1;116(6):1267-75.

69. Reinbolt RE, Shenk AM, White PH, Navari RM. Symptomatic treatment of infections in patients with advanced cancer receiving hospice care. Journal of pain and symptom management. 2005 Aug 31;30(2):175-82.

70. Oliveira AG. Current management of hospitalized community acquired pneumonia in Portugal. Consensus statements of an expert panel. Revista portuguesa de pneumologia. 2004 Dec;11(3):243-82.

71. Brinsley KJ, Sinkowitz-Cochran RL, Cardo DM, CDC Campaign to Prevent Antimicrobial Resistance Team. Assessing motivation for physicians to prevent antimicrobial resistance in hospitalized children using the Health Belief Model as a framework. American journal of infection control. 2005 Apr 30;33(3):175-81.

72. Deuster S, Roten I, Muehlebach S. Implementation of treatment guidelines to support judicious use of antibiotic therapy. Journal of clinical pharmacy and therapeutics. 2010 Feb 1;35(1):71-8.

73. David RD, DeBlieux PM, Press R. Rational antibiotic treatment of outpatient genitourinary infections in a changing environment. The American journal of medicine. 2005 Jul 31;118(7):7-13.

74. McIsaac WJ, Prakash P, Ross S. The management of acute uncomplicated cystitis in adult women by family physicians in Canada. Canadian Journal of Infectious Diseases and Medical Microbiology. 2008;19(4):287-93.

75. Sabuda DM, Clark C, Pattullo AL. Retrospective audit of prophylactic use of antibiotic-containing bone cement for primary total hip and knee arthroplasty in the Calgary Health Region. The Canadian Journal of Hospital Pharmacy. 2008;61(5).

76. Jin C, Ely A, Fang L, Liang X. Framing a global health risk from the bottom-up: user perceptions and practices around antibiotics in four villages in China. Health, risk & society. 2011 Aug 1;13(5):433-49.

77. Ng RS, Chong CP. Surgeons' adherence to guidelines for surgical antimicrobial prophylaxis–a review. The Australasian medical journal. 2012;5(10):534.

78. Ullah H, Khan SA, Bakht SM, Tehseen Y, Karim S, Baseer A, Murtaza G. Prescribing Practice of Antibiotics Amongst Children; Physicians' Behavior and Knowledge. Latin American Journal of Pharmacy. 2012 Jan 1;31(9):1367-9.

79. Forrest CB, Fiks AG, Bailey LC, Localio R, Grundmeier RW, Richards T, Karavite DJ, Elden L, Alessandrini EA. Improving adherence to otitis media guidelines with clinical decision support and physician feedback. Pediatrics. 2013 Apr 1;131(4):e1071-81.

80. Khan AA, Rashed MR, Muneersha TM, Rahiman OF. Assessment of the Prescribing Knowledge, Attitude and Skills of Medical Students and Interns in a Large Teaching Hospital of Southern India. Biomedical and Pharmacology Journal. 2013; 6(1): 63-69.

81. Kandeel A, El-Shoubary W, Hicks LA, Fattah MA, Dooling KL, Lohiniva AL, Ragab O, Galal R, Talaat M. Patient attitudes and beliefs and provider practices regarding antibiotic use for acute respiratory tract infections in Minya, Egypt. Antibiotics. 2014 Nov 14;3(4):632-44.

82. Pasay DK, Chow SJ, Bresee LC, Guirguis M, Slobodan J. Assessment of current antimicrobial stewardship policies and resources: a focus group project. Healthcare infection. 2015 Mar 1;20(1):7-15.

83. Tran G.N., Bodapati A.V., Routh J.C., Saigal C.S., Copp H.L. Parental Preference Assessment for Vesicoureteral Reflux Management in Children. Journal of Urology. (no pagination), 2017. Date of Publication: 2017.

84. Greenberg J., Palmer J.B., Chan W.W., Correia C.E., Whalley D., Shannon P., Sawicki G.S. Treatment satisfaction in cystic fibrosis: Early patient experience with tobramycin inhalation powder. Patient Preference and Adherence. 10 (pp 2163-2169), 2016. Date of Publication: 26 Oct 2016.

85. Pea F. Antimicrobial treatment of bacterial infections in frail elderly patients: The difficult balance between efficacy, safety and tolerability. Current Opinion in Pharmacology. 24 (pp 18-22), 2015. Date of Publication: 01 Jul 2015.

86. Bielicki J.A., Barker C.I.S., van der Velden A.W., Sharland M., van Esso D., Hadjipanayis A., del Torso S., Grossman Z. Antibiotic preferences for childhood pneumonia vary by physician type and European region. ERS Monograph. 2 (2) (no pagination), 2016. Article Number: 00001-2016

87. Chouinard A.-F., Troulis M.J., Lahey E.T. The Acute Management of Facial Fractures. Current Trauma Reports. 2 (2) (pp 55-65), 2016. Date of Publication: 01 Jun 2016.

88. Davis T.D., Rushton H.G. Managing Vesicoureteral Reflux in the Pediatric Patient: a Spectrum of Treatment Options for a Spectrum of Disease. Current Treatment Options in Pediatrics. 2 (1) (pp 23-34), 2016. Date of Publication: 01 Mar 2016.

89. What are the Patterns of Prophylactic Postoperative Oral Antibiotic Use After Foot and Ankle Surgery? Ruta D.J., Kadakia A.R., Irwin T.A. Clinical Orthopaedics and Related Research. (no pagination), 2014. Date of Publication: 19 Jun 2014.

90. Juthani-Mehta M., Malani P.N., Mitchell S.L. Antimicrobials at the End of Life: An Opportunity to Improve Palliative Care and Infection Management. JAMA - Journal of the American Medical Association. 314 (19) (pp 2017-2018) 17 Nov 2015

91. Tonkin-Crine S, Yardley L, Little P. Antibiotic prescribing for acute respiratory tract infections in primary care: a systematic review and meta-ethnography. Journal of antimicrobial chemotherapy. 2011 Jul 15;66(10):2215-23.

92. Petursson P. GPs’ reasons for “non-pharmacological” prescribing of antibiotics - a phenomenological study. Scand J Prim Health 2005; 23: 120–5.

93. Hart AM, Pepper GA, Gonzales R. Balancing acts: deciding for or against antibiotics in acute respiratory infections. J Fam Practice 2006; 55: 320–5.

94. Frich JC, Hoye S, Lindbaek M et al. General practitioners and tutors’ experiences with peer group academic detailing: a qualitative study. BMC Fam Pract 2010; 11: 12. doi:10.1186/1471-2296-11-12.

95. Bekkers MJ, Simpson SA, Dunstan F et al. Enhancing the quality of antibiotic prescribing in primary care: qualitative evaluation of a blended learning intervention. BMC Fam Pract 2010; 11: 34. doi:10.1186/1471-2296-11-34.

96. Cals JWL, Chappin FHF, Hopstaken RM et al. C-reactive protein point-of-care testing for lower respiratory tract infections: a qualitative evaluation of experiences by GPs. Fam Pract 2010; 27: 212–8.

97. Hoye S, Frich JC, Lindbaek M. Delayed prescribing for upper respiratory tract infections: a qualitative study of GPs’ views and experiences. Brit J Gen Pract 2010; 60: 907–12.

98. Coenen S, Francis N, Kelly M, Hood K, Nuttall J, Little P, Verheij TJ, Melbye H, Goossens H, Butler CC, GRACE Project Group. Are patient views about antibiotics related to clinician perceptions, management and outcome? A multi-country study in outpatients with acute cough. PloS one. 2013 Oct 23;8(10):e76691.

99. Rodrigues AT, Roque F, Falcão A, Figueiras A, Herdeiro MT. Understanding physician antibiotic prescribing behaviour: a systematic review of qualitative studies. International journal of antimicrobial agents. 2013 Mar 31;41(3):203-12.

100. Ong S, Nakase J, Moran GJ, Karras DJ, Kuehnert MJ, Talan DA. Antibiotic use for emergency department patients with upper respiratory infections: prescribing practices, patient expectations, and patient satisfaction. Ann Emerg Med 2007;50:213–20.

101. Simpson SA, Wood F, Butler CC. General practitioners’ perceptions of antimicrobial resistance: a qualitative study. J Antimicrob Chemother 2007;59:292–6.

102. Wood F, Simpson S, Butler CC. Socially responsible antibiotic choices in primary care: a qualitative study of GPs’ decisions to prescribe broad-spectrum and fluoroquinolone antibiotics. Fam Pract 2007;24:427–34.

103. Moro ML, Marchi M, Gagliotti C, Di Mario S, Resi D. Why do paediatricians prescribe antibiotics? Results of an Italian regional project. BMC Pediatr 2009;9:69.

104. Bjorkman I, Berg J, Roing M, Erntell M, Lundborg CS. Perceptions among Swedish hospital physicians on prescribing of antibiotics and antibiotic resistance. Qual Saf Health Care 2010;19:e8.

105. Björnsdóttir I, Kristinsson KG, Hansen EH. Diagnosing infections: a qualitative view on prescription decisions in general practice over time. Pharm World Sci 2010;32:805–14.

106. Kotwani A, Wattal C, Katewa S, Joshi PC, Holloway K. Factors influencing primary care physicians to prescribe antibiotics in Delhi India. Fam Pract 2010;27:684–90.

107. Vazquez-Lago JM, Lopez-Vazquez P, Lopez-Duran A, Taracido-Trunk M, Figueiras A. Attitudes of primary care physicians to the prescribing of antibiotics and antimicrobial resistance: a qualitative study from Spain. Fam Pract 2012;29:352–60 [Epub 2011 Oct 19].

108. Zaffani S, Cuzzolin L, Meneghelli G, Gangemi M, Murgia V, Chiamenti G, et al. An analysis of the factors influencing the paediatrician–parents relationship: the importance of the socio-demographic characteristics of the mothers. Child Care Health Dev 2005;31:575–80.

109. Bjorkman I, Erntell M, Roing M, Lundborg CS. Infectious disease management in primary care: perceptions of GPs. BMC Fam Pract 2011;12:1.

110. Reynolds L, McKee M. Factors influencing antibiotic prescribing in China: an exploratory analysis. Health Policy 2009;90:32–6

111. Ong S, Moran GJ, Krishnadasan A, Talan DA. Antibiotic prescribing practices of emergency physicians and patient expectations for uncomplicated lacerations. West J Emerg Med 2011;12:375–80.

112. Midthun S, Paur R, Bruce AW, Midthun P. Urinary tract infections in the elderly: a survey of physicians and nurses. Geriatr Nurs 2005;26:245–51.

NOTES: Reference 33 and references 91–112 added from other sources.

**Summary of Objectives and Context of Included Papers**

**Primary Care papers**

| **Study title** | Delayed/back up antibiotic prescriptions: what do the public think? |
| --- | --- |
| **First author** | McNulty CA |
| **Publication date** | 2015 |
| **Journal** | BMJ open |
| **Paper objectives** | To describe the general public’s understanding, acceptance and use of delayed antibiotics. |
| **Country / countries** | UK (England) |
| **Preference perspective**  **(‘X’ either or both)** |  |
| ***Patients*** |  |
| ***Public*** | X |
| ***Healthcare professionals*** |  |
| **Special Context / other comments** |  |

| **Study title** | Parents’ beliefs and knowledge about the management of acute otitis media: a qualitative study |
| --- | --- |
| **First author** | Hansen MP |
| **Publication date** | 2015 |
| **Journal** | BMC family practice |
| **Paper objectives** | To explore parents’ knowledge and beliefs about the management of acute otitis media in children. |
| **Country / countries** | Australia |
| **Preference perspective**  **(‘X’ either or both)** |  |
| ***Patients*** | X (parents) |
| ***Public*** |  |
| ***Healthcare professionals*** |  |
| **Special Context / other comments** |  |

| **Study title** | Managing expectations of antibiotics for upper respiratory tract infections: a qualitative study |
| --- | --- |
| **First author** | Mustafa M |
| **Publication date** | 2014 |
| **Journal** | The Annals of Family Medicine |
| **Paper objectives** | To explore the views and experiences of family physicians about asking patients directly whether they expect to receive antibiotics for upper respiratory tract infections |
| **Country / countries** | UK (South Wales) |
| **Preference perspective**  **(‘X’ either or both)** |  |
| ***Patients*** | X |
| ***Public*** |  |
| ***Healthcare professionals*** | X |
| **Special Context / other comments** |  |
|  |  |

| **Study title** | Access to point-of-care tests reduces the prescription of antibiotics among antibiotic-requesting subjects with respiratory tract infections |
| --- | --- |
| **First author** | Llor C |
| **Publication date** | 2014 |
| **Journal** | Respiratory care |
| **Paper objectives** | To evaluate the effect of access to point-of-care tests on decreasing the prescription of antibiotics in respiratory tract infections in subjects who  explicitly requested an antibiotic prescription |
| **Country / countries** | Spain |
| **Preference perspective**  **(‘X’ either or both)** | N/A |
| ***Patients*** |  |
| ***Public*** |  |
| ***Healthcare professionals*** |  |
| **Special Context / other comments** |  |

| **Study title** | Patient and prescriber determinants for the choice between amoxicillin and broader-spectrum antibiotics: a nationwide prescription-level analysis |
| --- | --- |
| **First author** | Blommaert A |
| **Publication date** | 2013 |
| **Journal** | JAC |
| **Paper objectives** | Patient and prescriber characteristics that affect broad v narrow spectrum antibiotics |
| **Country / countries** | Belgium |
| **Preference perspective**  **(‘X’ either or both)** |  |
| ***Patients*** | X |
| ***Public*** |  |
| ***Healthcare professionals*** | X |
| **Special Context / other comments** |  |

| **Study title** | Prolonged antibiotic treatment in long-term care: role of the prescriber |
| --- | --- |
| **First author** | Daneman N |
| **Publication date** | 2013 |
| **Journal** | JAMA internal medicine |
| **Paper objectives** | Determining if prescriber preferences play a role in antibiotic prescribing in long term care. |
| **Country / countries** | Canada |
| **Preference perspective**  **(‘X’ either or both)** |  |
| ***Patients*** |  |
| ***Public*** |  |
| ***Healthcare professionals*** | X |
| **Special Context / other comments** |  |

| **Study title** | Infectious diseases in primary care; managing the interface between the person and the community |
| --- | --- |
| **First author** | Coenen S |
| **Publication date** | 2012 |
| **Journal** | The European journal of general practice |
| **Paper objectives** |  |
| **Country / countries** |  |
| **Preference perspective**  **(‘X’ either or both)** |  |
| ***Patients*** |  |
| ***Public*** |  |
| ***Healthcare professionals*** | X |
| **Special Context / other comments** |  |

| **Study title** | A nationwide study of acne treatment patterns in Korea: analysis of patient preconceived notions and dermatologist suggestion for treatment |
| --- | --- |
| **First author** | KwON HH |
| **Publication date** | 2012 |
| **Journal** | Acta dermato-venereologica |
| **Paper objectives** | To explore the association between patients’ beliefs regarding acne and physicians’ suggestion for treatment modality in dermatology clinics |
| **Country / countries** | South Korea |
| **Preference perspective**  **(‘X’ either or both)** |  |
| ***Patients*** | X |
| ***Public*** |  |
| ***Healthcare professionals*** | X |
| **Special Context / other comments** |  |

| **Study title** | GPs' views in five European countries of interventions to promote prudent antibiotic use |
| --- | --- |
| **First author** | Tonkin-Crine S |
| **Publication date** | 2011 |
| **Journal** | Br J Gen Pract |
| **Paper objectives** | To explore GPs’ views and experiences of strategies to promote a more prudent use of antibiotics, across five countries |
| **Country / countries** | Belgium, France, Poland, Spain, and the UK |
| **Preference perspective**  **(‘X’ either or both)** |  |
| ***Patients*** |  |
| ***Public*** |  |
| ***Healthcare professionals*** | X |
| **Special Context / other comments** |  |

| **Study title** | Influence of CRP testing and clinical findings on antibiotic prescribing in adults presenting with acute cough in primary care |
| --- | --- |
| **First author** | Jakobsen KA |
| **Publication date** | 2010 |
| **Journal** | Scandinavian journal of primary health care |
| **Paper objectives** | To determine the independent influence of a point of care test (POCT) for C-reactive protein (CRP) on the prescription of antibiotics in patients with acute cough or symptoms suggestive of LRTI, and how symptoms and chest findings influence the decision to prescribe when the test is and is not used |
| **Country / countries** | Norway, Sweden, and Wales |
| **Preference perspective**  **(‘X’ either or both)** |  |
| ***Patients*** |  |
| ***Public*** |  |
| ***Healthcare professionals*** | X |
| **Special Context / other comments** |  |

| **Study title** | C‐reactive protein point of care testing and physician communication skills training for lower respiratory tract infections in general practice: economic evaluation of a cluster randomized trial |
| --- | --- |
| **First author** | Cals JW |
| **Publication date** | 2010 |
| **Journal** | Journal of evaluation in clinical practice |
| **Paper objectives** | Economic evaluation of GP use of CRP point of care test, GP communication skills training, and both GP use of CRP and communication skills training on antibiotic use for lower respiratory tract infections (LRTIs) |
| **Country / countries** | The Netherlands |
| **Preference perspective**  **(‘X’ either or both)** |  |
| ***Patients*** |  |
| ***Public*** |  |
| ***Healthcare professionals*** | X |
| **Special Context / other comments** |  |

| **Study title** | Is the “practice style” hypothesis relevant for general practitioners? An analysis of antibiotics prescription for acute rhinopharyngitis |
| --- | --- |
| **First author** | Mousquès J |
| **Publication date** | 2010 |
| **Journal** | Social science & medicine. |
| **Paper objectives** | To assess the relevance of hypotheses which assume that physicians adopt a uniform practice style for their patients for each similar clinical decision |
| **Country / countries** | France |
| **Preference perspective**  **(‘X’ either or both)** |  |
| ***Patients*** |  |
| ***Public*** |  |
| ***Healthcare professionals*** | X |
| **Special Context / other comments** |  |

| **Study title** | Willingness of gynaecologists, doctors in family planning, GPs, practice nurses and pharmacists to adopt novel interventions for treating sexual partners of women with chlamydia |
| --- | --- |
| **First author** | Cameron ST |
| **Publication date** | 2007 |
| **Journal** | BJOG: An International Journal of Obstetrics & Gynaecology |
| **Paper objectives** | To determine willingness of health professionals to adopt new interventions for treating sexual partners of women with chlamydia |
| **Country / countries** | UK |
| **Preference perspective**  **(‘X’ either or both)** |  |
| ***Patients*** |  |
| ***Public*** |  |
| ***Healthcare professionals*** | X |
| **Special Context / other comments** | Prescribing antibiotics to be given to partner.  Covers primary and secondary care. |

| **Study title** | Relative influence of antibiotic therapy attributes on physician choice in treating acute uncomplicated pyelonephritis |
| --- | --- |
| **First author** | McGregor JC |
| **Publication date** | 2007 |
| **Journal** | Medical Decision Making |
| **Paper objectives** | DCE to assess relative Influence of Antibiotic Therapy Attributes on Physician Choice in Treating Acute Uncomplicated Pyelonephritis (NB No option to give ‘no antibiotic’ |
| **Country / countries** | USA |
| **Preference perspective**  **(‘X’ either or both)** |  |
| ***Patients*** |  |
| ***Public*** |  |
| ***Healthcare professionals*** | X |
| **Special Context / other comments** |  |

| **Study title** | Determining factors in the prescription of moxifloxacin in exacerbations of chronic bronchitis in the primary-care setting |
| --- | --- |
| **First author** | Miravitlles M |
| **Publication date** | 2007 |
| **Journal** | Clinical drug investigation |
| **Paper objectives** | To describe the characteristics of patients and exacerbations that may determine the choice of an antibacterial in exacerbations of chronic bronchitis |
| **Country / countries** | Spain |
| **Preference perspective**  **(‘X’ either or both)** |  |
| ***Patients*** |  |
| ***Public*** |  |
| ***Healthcare professionals*** | X |
| **Special Context / other comments** |  |

| **Study title** | Cervicitis: to treat or not to treat? The role of patient preferences and decision analysis |
| --- | --- |
| **First author** | Sheeder J |
| **Publication date** | 2006 |
| **Journal** | Journal of adolescent health |
| **Paper objectives** | To examine the positive and negative ramifications of treating cervicitis empirically as a Chlamydial (CT) infection |
| **Country / countries** | USA |
| **Preference perspective**  **(‘X’ either or both)** |  |
| ***Patients*** |  |
| ***Public*** |  |
| ***Healthcare professionals*** | X |
| **Special Context / other comments** |  |

| **Study title** | An assessment of the shared-decision model in parents of children with acute otitis media |
| --- | --- |
| **First author** | Merenstein D |
| **Publication date** | 2005 |
| **Journal** | Pediatrics |
| **Paper objectives** | To assess (1) which style of decision making on the physician’s part would most effectively reduce parents’ proclivity to use antibiotics for treatment of their child’s AOM and (2) parental satisfaction with different doctor-patient decision-making styles |
| **Country / countries** | USA |
| **Preference perspective**  **(‘X’ either or both)** |  |
| ***Patients*** |  |
| ***Public*** | X (parents) |
| ***Healthcare professionals*** |  |
| **Special Context / other comments** |  |

| **Study title** | Rational antibiotic treatment of outpatient genitourinary infections in a changing environment |
| --- | --- |
| **First author** | David RD |
| **Publication date** | 2005 |
| **Journal** | The American journal of medicine |
| **Paper objectives** | To re-evaluate our treatment options for genitourinary infections |
| **Country / countries** | USA |
| **Preference perspective**  **(‘X’ either or both)** |  |
| ***Patients*** | X |
| ***Public*** |  |
| ***Healthcare professionals*** |  |
| **Special Context / other comments** |  |

| **Study title** | The management of acute uncomplicated cystitis in adult women by family physicians in Canada. |
| --- | --- |
| **First author** | McIsaac WJ |
| **Publication date** | 2008 |
| **Journal** | Canadian Journal of Infectious Diseases and Medical Microbiology |
| **Paper objectives** | To assess prescribing patterns and antibiotic preferences of physicians for acute uncomplicated cystitis |
| **Country / countries** | Canada |
| **Preference perspective**  **(‘X’ either or both)** |  |
| ***Patients*** |  |
| ***Public*** |  |
| ***Healthcare professionals*** | X |
| **Special Context / other comments** |  |

| **Study title** | Framing a global health risk from the bottom-up: user perceptions and practices around antibiotics in four villages in China |
| --- | --- |
| **First author** | Jin C |
| **Publication date** | 2011 |
| **Journal** | Health, risk & society |
| **Paper objectives** | To investigate perceptions and practices around antibiotic use amongst villagers in four villages in Hubei and Shandong, China. |
| **Country / countries** | China |
| **Preference perspective**  **(‘X’ either or both)** |  |
| ***Patients*** | X |
| ***Public*** |  |
| ***Healthcare professionals*** | X |
| **Special Context / other comments** |  |

| **Study title** | Improving adherence to otitis media guidelines with clinical decision support and physician feedback |
| --- | --- |
| **First author** | Forrest CB |
| **Publication date** | 2013 |
| **Journal** | Pediatrics |
| **Paper objectives** | To assess the effects of electronic health record–based clinical decision support (CDS) and physician performance feedback on adherence to guidelines for acute otitis media (AOM) and otitis media with effusion (OME). |
| **Country / countries** | USA |
| **Preference perspective**  **(‘X’ either or both)** |  |
| ***Patients*** |  |
| ***Public*** |  |
| ***Healthcare professionals*** | X |
| **Special context / other comments** |  |

| **Study title** | Patient attitudes and beliefs and provider practices regarding antibiotic use for acute respiratory tract infections in Minya, Egypt |
| --- | --- |
| **First author** | Kandeel A |
| **Publication date** | 2014 |
| **Journal** | Antibiotics |
| **Paper objectives** | To explore physician prescribing patterns of antibiotics for acute respiratory infections (ARIs) and to explore the knowledge, attitudes, and practices of patients regarding antibiotic use for ARIs |
| **Country / countries** | Egypt |
| **Preference perspective**  **(‘X’ either or both)** |  |
| ***Patients*** | X |
| ***Public*** |  |
| ***Healthcare professionals*** | X |
| **Special context / other comments** |  |

| **Study title** | Antibiotic prescribing for acute respiratory tract infections in primary care: a systematic review and meta-ethnography |
| --- | --- |
| **First author** | Tonkin-Crine S |
| **Publication date** | 2011 |
| **Journal** | Journal of antimicrobial chemotherapy |
| **Paper objectives** | To examine why some interventions to reduce antibiotic prescribing for acute RTIs may be more effective than others. |
| **Country / countries** | Multi-country |
| **Preference perspective**  **(‘X’ either or both)** |  |
| ***Patients*** |  |
| ***Public*** |  |
| ***Healthcare professionals*** | X |
| **Special context / other comments** |  |

| **Study title** | Understanding the culture of prescribing: qualitative study of general practitioners’ and patients’ perceptions of antibiotics for sore throats |
| --- | --- |
| **First author** | Butler CC |
| **Publication date** | 1998 |
| **Journal** | BMJ |
| **Paper objectives** | To better understand reasons for antibiotics being prescribed for sore throats despite well known evidence that they are generally of little help. |
| **Country / countries** | UK (South Wales) |
| **Preference perspective**  **(‘X’ either or both)** |  |
| ***Patients*** | X |
| ***Public*** |  |
| ***Healthcare professionals*** | X |
| **Special context / other comments** |  |

| **Study title** | Why do general practitioners prescribe antibiotics for sore throat? Grounded theory interview study |
| --- | --- |
| **First author** | Kumar S |
| **Publication date** | 2003 |
| **Journal** | BMJ |
| **Paper objectives** | To understand why general practitioners prescribe antibiotics for some cases of sore throat and to explore the factors that influence their prescribing |
| **Country / countries** | UK |
| **Preference perspective**  **(‘X’ either or both)** |  |
| ***Patients*** |  |
| ***Public*** |  |
| ***Healthcare professionals*** | X |
| **Special context / other comments** |  |

| **Study title** | Intentions, strategies and uncertainty inherent in antibiotic prescribing |
| --- | --- |
| **First author** | Bjornsdottir I |
| **Publication date** | 2002 |
| **Journal** | Eur J Gen Pract |
| **Paper objectives** | To explore physicians’ perceived reasons for deciding to prescribe antibiotics. |
| **Country / countries** | Iceland |
| **Preference perspective**  **(‘X’ either or both)** |  |
| ***Patients*** |  |
| ***Public*** |  |
| ***Healthcare professionals*** | X |
| **Special context / other comments** |  |

| **Study title** | GPs’ reasons for “non-pharmacological” prescribing of antibiotics - a phenomenological study |
| --- | --- |
| **First author** | Petursson P |
| **Publication date** | 2005 |
| **Journal** | Scand J Prim Health |
| **Paper objectives** | To study the reasons cited by Icelandic general practitioners for their “non-pharmacological” prescribing of antibiotics. |
| **Country / countries** | Iceland |
| **Preference perspective**  **(‘X’ either or both)** |  |
| ***Patients*** |  |
| ***Public*** |  |
| ***Healthcare professionals*** | X |
| **Special context / other comments** |  |

| **Study title** | Balancing acts: deciding for or against antibiotics in acute respiratory infections |
| --- | --- |
| **First author** | Hart AM |
| **Publication date** | 2006 |
| **Journal** | J Fam Practice |
| **Paper objectives** | To describe the decision making process in prescribing for acute respiratory infections |
| **Country / countries** | USA |
| **Preference perspective**  **(‘X’ either or both)** |  |
| ***Patients*** |  |
| ***Public*** |  |
| ***Healthcare professionals*** | X |
| **Special context / other comments** |  |

| **Study title** | General practitioners and tutors’ experiences with peer group academic detailing: a qualitative study |
| --- | --- |
| **First author** | Frich JC |
| **Publication date** | 2010 |
| **Journal** | BMC Fam Pract |
| **Paper objectives** | To explore GPs and tutors’ experiences with peer group academic detailing, and to explore GPs’ reasons for deviating from recommended practice (in either supporting a safer prescription practice for elderly patients or for prescribing practice for patients with RTIs) |
| **Country / countries** | Norway |
| **Preference perspective**  **(‘X’ either or both)** |  |
| ***Patients*** |  |
| ***Public*** |  |
| ***Healthcare professionals*** | X |
| **Special context / other comments** |  |

| **Study title** | Enhancing the quality of antibiotic prescribing in primary care: qualitative evaluation of a blended learning intervention |
| --- | --- |
| **First author** | Bekkers MJ |
| **Publication date** | 2010 |
| **Journal** | BMC Fam Pract |
| **Paper objectives** | To report part of the process evaluation of a trial of the Stemming the Tide of Antibiotic Resistance (STAR) Educational Program, which aims to enhance the quality of antibiotic prescribing and raise awareness about antibiotic resistance among general medical practitioners |
| **Country / countries** | UK |
| **Preference perspective**  **(‘X’ either or both)** |  |
| ***Patients*** |  |
| ***Public*** |  |
| ***Healthcare professionals*** | X |
| **Special context / other comments** |  |

| **Study title** | C-reactive protein point-of-care testing for lower respiratory tract infections: a qualitative evaluation of experiences by GPs |
| --- | --- |
| **First author** | Cals JWL |
| **Publication date** | 2010 |
| **Journal** | Fam Pract |
| **Paper objectives** | To explore GPs’ attitudes to and experiences of introducing C-reactive protein (CRP) point-of-care testing (POCT) for lower respiratory tract infections (LRTI) in primary care. |
| **Country / countries** | The Netherlands |
| **Preference perspective**  **(‘X’ either or both)** |  |
| ***Patients*** |  |
| ***Public*** |  |
| ***Healthcare professionals*** | X |
| **Special context / other comments** |  |

| **Study title** | Delayed prescribing for upper respiratory tract infections: a qualitative study of GPs’ views and experiences |
| --- | --- |
| **First author** | Hoye S |
| **Publication date** | 2010 |
| **Journal** | Brit J Gen Pract |
| **Paper objectives** | To explore GPs’ views on and experiences with delayed prescribing in patients with acute upper respiratory tract infections |
| **Country / countries** | Norway |
| **Preference perspective**  **(‘X’ either or both)** |  |
| ***Patients*** |  |
| ***Public*** |  |
| ***Healthcare professionals*** | X |
| **Special context / other comments** |  |

| **Study title** | Are patient views about antibiotics related to clinician perceptions, management and outcome? A multi-country study in outpatients with acute cough |
| --- | --- |
| **First author** | Coenen S |
| **Publication date** | 2013 |
| **Journal** | PloS one |
| **Paper objectives** | To explore whether patient views (expecting, hoping for or asking for antibiotics) are associated with illness presentation and resolution, whether patient views are accurately perceived by clinicians, and the association of all these factors with antibiotic prescribing and patient satisfaction with care |
| **Country / countries** | 13 European countries |
| **Preference perspective**  **(‘X’ either or both)** |  |
| ***Patients*** | X |
| ***Public*** |  |
| ***Healthcare professionals*** | X |
| **Special context / other comments** |  |

| **Study title** | Antibiotic prescribing for acute cough: the effect of perceived patient demand |
| --- | --- |
| **First author** | Coenen S |
| **Publication date** | 2006 |
| **Journal** | Br J Gen Pract |
| **Paper objectives** | To obtain a valid estimate of the effect of perceived patient demand on antibiotic prescribing for acute cough |
| **Country / countries** | Belgium |
| **Preference perspective**  **(‘X’ either or both)** |  |
| ***Patients*** |  |
| ***Public*** |  |
| ***Healthcare professionals*** | X |
| **Special context / other comments** |  |

| **Study title** | Understanding physician antibiotic prescribing behaviour: a systematic review of qualitative studies |
| --- | --- |
| **First author** | Rodrigues AT |
| **Publication date** | 2013 |
| **Journal** | International journal of antimicrobial agents |
| **Paper objectives** | Systematic review of qualitative studies to better understand physician antibiotic prescribing behaviour |
| **Country / countries** | N/A |
| **Preference perspective**  **(‘X’ either or both)** |  |
| ***Patients*** |  |
| ***Public*** |  |
| ***Healthcare professionals*** | X |
| **Special context / other comments** | Primary and secondary care |

| **Study title** | General practitioners’ perceptions of antimicrobial resistance: a qualitative study |
| --- | --- |
| **First author** | Simpson SA |
| **Publication date** | 2007 |
| **Journal** | J Antimicrob Chemother |
| **Paper objectives** | To achieve a deeper understanding of GPs’ perceptions of antimicrobial resistance. |
| **Country / countries** | UK (Wales) |
| **Preference perspective**  **(‘X’ either or both)** |  |
| ***Patients*** |  |
| ***Public*** |  |
| ***Healthcare professionals*** | X |
| **Special context / other comments** |  |

| **Study title** | Socially responsible antibiotic choices in primary care: a qualitative study of GPs’ decisions to prescribe broad-spectrum and fluoroquinolone antibiotics |
| --- | --- |
| **First author** | Wood F |
| **Publication date** | 2007 |
| **Journal** | Fam Pract |
| **Paper objectives** | To explore the reasons for GPs’ choice of prescribed antibiotic, in particular their decision to prescribe fluroquinolones. |
| **Country / countries** | UK (Wales) |
| **Preference perspective**  **(‘X’ either or both)** |  |
| ***Patients*** |  |
| ***Public*** |  |
| ***Healthcare professionals*** | X |
| **Special context / other comments** |  |

| **Study title** | Why do paediatricians prescribe antibiotics? Results of an Italian regional project. |
| --- | --- |
| **First author** | Moro ML |
| **Publication date** | 2009 |
| **Journal** | BMC Pediatr |
| **Paper objectives** | To investigate determinants of antibiotic prescription in paediatric care, as a first step of a multilevel intervention to improve prescribing for common respiratory tract infections (RTIs) in a northern Italian region with high antibiotic prescription rate. |
| **Country / countries** | Italy |
| **Preference perspective**  **(‘X’ either or both)** |  |
| ***Patients*** |  |
| ***Public*** |  |
| ***Healthcare professionals*** |  |
| **Special context / other comments** |  |

| **Study title** | Diagnosing infections: a qualitative view on prescription decisions in general practice over time |
| --- | --- |
| **First author** | Björnsdóttir I |
| **Publication date** | 2010 |
| **Journal** | Pharm World Sci |
| **Paper objectives** | To map general practitioners’ (GPs’) decision-making for common infections, exploring their diagnostic basis for antibiotic prescriptions. |
| **Country / countries** | Iceland |
| **Preference perspective**  **(‘X’ either or both)** |  |
| ***Patients*** |  |
| ***Public*** |  |
| ***Healthcare professionals*** | X |
| **Special context / other comments** |  |

| **Study title** | Factors influencing primary care physicians to prescribe antibiotics in Delhi India |
| --- | --- |
| **First author** | Kotwani A |
| **Publication date** | 2010 |
| **Journal** | Fam Pract |
| **Paper objectives** | To explore the factors that influence primary care physicians to prescribe antibiotics and to investigate possible interventions. |
| **Country / countries** | India |
| **Preference perspective**  **(‘X’ either or both)** |  |
| ***Patients*** |  |
| ***Public*** |  |
| ***Healthcare professionals*** | X |
| **Special context / other comments** |  |

| **Study title** | Attitudes of primary care physicians to the prescribing of antibiotics and antimicrobial resistance: a qualitative study from Spain |
| --- | --- |
| **First author** | Vazquez-Lago JM |
| **Publication date** | 2012 |
| **Journal** | Fam Pract |
| **Paper objectives** | To ascertain the opinions and attitudes of GPs in Spain with respect to antibiotics and resistance. |
| **Country / countries** | Spain |
| **Preference perspective**  **(‘X’ either or both)** |  |
| ***Patients*** |  |
| ***Public*** |  |
| ***Healthcare professionals*** | X |
| **Special context / other comments** |  |

| **Study title** | An analysis of the factors influencing the paediatrician–parents relationship: the importance of the socio-demographic characteristics of the mothers |
| --- | --- |
| **First author** | Zaffani S |
| **Publication date** | 2005 |
| **Journal** | Child Care Health Dev |
| **Paper objectives** | To investigate the influence of socio-demographic characteristics on the relationship between parents and clinicians (paediatricians) |
| **Country / countries** | Italy |
| **Preference perspective**  **(‘X’ either or both)** |  |
| ***Patients*** |  |
| ***Public*** |  |
| ***Healthcare professionals*** |  |
| **Special context / other comments** |  |

| **Study title** | Infectious disease management in primary care: perceptions of GPs |
| --- | --- |
| **First author** | Bjorkman I |
| **Publication date** | 2011 |
| **Journal** | BMC Fam Pract |
| **Paper objectives** | To explore and describe the variations in GPs’ perceptions of infectious disease management, with special reference to antibiotic prescribing. |
| **Country / countries** | Sweden |
| **Preference perspective**  **(‘X’ either or both)** |  |
| ***Patients*** |  |
| ***Public*** |  |
| ***Healthcare professionals*** | X |
| **Special context / other comments** |  |

| **Study title** | Factors influencing antibiotic prescribing in China: an exploratory analysis |
| --- | --- |
| **First author** | Reynolds L |
| **Publication date** | 2009 |
| **Journal** | Health Policy |
| **Paper objectives** | Qualitative study to assess knowledge, attitudes, and practices in relation to the use of antibiotics. |
| **Country / countries** | China |
| **Preference perspective**  **(‘X’ either or both)** |  |
| ***Patients*** | X |
| ***Public*** |  |
| ***Healthcare professionals*** | X |
| **Special context / other comments** | Primary / Secondary care |

| **Study title** | Urinary tract infections in the elderly: a survey of physicians and nurses |
| --- | --- |
| **First author** | Midthun S |
| **Publication date** | 2005 |
| **Journal** | Geriatr Nurs |
| **Paper objectives** | To identify, in the presence of significant bacteriuria, the symptoms that determine, and conditions that affect, whether a physician will begin antibiotic treatment in the elderly |
| **Country / countries** | USA |
| **Preference perspective**  **(‘X’ either or both)** |  |
| ***Patients*** |  |
| ***Public*** |  |
| ***Healthcare professionals*** | X |
| **Special context / other comments** | Primary / Secondary care |

**Secondary, Tertiary & Ambulatory Care papers**

| **Study title** | Antibiotic prescribing practices of emergency physicians and patient expectations for uncomplicated lacerations |
| --- | --- |
| **First author** | Ong S |
| **Publication date** | 2011 |
| **Journal** | West J Emerg Med |
| **Paper objectives** | To evaluate the proportion of patients with uncomplicated lacerations who are prescribed prophylactic antibiotics in the emergency department (ED), factors that physicians considered when prescribing antibiotics, and factors associated with patient satisfaction. |
| **Country / countries** | USA |
| **Preference perspective**  **(‘X’ either or both)** |  |
| ***Patients*** | X |
| ***Public*** |  |
| ***Healthcare professionals*** | X |
| **Special Context / other comments** |  |

| **Study title** | Perceptions among Swedish hospital physicians on prescribing of antibiotics and antibiotic resistance |
| --- | --- |
| **First author** | Bjorkman I |
| **Publication date** | 2010 |
| **Journal** | Qual Saf Health Care |
| **Paper objectives** | To explore and describe perceptions of antibiotic prescribing among Swedish hospital physicians, with special reference to whether the perceptions included awareness of antibiotic resistance |
| **Country / countries** | Sweden |
| **Preference perspective**  **(‘X’ either or both)** |  |
| ***Patients*** |  |
| ***Public*** |  |
| ***Healthcare professionals*** | X |
| **Special Context / other comments** |  |

| **Study title** | Antibiotic use for emergency department patients with upper respiratory infections: prescribing practices, patient expectations, and patient satisfaction |
| --- | --- |
| **First author** | Ong S |
| **Publication date** | 2007 |
| **Journal** | Ann Emerg Med |
| **Paper objectives** | To evaluate the factors that physicians in the emergency department (ED) consider when prescribing antibiotics (eg, patient expectations) and the factors associated with patient satisfaction. |
| **Country / countries** | USA |
| **Preference perspective**  **(‘X’ either or both)** |  |
| ***Patients*** | X |
| ***Public*** |  |
| ***Healthcare professionals*** | X |
| **Special Context / other comments** |  |

| **Study title** | Attitudes and Behaviours to Antimicrobial Prescribing following Introduction of a Smartphone App. |
| --- | --- |
| **First author** | Panesar P |
| **Publication date** | 2016 |
| **Journal** | PLoS ONE |
| **Paper objectives** | To assess the relationship between use of smartphone app prescribing guidance and the attitudes and behaviours of antimicrobial  prescribers |
| **Country / countries** |  |
| **Preference perspective**  **(‘X’ either or both)** |  |
| ***Patients*** |  |
| ***Public*** |  |
| ***Healthcare professionals*** | X |
| **Special Context / other comments** |  |

| **Study title** | Surgeon preferences regarding antibiotic prophylaxis for ballistic fractures |
| --- | --- |
| **First author** | Marecek GS |
| **Publication date** | 2016 |
| **Journal** | Archives of orthopaedic and trauma surgery |
| **Paper objectives** | To define current practice patterns in the orthopaedic trauma community regarding antibiotic use for low velocity ballistic fractures |
| **Country / countries** |  |
| **Preference perspective**  **(‘X’ either or both)** |  |
| ***Patients*** |  |
| ***Public*** |  |
| ***Healthcare professionals*** | X |
| **Special Context / other comments** |  |

| **Study title** | Patient perspectives on antibiotics for appendicitis at one hospital |
| --- | --- |
| **First author** | Kadera SP |
| **Publication date** | 2016 |
| **Journal** | Journal of Surgical Research |
| **Paper objectives** | To survey adults patients, including parents, to determine their understanding of appendicitis, preference for antibiotic or surgical management, and perception of benefits and risks associated with these management strategies, for acute uncomplicated appendicitis |
| **Country / countries** | USA |
| **Preference perspective**  **(‘X’ either or both)** |  |
| ***Patients*** | X |
| ***Public*** |  |
| ***Healthcare professionals*** |  |
| **Special Context / other comments** |  |

| **Study title** | Seventy-two-hour antibiotic retrieval from the ED: a randomized controlled trial of discharge instructional modality |
| --- | --- |
| **First author** | Olives TD |
| **Publication date** | 2016 |
| **Journal** | The American journal of emergency medicine. |
| **Paper objectives** | To examine the impact of instructional modality on 72-hour antibiotic retrieval among ED patients prescribed  outpatient antibiotics for infections. |
| **Country / countries** | USA |
| **Preference perspective**  **(‘X’ either or both)** |  |
| ***Patients*** | X |
| ***Public*** |  |
| ***Healthcare professionals*** |  |
| **Special Context / other comments** |  |

| **Study title** | Identification of patient characteristics influencing setting of care decisions for patients with acute bacterial skin and skin structure infections: results of a discrete choice experiment |
| --- | --- |
| **First author** | Lane S |
| **Publication date** | 2016 |
| **Journal** | Clinical therapeutics |
| **Paper objectives** | To understand the patient attributes that affect clinical decision-making regard- ing the setting of care for ABSSSI treatment. |
| **Country / countries** | UK and USA |
| **Preference perspective**  **(‘X’ either or both)** |  |
| ***Patients*** |  |
| ***Public*** |  |
| ***Healthcare professionals*** | X |
| **Special Context / other comments** |  |

| **Study title** | Implementation hurdles of an interactive, integrated, point-of-care computerised decision support system for hospital antibiotic prescription. |
| --- | --- |
| **First author** | Chow AL |
| **Publication date** | 2016 |
| **Journal** | International journal of antimicrobial agents |
| **Paper objectives** | To describe the implementation hurdles of introducing computerised antibiotic decision support system |
| **Country / countries** | Singapore |
| **Preference perspective**  **(‘X’ either or both)** |  |
| ***Patients*** |  |
| ***Public*** |  |
| ***Healthcare professionals*** | X |
| **Special Context / other comments** |  |

| **Study title** | Opportunities to improve antimicrobial use in paediatric intensive care units: a nationwide survey in Spain |
| --- | --- |
| **First author** | Paño-Pardo JR |
| **Publication date** | 2016 |
| **Journal** | Clinical Microbiology and Infection. |
| **Paper objectives** | 1) To assess those characteristics of Spanish PICUs that  could influence antimicrobial prescribing or antimicrobial stewardship; 2) To assess the attitudes and perceptions of PICU physicians regarding antimicrobial resistance and antimicrobial use. |
| **Country / countries** | Spain |
| **Preference perspective**  **(‘X’ either or both)** |  |
| ***Patients*** |  |
| ***Public*** |  |
| ***Healthcare professionals*** | X |
| **Special Context / other comments** |  |

| **Study title** | Perceptions and behaviours of infectious diseases physicians when managing urinary tract infections due to MDR organisms |
| --- | --- |
| **First author** | Trevino SE |
| **Publication date** | 2015 |
| **Journal** | Journal of Antimicrobial Chemotherapy |
| **Paper objectives** | To attain a better understanding of infectious diseases (ID) physicians’  experience with MDR organism (MDRO) urinary tract infections (UTIs) by using a survey on disease perception, diagnostic management and treatment preferences. |
| **Country / countries** | USA and Canada |
| **Preference perspective**  **(‘X’ either or both)** |  |
| ***Patients*** |  |
| ***Public*** |  |
| ***Healthcare professionals*** | X |
| **Special Context / other comments** |  |

| **Study title** | Controversies in the management of vesicoureteral reflux |
| --- | --- |
| **First author** | Arlen AM |
| **Publication date** | 2015 |
| **Journal** | Current urology reports |
| **Paper objectives** | To provide an overview of controversies, and make recommendations, in the management of vesicoureteral reflux |
| **Country / countries** | USA |
| **Preference perspective**  **(‘X’ either or both)** |  |
| ***Patients*** |  |
| ***Public*** |  |
| ***Healthcare professionals*** | X |
| **Special Context / other comments** |  |

| **Study title** | Efficacy and safety of pharmacological treatments for acute Lyme neuroborreliosis–a systematic review |
| --- | --- |
| **First author** | Dersch R |
| **Publication date** | 2015 |
| **Journal** | European journal of neurology |
| **Paper objectives** | To evaluate the available evidence for pharmacological treatment of acute Lyme neuroborreliosis as a basis for evidence-based clinical recommendations |
| **Country / countries** | N/A |
| **Preference perspective**  **(‘X’ either or both)** | N/A |
| ***Patients*** |  |
| ***Public*** |  |
| ***Healthcare professionals*** |  |
| **Special Context / other comments** |  |

| **Study title** | An unsupported preference for intravenous antibiotics |
| --- | --- |
| **First author** | Li HK |
| **Publication date** | 2015 |
| **Journal** | PLoS Med |
| **Paper objectives** | An essay on the importance of expanding the evidence base |
| **Country / countries** | N/A |
| **Preference perspective**  **(‘X’ either or both)** | N/A |
| ***Patients*** |  |
| ***Public*** |  |
| ***Healthcare professionals*** |  |
| **Special Context / other comments** |  |

| **Study title** | Preferences and stated adherence for antibiotic treatment of cystic fibrosis pseudomonas infections |
| --- | --- |
| **First author** | Mohamed AF |
| **Publication date** | 2016 |
| **Journal** | The Patient-Patient-Centered Outcomes Research |
| **Paper objectives** | Using a DCE to quantify preferences and stated adherence for inhaled antibiotic treatments in cystic fibrosis |
| **Country / countries** | USA |
| **Preference perspective**  **(‘X’ either or both)** |  |
| ***Patients*** | X (adult patients and parents) |
| ***Public*** |  |
| ***Healthcare professionals*** |  |
| **Special Context / other comments** |  |

| **Study title** | Towards changing healthcare workers' behaviour: a qualitative study exploring non-compliance through appraisals of infection prevention and control practices |
| --- | --- |
| **First author** | Shah N |
| **Publication date** | 2015 |
| **Journal** | Journal of Hospital Infection |
| **Paper objectives** | To identify behaviours of HCWs that facilitated non-compliance with IPC practices, focusing on how appraisals of IPC duties and social and environmental circumstances shaped and influenced non-compliant behaviour. |
| **Country / countries** | UK |
| **Preference perspective**  **(‘X’ either or both)** |  |
| ***Patients*** |  |
| ***Public*** |  |
| ***Healthcare professionals*** | X |
| **Special Context / other comments** |  |

| **Study title** | Current practice in the management of type I open fractures in children: a survey of POSNA membership |
| --- | --- |
| **First author** | Wetzel RJ |
| **Publication date** | 2015 |
| **Journal** | Journal of Pediatric Orthopaedics |
| **Paper objectives** | To ascertain the treatment preferences of pediatric orthopaedic surgeons for type I open fractures. |
| **Country / countries** | North America |
| **Preference perspective**  **(‘X’ either or both)** |  |
| ***Patients*** |  |
| ***Public*** |  |
| ***Healthcare professionals*** | X |
| **Special Context / other comments** |  |

| **Study title** | Psychosocial determinants of physicians’ acceptance of recommendations by antibiotic computerised decision support systems: a mixed methods study |
| --- | --- |
| **First author** | Chow A |
| **Publication date** | 2015 |
| **Journal** | International journal of antimicrobial agents |
| **Paper objectives** | To evaluate physicians’ perceptions and attitudes toward antibiotic computerised decision support systems (CDSS) and determine psychosocial factors associated with acceptance of CDSS recommendations for empirical therapy. |
| **Country / countries** | Singapore |
| **Preference perspective**  **(‘X’ either or both)** |  |
| ***Patients*** |  |
| ***Public*** |  |
| ***Healthcare professionals*** | X |
| **Special Context / other comments** |  |

| **Study title** | Race, otitis media, and antibiotic selection |
| --- | --- |
| **First author** | Fleming-Dutra KE |
| **Publication date** | 2014 |
| **Journal** | Pediatrics |
| **Paper objectives** | To determine whether ethnicity is associated with differences in otitis media diagnosis and antibiotic prescribing nationally |
| **Country / countries** | USA |
| **Preference perspective**  **(‘X’ either or both)** |  |
| ***Patients*** |  |
| ***Public*** |  |
| ***Healthcare professionals*** | X |
| **Special Context / other comments** |  |

| **Study title** | Germs are germs, and why not take a risk? Patients’ expectations for prescribing antibiotics in an inner-city emergency department |
| --- | --- |
| **First author** | Broniatowski DA |
| **Publication date** | 2015 |
| **Journal** | Medical Decision Making |
| **Paper objectives** | To investigate two hypotheses: That patients 1) consider “germs are germs” and fail to distinguish between bacteria and viruses; 2) expect antibiotics because they tend to make risky decisions when the status quo is bad |
| **Country / countries** |  |
| **Preference perspective**  **(‘X’ either or both)** |  |
| ***Patients*** | X |
| ***Public*** |  |
| ***Healthcare professionals*** |  |
| **Special Context / other comments** |  |

| **Study title** | Intravenous antibiotic durations for common bacterial infections in children: When is enough enough? |
| --- | --- |
| **First author** | Schroeder AR |
| **Publication date** | 2014 |
| **Journal** | Journal of hospital medicine |
| **Paper objectives** | To propose a framework for the duration of therapy |
| **Country / countries** | USA |
| **Preference perspective**  **(‘X’ either or both)** |  |
| ***Patients*** |  |
| ***Public*** |  |
| ***Healthcare professionals*** | X |
| **Special Context / other comments** |  |

| **Study title** | What are the patterns of prophylactic postoperative oral antibiotic use after foot and ankle surgery? |
| --- | --- |
| **First author** | Ruta DJ |
| **Publication date** | 2014 |
| **Journal** | Clinical Orthopaedics and Related Research |
| **Paper objectives** | To investigate surgeons’ use of prophylactic postoperative oral antibiotics after elective outpatient foot or ankle procedures, identifying (1) frequency  of use, (2) regimen preferences, (3) personal indications, and (4) associated experience and demographics. |
| **Country / countries** | USA |
| **Preference perspective**  **(‘X’ either or both)** |  |
| ***Patients*** |  |
| ***Public*** |  |
| ***Healthcare professionals*** | X |
| **Special Context / other comments** |  |

| **Study title** | Are parents of children hospitalized with severe community‐acquired pneumonia more satisfied with care when physicians allow them to share decisions on the antibiotic route? |
| --- | --- |
| **First author** | Rosati P |
| **Publication date** | 2015 |
| **Journal** | Health Expectations |
| **Paper objectives** | To investigate whether shared decision-making (in choosing the  antibiotic route) influences parental satisfaction |
| **Country / countries** | Italy |
| **Preference perspective**  **(‘X’ either or both)** |  |
| ***Patients*** | X (parents) |
| ***Public*** |  |
| ***Healthcare professionals*** |  |
| **Special Context / other comments** |  |

| **Study title** | Ventilator-associated pneumonia: overdiagnosis and treatment are common in medical and surgical intensive care units |
| --- | --- |
| **First author** | Nussenblatt V |
| **Publication date** | 2014 |
| **Journal** | Infection Control & Hospital Epidemiology |
| **Paper objectives** | To quantify and characterize unjustified antimicrobial use for ventilator associated pneumonia (VAP), and identify risk factors for continuation  of antibiotics in patients without VAP after 3 days |
| **Country / countries** | USA |
| **Preference perspective**  **(‘X’ either or both)** |  |
| ***Patients*** |  |
| ***Public*** |  |
| ***Healthcare professionals*** | X |
| **Special Context / other comments** |  |

| **Study title** | Changing clinical practice: management of paediatric community-acquired  pneumonia |
| --- | --- |
| **First author** | Elemraid MA |
| **Publication date** | 2013 |
| **Journal** | Journal of Evaluation in Clinical Practice |
| **Paper objectives** |  |
| **Country / countries** | UK |
| **Preference perspective**  **(‘X’ either or both)** |  |
| ***Patients*** |  |
| ***Public*** |  |
| ***Healthcare professionals*** | X |
| **Special Context / other comments** |  |

| **Study title** | Integrated information visualization to support decision making for use of antibiotics in intensive care: design and usability evaluation. |
| --- | --- |
| **First author** | Forsman J |
| **Publication date** | 2013 |
| **Journal** | Informatics for Health and Social Care |
| **Paper objectives** | Value of data visualisation tool in ICU |
| **Country / countries** | Sweden |
| **Preference perspective**  **(‘X’ either or both)** |  |
| ***Patients*** |  |
| ***Public*** |  |
| ***Healthcare professionals*** | X |
| **Special Context / other comments** |  |

| **Study title** | Current management of prosthetic joint infections in adults: results of an Emerging Infections Network survey |
| --- | --- |
| **First author** | Marschall J |
| **Publication date** | 2013 |
| **Journal** | International journal of antimicrobial agents |
| **Paper objectives** | Prescriber preferences for management of prosthetic joint infections |
| **Country / countries** | USA and Canada |
| **Preference perspective**  **(‘X’ either or both)** |  |
| ***Patients*** |  |
| ***Public*** |  |
| ***Healthcare professionals*** | X |
| **Special Context / other comments** |  |

| **Study title** | A survey of outpatient antibiotic prescribing for cystitis |
| --- | --- |
| **First author** | Velasco E |
| **Publication date** | 2012 |
| **Journal** | Deutsches Ärzteblatt International |
| **Paper objectives** | Investigate factors that affect the prescribing of specific antibiotics for uncomplicated cystitis in outpatient care. |
| **Country / countries** | Germany |
| **Preference perspective**  **(‘X’ either or both)** |  |
| ***Patients*** |  |
| ***Public*** |  |
| ***Healthcare professionals*** | X |
| **Special Context / other comments** |  |

| **Study title** | Decision making in pediatric oncology: Evaluation and incorporation of patient and parent preferences |
| --- | --- |
| **First author** | Sung L |
| **Publication date** | 2013 |
| **Journal** | Pediatric blood & cancer |
| **Paper objectives** | Evaluation and incorporation of patient and parent preferences |
| **Country / countries** | Canada |
| **Preference perspective**  **(‘X’ either or both)** |  |
| ***Patients*** | X |
| ***Public*** | X (parents) |
| ***Healthcare professionals*** | X |
| **Special Context / other comments** |  |

| **Study title** | Attitudes toward infection prophylaxis in pediatric oncology: a qualitative approach |
| --- | --- |
| **First author** | Diorio C |
| **Publication date** | 2012 |
| **Journal** | PloS one |
| **Paper objectives** | Describing the attitudes of parents, children and healthcare professionals to infection prophylaxis in pediatric oncology |
| **Country / countries** | Canada |
| **Preference perspective**  **(‘X’ either or both)** |  |
| ***Patients*** | X |
| ***Public*** | X (parents) |
| ***Healthcare professionals*** | X |
| **Special Context / other comments** |  |

| **Study title** | Discrete choice experiment to evaluate factors that influence preferences for antibiotic prophylaxis in pediatric oncology |
| --- | --- |
| **First author** | Regier DA |
| **Publication date** | 2012 |
| **Journal** | PloS one |
| **Paper objectives** | Using DCE to quantify impact of attributes influencing preferences of parents and HCPs for antibiotic prophylaxis |
| **Country / countries** | Canada |
| **Preference perspective**  **(‘X’ either or both)** |  |
| ***Patients*** | X |
| ***Public*** | X (parents) |
| ***Healthcare professionals*** | X |
| **Special Context / other comments** |  |

| **Study title** | Ventilator-associated pneumonias in children (ii)-prophylaxis and treatment. Folia medica |
| --- | --- |
| **First author** | Shmilev TI |
| **Publication date** | 2012 |
| **Journal** | Folia medica |
| **Paper objectives** | A review to acquaint pediatricians and neonatologists with the tendencies and the established norms for VAP prevention and treatment |
| **Country / countries** | Not specific |
| **Preference perspective**  **(‘X’ either or both)** |  |
| ***Patients*** |  |
| ***Public*** |  |
| ***Healthcare professionals*** | X |
| **Special Context / other comments** |  |

| **Study title** | Diversity in urologists' personal preferences in the ureteroscopic management of ureteral calculi in Norway |
| --- | --- |
| **First author** | Ulvik Ø |
| **Publication date** | 2013 |
| **Journal** | Scandinavian journal of urology |
| **Paper objectives** | To evaluate diversities in Norwegian urologists' personal preferences in the endoscopic management of ureteral calculi |
| **Country / countries** | Norway |
| **Preference perspective**  **(‘X’ either or both)** |  |
| ***Patients*** |  |
| ***Public*** |  |
| ***Healthcare professionals*** | X |
| **Special Context / other comments** |  |

| **Study title** | Prophylactic antibiotic regimens in tumor surgery (PARITY) survey |
| --- | --- |
| **First author** | Hasan K |
| **Publication date** | 2012 |
| **Journal** | BMC musculoskeletal disorders |
| **Paper objectives** | To examine surgeon preferences in antibiotic prophylaxis in tumour surgery and perceptions about current evidence |
| **Country / countries** |  |
| **Preference perspective**  **(‘X’ either or both)** |  |
| ***Patients*** |  |
| ***Public*** |  |
| ***Healthcare professionals*** | X |
| **Special Context / other comments** |  |

| **Study title** | Discrete choice experiment produced estimates of acceptable risks of therapeutic options in cancer patients with febrile neutropenia |
| --- | --- |
| **First author** | Sung L |
| **Publication date** | 2012 |
| **Journal** | Journal of clinical epidemiology |
| **Paper objectives** | Parental preferences for home oral/IV antibiotics v IV antibiotics in hospital |
| **Country / countries** |  |
| **Preference perspective**  **(‘X’ either or both)** |  |
| ***Patients*** | X (parents) |
| ***Public*** |  |
| ***Healthcare professionals*** |  |
| **Special Context / other comments** |  |

| **Study title** | Survey of practice preference pattern in antibiotic prophylaxis against endophthalmitis after cataract surgery in Singapore |
| --- | --- |
| **First author** | Han DC |
| **Publication date** | 2012 |
| **Journal** | International ophthalmology |
| **Paper objectives** | To determine practice preference of prophylaxis against post-cataract surgery endophthalmitis in Singapore |
| **Country / countries** | Singapore |
| **Preference perspective**  **(‘X’ either or both)** |  |
| ***Patients*** |  |
| ***Public*** |  |
| ***Healthcare professionals*** | X |
| **Special Context / other comments** |  |

| **Study title** | Patients' views and experience of intravenous and oral antimicrobial therapy: room for change |
| --- | --- |
| **First author** | Bamford KB |
| **Publication date** | 2011 |
| **Journal** | Injury |
| **Paper objectives** | Learn about patients’ views or preferences about the route of administration of antimicrobials |
| **Country / countries** | UK |
| **Preference perspective**  **(‘X’ either or both)** |  |
| ***Patients*** |  |
| ***Public*** |  |
| ***Healthcare professionals*** |  |
| **Special Context / other comments** |  |

| **Study title** | Knowledge, attitudes and practice survey about antimicrobial resistance and prescribing among physicians in a hospital setting in Lima, Peru |
| --- | --- |
| **First author** | García C |
| **Publication date** | 2011 |
| **Journal** | BMC clinical pharmacology |
| **Paper objectives** | To evaluate knowledge, attitudes and practices about AMR and AM prescribing among medical doctors in two large public hospitals in Lima, Peru |
| **Country / countries** | Peru |
| **Preference perspective**  **(‘X’ either or both)** |  |
| ***Patients*** |  |
| ***Public*** |  |
| ***Healthcare professionals*** | X |
| **Special Context / other comments** |  |

| **Study title** | Prophylactic antibiotics for simple hand lacerations: time for a clinical trial? |
| --- | --- |
| **First author** | Zehtabchi S |
| **Publication date** | 2011 |
| **Journal** | Injury |
| **Paper objectives** | To assess the need and feasibility of a randomised controlled trial to evaluate the role of prophylactic antibiotics in simple hand lacerations |
| **Country / countries** | USA |
| **Preference perspective**  **(‘X’ either or both)** |  |
| ***Patients*** |  |
| ***Public*** |  |
| ***Healthcare professionals*** | X |
| **Special Context / other comments** |  |

| **Study title** | Implementation of treatment guidelines to support judicious use of antibiotic therapy |
| --- | --- |
| **First author** | Deuster S |
| **Publication date** | 2010 |
| **Journal** | Journal of clinical pharmacy and therapeutics |
| **Paper objectives** | Intervention study using treatment guidelines to try to improve antibiotic therapy by changing prescribing practice. |
| **Country / countries** |  |
| **Preference perspective**  **(‘X’ either or both)** |  |
| ***Patients*** |  |
| ***Public*** |  |
| ***Healthcare professionals*** | X |
| **Special Context / other comments** |  |

| **Study title** | A survey of knowledge, attitudes, and beliefs of medical students concerning antimicrobial use and resistance |
| --- | --- |
| **First author** | Minen MT |
| **Publication date** | 2010 |
| **Journal** | Microbial Drug Resistance |
| **Paper objectives** | To survey medical students’ perceptions and attitudes about their training on antimicrobial use to identify gaps in medical education. |
| **Country / countries** | USA |
| **Preference perspective**  **(‘X’ either or both)** |  |
| ***Patients*** |  |
| ***Public*** |  |
| ***Healthcare professionals*** | X |
| **Special Context / other comments** |  |

| **Study title** | Current practice patterns in primary hip and knee arthroplasty among members of the American Association of Hip and Knee Surgeons |
| --- | --- |
| **First author** | Berry DJ |
| **Publication date** | 2010 |
| **Journal** | The Journal of arthroplasty |
| **Paper objectives** | To determine current practices in primary total hip arthroplasty and total knee arthroplasty |
| **Country / countries** | USA |
| **Preference perspective**  **(‘X’ either or both)** |  |
| ***Patients*** |  |
| ***Public*** |  |
| ***Healthcare professionals*** | X |
| **Special Context / other comments** |  |

| **Study title** | Surgical antibiotic practices among pediatric urologists in the United States |
| --- | --- |
| **First author** | Hsieh MH |
| **Publication date** | 2011 |
| **Journal** | Journal of pediatric urology |
| **Paper objectives** | To examined pediatric urologist preferences for surgical antibiotic use |
| **Country / countries** | USA |
| **Preference perspective**  **(‘X’ either or both)** |  |
| ***Patients*** |  |
| ***Public*** |  |
| ***Healthcare professionals*** | X |
| **Special Context / other comments** |  |

| **Study title** | Prophylaxis of postoperative endophthalmitis after cataract surgery: results of the 2007 ASCRS member survey |
| --- | --- |
| **First author** | Chang DF |
| **Publication date** | 2007 |
| **Journal** | Journal of Cataract & Refractive Surgery |
| **Paper objectives** | To investigate current antibiotic prophylactic practices for cataract surgery |
| **Country / countries** |  |
| **Preference perspective**  **(‘X’ either or both)** |  |
| ***Patients*** |  |
| ***Public*** |  |
| ***Healthcare professionals*** | X |
| **Special Context / other comments** |  |

| **Study title** | Linezolid in the treatment of implant-related chronic osteomyelitis |
| --- | --- |
| **First author** | Vercillo M |
| **Publication date** | 2007 |
| **Journal** | Clinical orthopaedics and related research |
| **Paper objectives** | To evaluate effectiveness of Linezolid in the Treatment of Implant-related Chronic Osteomyelitis |
| **Country / countries** | USA |
| **Preference perspective**  **(‘X’ either or both)** |  |
| ***Patients*** |  |
| ***Public*** |  |
| ***Healthcare professionals*** | X |
| **Special Context / other comments** |  |

| **Study title** | Decision making in asthma exacerbation–a clinical judgement analysis |
| --- | --- |
| **First author** | Jenkins J |
| **Publication date** | 2007 |
| **Journal** | Archives of disease in childhood |
| **Paper objectives** | To compare decisions between practitioners with different training and experience |
| **Country / countries** | UK (Northern Ireland) |
| **Preference perspective**  **(‘X’ either or both)** |  |
| ***Patients*** |  |
| ***Public*** |  |
| ***Healthcare professionals*** | X |
| **Special Context / other comments** | Decision to give systemic corticosteroids (CS) Vs oral antibiotics Vs admission to hospital |

| **Study title** | Decision-making analysis for selection of antibiotic treatment in intra-abdominal infection using preference measurements |
| --- | --- |
| **First author** | Sanabria A |
| **Publication date** | 2006 |
| **Journal** | Surgical infections |
| **Paper objectives** | to determine the best antibiotic regimen for patients with community-acquired abdominal infection, considering both effectiveness and adverse effects |
| **Country / countries** | Colombia |
| **Preference perspective**  **(‘X’ either or both)** |  |
| ***Patients*** |  |
| ***Public*** |  |
| ***Healthcare professionals*** | X |
| **Special context / other comments** |  |

| **Study title** | A willingness-to-pay assessment of parents’ preference for shorter duration treatment of acute otitis media in children |
| --- | --- |
| **First author** | Chenevier DG |
| **Publication date** | 2005 |
| **Journal** | Pharmacoeconomics |
| **Paper objectives** | To assess parental willingness to pay for a shorter course of antibacterial treatment versus conventional antibacterial therapy for acute otitis media |
| **Country / countries** | Canada |
| **Preference perspective**  **(‘X’ either or both)** |  |
| ***Patients*** | X (parents) |
| ***Public*** |  |
| ***Healthcare professionals*** |  |
| **Special context / other comments** |  |

| **Study title** | Current management of hospitalized community acquired pneumonia in Portugal. Consensus statements of an expert panel |
| --- | --- |
| **First author** | Oliveira AG |
| **Publication date** | 2005 |
| **Journal** | Revista portuguesa de pneumologia |
| **Paper objectives** | To assess whether consensus statements of an expert panel are consistent with guidelines |
| **Country / countries** | Portugal |
| **Preference perspective**  **(‘X’ either or both)** |  |
| ***Patients*** |  |
| ***Public*** |  |
| ***Healthcare professionals*** | X |
| **Special context / other comments** |  |

| **Study title** | Assessing motivation for physicians to prevent antimicrobial resistance in hospitalized children using the Health Belief Model as a framework |
| --- | --- |
| **First author** | Brinsley KJ |
| **Publication date** | 2005 |
| **Journal** | American journal of infection control |
| **Paper objectives** | To assess personal perceptions and cues to action related to the prevention of AMR resistance in hospitalized children |
| **Country / countries** | USA |
| **Preference perspective**  **(‘X’ either or both)** |  |
| ***Patients*** |  |
| ***Public*** |  |
| ***Healthcare professionals*** | X |
| **Special context / other comments** |  |

| **Study title** | Retrospective audit of prophylactic use of antibiotic-containing bone cement for primary total hip and knee arthroplasty in the Calgary Health Region |
| --- | --- |
| **First author** | Sabuda DM |
| **Publication date** | 2008 |
| **Journal** | The Canadian Journal of Hospital Pharmacy |
| **Paper objectives** | To determine the proportion of orthopedic surgeons in the Calgary Health Region who used antibioticcontaining bone cement as prophylactic therapy in conjunction with primary hip and knee arthroplasty, to identify which antibiotics were used and in what amounts, and to determine patient-related factors that might have influenced the use of such therapy. |
| **Country / countries** | Canada |
| **Preference perspective**  **(‘X’ either or both)** |  |
| ***Patients*** |  |
| ***Public*** |  |
| ***Healthcare professionals*** | X |
| **Special context / other comments** |  |

| **Study title** | Surgeons' adherence to guidelines for surgical antimicrobial prophylaxis–a review |
| --- | --- |
| **First author** | Ng RS |
| **Publication date** | 2012 |
| **Journal** | The Australasian medical journal |
| **Paper objectives** | To review studies on surgeons’ adherence to surgical antimicrobial prophylaxis guidelines and factors influencing their adherence. A |
| **Country / countries** | Worldwide |
| **Preference perspective**  **(‘X’ either or both)** |  |
| ***Patients*** |  |
| ***Public*** |  |
| ***Healthcare professionals*** | X |
| **Special context / other comments** |  |

| **Study title** | Prescribing Practice of Antibiotics Amongst Children; Physicians' Behavior and Knowledge |
| --- | --- |
| **First author** | Ullah H |
| **Publication date** | 2012 |
| **Journal** | Latin American Journal of Pharmacy |
| **Paper objectives** | To assess physicians’ behaviour and knowledge regarding prescribing antibiotics |
| **Country / countries** | Pakistan |
| **Preference perspective**  **(‘X’ either or both)** |  |
| ***Patients*** |  |
| ***Public*** |  |
| ***Healthcare professionals*** | X |
| **Special context / other comments** |  |

| **Study title** | Assessment of the Prescribing Knowledge, Attitude and Skills of Medical Students and Interns in a Large Teaching Hospital of Southern India |
| --- | --- |
| **First author** | Khan AA |
| **Publication date** | 2013 |
| **Journal** | Biomedical and Pharmacology Journal |
| **Paper objectives** | To assess undergraduate medical students’ and interns’ attitude and knowledge regarding safe prescribing and to examine their prescribing skills. |
| **Country / countries** | India |
| **Preference perspective**  **(‘X’ either or both)** |  |
| ***Patients*** |  |
| ***Public*** |  |
| ***Healthcare professionals*** | X |
| **Special context / other comments** |  |

| **Study title** | Assessment of current antimicrobial stewardship policies and resources: a focus group project |
| --- | --- |
| **First author** | Pasay DK |
| **Publication date** | 2014 |
| **Journal** | Healthcare infection |
| **Paper objectives** | To qualitatively assess frontline pharmacy staff and leadership perspectives on antimicrobial stewardship resources. |
| **Country / countries** | Canada |
| **Preference perspective**  **(‘X’ either or both)** |  |
| ***Patients*** |  |
| ***Public*** |  |
| ***Healthcare professionals*** | X |
| **Special context / other comments** |  |

**End of life care papers**

| **Study title** | Differences in attitudes to end-of-life care among patients, relatives and healthcare professionals |
| --- | --- |
| **First author** | Ang GC |
| **Publication date** | 2016 |
| **Journal** | Singapore medical journal |
| **Paper objectives** | To explore and compared the differences in attitudes toward end-of-life care among patients, relatives and healthcare professionals, including doctors and nurses |
| **Country / countries** | Singapore |
| **Preference perspective**  **(‘X’ either or both)** |  |
| ***Patients*** | X |
| ***Public*** |  |
| ***Healthcare professionals*** | X |
| **Special context / other comments** |  |

| **Study title** | The Positive Association of End-of-Life Treatment Discussions and Care Satisfaction in the Nursing Home |
| --- | --- |
| **First author** | Reinhardt JP |
| **Publication date** | 2015 |
| **Journal** | Journal of social work in end-of-life & palliative care |
| **Paper objectives** | To determine whether family satisfaction with care  in the nursing home may be associated with involvement in EOL  treatment discussions |
| **Country / countries** | USA |
| **Preference perspective**  **(‘X’ either or both)** |  |
| ***Patients*** | X (family) |
| ***Public*** |  |
| ***Healthcare professionals*** |  |
| **Special context / other comments** |  |

| **Study title** | Infection management and multidrug-resistant organisms in nursing home residents with advanced dementia |
| --- | --- |
| **First author** | Mitchell SL |
| **Publication date** | 2014 |
| **Journal** | JAMA internal medicine |
| **Paper objectives** | To (1) describe the occurrence and management of suspected  infectious episodes, specifically whether antimicrobial treatment initiation was appropriate based on consensus guidelines; (2) identify factors associated with appropriate antimicrobial treatment; (3) describe the prevalence and acquisition of MDRO colonization;(4) examine the association between antimicrobial exposure and acquisition |
| **Country / countries** | USA |
| **Preference perspective**  **(‘X’ either or both)** |  |
| ***Patients*** |  |
| ***Public*** |  |
| ***Healthcare professionals*** |  |
| **Special context / other comments** |  |

| **Study title** | The Consistency Between Treatments Provided to Nursing Facility Residents and Orders on the Physician Orders for Life‐Sustaining Treatment Form |
| --- | --- |
| **First author** | Hickman SE |
| **Publication date** | 2011 |
| **Journal** | Journal of the American Geriatrics Society |
| **Paper objectives** | To evaluate the consistency between treatments provided and Physician Orders for Life-Sustaining Treatment (POLST) orders |
| **Country / countries** | USA |
| **Preference perspective**  **(‘X’ either or both)** |  |
| ***Patients*** | X |
| ***Public*** |  |
| ***Healthcare professionals*** | X |
| **Special context / other comments** |  |

| **Study title** | Symptomatic treatment of infections in patients with advanced cancer receiving hospice care |
| --- | --- |
| **First author** | Reinbolt RE |
| **Publication date** | 2005 |
| **Journal** | Journal of pain and symptom management |
| **Paper objectives** | To determine if the use of antimicrobials for a clinically suspected infection improved infection-related symptoms |
| **Country / countries** | USA |
| **Preference perspective**  **(‘X’ either or both)** |  |
| ***Patients*** | X |
| ***Public*** |  |
| ***Healthcare professionals*** | X |
| **Special context / other comments** |  |
