# Supplementary material for: Why do hospital prescribers continue antibiotics when it is safe to stop? Results of a choice experiment survey
Source: BMC Med. 2020 Jul 30;18:196. doi: 10.1186/s12916-020-01660-4 (PMC7391515; doi:10.1186/s12916-020-01660-4)
Supplement: Supplementary file 2 — Additional file 2: Experimental design. [file 12916_2020_1660_MOESM2_ESM.docx]

**Additional file 2: Experimental design**

The aim of the choice experiment was to model the probability that a respondent would select ‘continue antibiotic treatment’. The probability of choosing this alternative is determined by the indirect utility (*V*). Assuming that utility is linear and additive (a common assumption):

*V* = α + β_1_.SYMPTOMS1 + β_2_. SYMPTOMS2 + β_3_.CONFLICT1 + β_4_. CONFLICT 2 + β_5_.CONTINUERISK + β_6_.STOPRISK + β_7_.PREMORBID1 + β_8_. PREMORBID2 + β_9_.PRESSURE1 + β_10_.PRESSURE2 + γ.Z + ε

Where: α = estimate for the constant

β_1_ to β_10_ = attribute coefficients

Z = set of covariate terms of interest

γ = set of estimates for these covariate terms

ε = error term (unobservable random component)

Expected impact of attributes on utility (from continuing antibiotics)

| **Attribute** | **Expected impact on utility from continuing antibiotics (of an increase in the attribute)** | **Explanation** |
| --- | --- | --- |
| SYMPTOMS | Negative | The levels were ordered by decreasing probability of bacterial infection, so a negative impact (on probability of continuing antibiotics) was expected. |
| CONFLICT | Negative | The levels were ordered by decreasing extent of conflict with discontinuing antibiotics, so a negative impact (on probability of continuing antibiotics) was expected. |
| CONTINUE RISK | Positive | The levels were ordered by decreasing risk of harm from continuing antibiotics, so a positive impact (on probability of continuing antibiotics) was expected. |
| STOP RISK | Negative | The levels were ordered by decreasing risk of harm from stopping antibiotics, so a negative impact (on probability of continuing antibiotics) was expected. |
| PREMORBID | Unclear | The levels were ordered by improving premorbid health, but the impact on probability of continuing antibiotics was unclear. |
| PRESSURE | Positive | The levels were ordered by increasing extent of pressure to continue antibiotic treatment, so a positive impact (on probability of continuing antibiotics) was expected. |

One constraint was applied. If the patient’s presenting symptoms in a scenario were UTI & kidney pain, then we did not allow the possibility that early discontinuation of antibiotics would ‘not conflict’ with guidelines. This was because the words ‘kidney pain’ would suggest that this is not a simple UTI. Guidelines would not encourage early discontinuation in people like this. Additionally, there would be a possible ethical concern if we appeared to imply that such a patient is a possible candidate for early discontinuation.

No potential interactions between attribute-levels were identified.

Ngene was used to generate a d-efficient experimental design for the pilot study. The selected design minimised the d-error for a logistic model. The levels of all attributes were balanced, except for ‘SYMPTOMS’ and ‘CONFLICT’. However, lack of balance in these attributes was due to the constraint we applied. Short of being completely balanced, these two attributes were as close to be being balanced as possible.

The choices respondents made in the pilot study were used as inputs into a model of choice behaviour. The aim of this model was to gain some preliminary knowledge about the strength and direction of respondents’ preferences that could be used to refine the experimental design of the main survey. This was done using conditional logistic regression analysis. The results generated by the pilot model were generally logical and consistent with prior expectations and it had an excellent fit (McFadden Pseudo R-Sq 0.3954). It was therefore judged to be reasonable to take forward as much information as possible from the pilot model to inform the main experimental design.

To do this, we used a model averaging design that gave equal weights to the following two designs:

1. The same model as for the pilot survey, but with attribute priors specified for the three attributes with significant coefficients in the pilot model (Symptoms1 (UTI & Kidney); Continue risk; Stop risk). These priors were set equal to their respective coefficients in the pilot model.
2. The same model as for the pilot survey, but with attribute priors specified for all attributes except for the ‘external pressure to continue’ attribute. Priors for the latter attribute-levels were set to 0 as before. This was because, though small and insignificant, the impacts of the ‘external pressure to continue’ coefficients on the probability of continuing antibiotics were not in the expected directions.

The choices generated by this experimental design are presented in Additional file 3.
