# Supplementary material for: Why do hospital prescribers continue antibiotics when it is safe to stop? Results of a choice experiment survey
Source: BMC Med. 2020 Jul 30;18:196. doi: 10.1186/s12916-020-01660-4 (PMC7391515; doi:10.1186/s12916-020-01660-4)
Supplement: Supplementary file 3 — Additional file 3: Full text of final choice experiment survey. [file 12916_2020_1660_MOESM3_ESM.docx]

# Additional file 3: Full text of final choice experiment survey

**PARTICIPANT INFORMATION**

**A survey study to better understand healthcare workers’ views on starting and stopping antibiotics in the treatment of acute medical patients in secondary care**

We would like to invite you to take part in our survey study. The survey is being run by the [Nuffield Department of Population Health](https://www.ndph.ox.ac.uk/) at the University of Oxford. Before you decide whether or not to participate in this study, it is important that you understand why the research is being carried out and what it would involve for you. Please take the time to read this information, and discuss it with others if you wish. If there is anything that is not clear, or if you would like more information, please ask us. Our contact details are at the bottom of this webpage.

**What is the purpose of the study?**

This survey concerns the treatment of acute medical patients in secondary care with antibiotics. Current Department of Health guidelines recommend that antibiotic prescriptions are reviewed and revised within 72 hours of treatment initiation in hospitalised patients. We want to understand how healthcare workers reviewing antibiotic prescriptions decide whether treatment should be continued or discontinued. This information may help inform future clinical practice.

**Why have I been invited?**

You have been invited to participate in this survey because we believe you may be involved, either now or in the past, in decisions about the prescription of antibiotics in this context. Please do not complete the survey if you are retired. We hope to recruit 300 participants to take part in our survey. We are interested in your views about what factors are important when deciding whether to continue or discontinue an antibiotic prescription.

**Do I have to take part?**

No, your participation in this survey is entirely voluntary. We will ask you to consent to participate on the next webpage by clicking on a tick box. If you choose not to click on this tick box you will not be able to take part in the study. If you begin to participate but then change your mind, you may withdraw without giving a reason and without penalty. You can withdraw at any time during the survey by simply closing your internet browser window. Your responses will not be submitted unless you select the tick box ‘submit survey’ at the very end.

**What does taking part involve?**

Taking part in this study simply involves completing our online survey. It should take around 20 minutes of your time. The survey technique that we have used is called a discrete choice experiment. In this survey you will be asked to consider a series of hypothetical scenarios. In each scenario you will be asked to make a decision about whether to continue or discontinue an antibiotic prescription, 72 hours after treatment has been initiated.

**What are the possible benefits of taking part?**

If you agree to take part in this survey, we hope that your views will inform future interventions aiming to reduce unnecessary antibiotic use in acute care. There is no remuneration for taking part in the study, but we hope that you will find some of the questions interesting and thought-provoking.

**Are there any risks in taking part?**

There will be no risk to you from taking part in this study.

**What will happen to my data?**

In this survey you will be asked to provide details such as your age, gender and primary clinical speciality, and to answer some questions about your personality. All of the information that you provide will be anonymous and no attempt will be made to identify you. Your information will only be used for research purposes, such as better understanding prescribing decisions in hospitals. However authorised representatives of the University of Oxford may be given access to data for monitoring and/or audit of the study.

Your responses will be stored in a password-protected file and may be used in academic publications but no individual-level information will be published. The University of Oxford is the data controller for the purposes of the Data Protection Act 1998. The data you provide will be archived indefinitely and may be shared with researchers at the University of Oxford or at other institutions for the purposes of research only. Please note that because the information you provide will be anonymised, it will not be possible for you to withdraw your data after you have completed the survey and submitted your responses.

**What will happen to the results of this study?**

We hope that the results of the study will be presented at academic conferences, published in a peer-reviewed journal and used to inform good clinical practice. It will not be possible to identify any participants in the study from any reports or publications.

**What if there is a problem?**

If a participant in University-sponsored research is ever considered to have suffered harm through their participation, the University has arrangements in place to provide for compensation. If you have a concern about any aspect of this study, please speak to Dr Laurence Roope (telephone 01865 617913), who will try to answer your query. Dr Roope will acknowledge your concern within 10 working days and give you an indication of how he intends to deal with it. If you remain unhappy or wish to make a formal complaint, please contact the University of Oxford’s Clinical Trials and Research Governance (CTRG) office on 01865 616480, or email at [ctrg@admin.ox.ac.uk](mailto:ctrg@admin.ox.ac.uk), who will also inform the chair of the Research Ethics Committee at the University of Oxford.

**Who is organising and funding the study?**

This research is sponsored by the University of Oxford, and funded by the National Institute for Health Research.

The project has been reviewed by, and received ethics clearance through, the University of Oxford’s Central University Research Ethics Committee [reference R56654/RE001].

Further information and contact details:

**Dr Laurence Roope**

Senior Researcher

Health Economics Research Centre

Nuffield Department of Population Health

University of Oxford

Old Road Campus

Headington

Oxford

OX3 7LF

Telephone: 01865 617913

Email: [laurence.roope@dph.ox.ac.uk](mailto:laurence.roope@dph.ox.ac.uk)

If you wish, you may proceed to give your consent to take part in the study on the next webpage.

**THANK YOU FOR YOUR HELP**

**CONSENT**

***A survey study to better understand healthcare workers’ views on starting and stopping antibiotics in the treatment of acute medical patients in secondary care***

If you wish to participate in this survey study, please indicate your consent by clicking on each of the four tick boxes below.

You will only take part in the study if you agree to click on each of these tick boxes.

I confirm that I have read the information about the above study on the previous webpage. I have had the opportunity to consider the information and ask questions, and have had any questions I have asked answered satisfactorily.

[Tick box]

I understand that my participation is voluntary and that I am free to withdraw at any time before submitting the survey.

[Tick box]

I understand that the survey results may be used to support other research in the future, and may be shared anonymously with other researchers.

[Tick box]

I agree to take part in the above study.

[Tick box]

**BACKGROUND**

This survey is about the treatment of acute medical patients in secondary care with antibiotics. Current guidelines recommend that antibiotic prescriptions are reviewed and revised within 72 hours of treatment initiation in hospitalised patients.

We are interested in your views about what factors are important when deciding whether to continue or discontinue an antibiotic prescription at this stage in the patient pathway. The survey technique that we are using is called a discrete choice experiment. In the survey you will be asked to consider a series of hypothetical scenarios. In each scenario you will be asked to make a decision about whether to continue or discontinue an antibiotic prescription, 72 hours after treatment has been initiated. The survey results will help us to better understand which factors are most important when healthcare professionals make antibiotic prescribing decisions in hospital, and will inform recommended clinical practice in the future.

**HOW TO COMPLETE THIS SURVEY**

Please consider the following hypothetical situation:

*You are reviewing the treatment of a patient who was admitted to hospital 72 hours ago. Antibiotic treatment was initiated in this patient within 2 hours of their admission. You now have to decide whether to continue or discontinue antibiotic treatment.*

In this survey you will be presented with several scenarios in which we will ask you to decide whether to continue or discontinue antibiotic treatment for this hypothetical patient.

In each scenario we will provide six pieces of information to help you to make this decision. This information relates to the condition of this patient, the likely consequences of continuing or discontinuing antibiotic treatment, and the potential pressures related to this clinical decision. The six pieces of information fall into the following categories, which will be described in more detail on the following webpages:

- Patient’s presenting symptoms
- Whether early discontinuation of antibiotic treatment within 72 hours of treatment initiation would be in conflict with local antibiotic guidelines
- Risk of significant harm arising from continued antibiotic treatment
- Risk of significant harm arising from discontinuing antibiotic treatment
- Premorbid condition of patient
- Level of external pressure to continue antibiotic treatment

There are three options in each of these categories, and these options will vary in each scenario. Detailed descriptions of each category of information and the possible options are provided on the following pages. Please read these descriptions carefully, then answer all of the questions that follow.

**THANK YOU FOR YOUR HELP**

**CATEGORY 1**

**Patient’s presenting symptoms**

The first category describes the symptoms that a patient presents with when they are admitted to hospital.

There are three possible options in this category:

*Symptoms indicating a urinary tract infection, with kidney pain*

*Fever, cough and possible pulmonary infiltrates on chest X-Ray*

*Off-legs and confused. Possible urinary tract infection. Possible lower respiratory tract infection. Might have experienced a fall*

**CATEGORY 2**

**Whether early discontinuation of antibiotic treatment within 72 hours of treatment initiation would be in conflict with local antibiotic guidelines**

The second category describes the extent to which there are conflicts between early discontinuation of antibiotic treatment and local antibiotic guidelines.

There are three possible options in this category:

*Early discontinuation would strongly conflict with local antibiotic guidelines*

*Early discontinuation would somewhat conflict with local antibiotic guidelines*

*Early discontinuation would not conflict with local antibiotic guidelines*

**CATEGORY 3**

**Risk of significant harm arising from continued antibiotic treatment**

The third category describes the likelihood that the patient will experience an adverse event from continued antibiotic treatment.

An adverse event might be a *C. difficile* infection, significant toxicity or drug-interactions, or a future antibiotic resistant infection.

There are three possible options in this category:

*Likely. In 30 cases out of every 100 like this, the patient will experience an adverse effect from continued antibiotic treatment.*

*
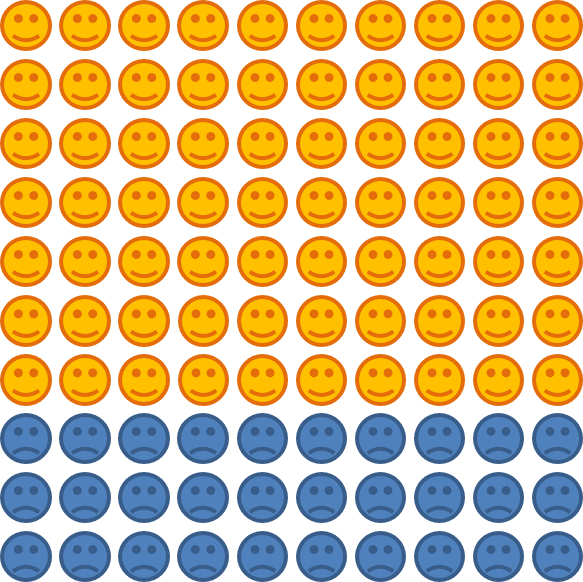
*

*Somewhat likely. In 10 cases out of every 100 like this, the patient will experience an adverse effect from continued antibiotic treatment.*

*
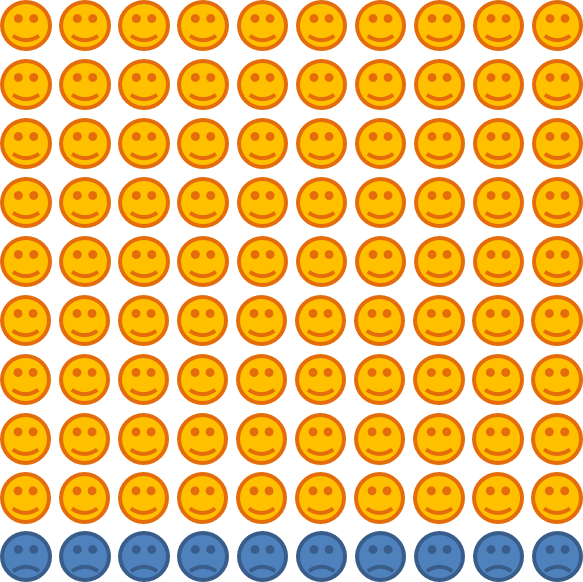
*

*Negligible. In 1 case out of every 100 like this, the patient will experience an adverse effect from continued antibiotic treatment.*

**
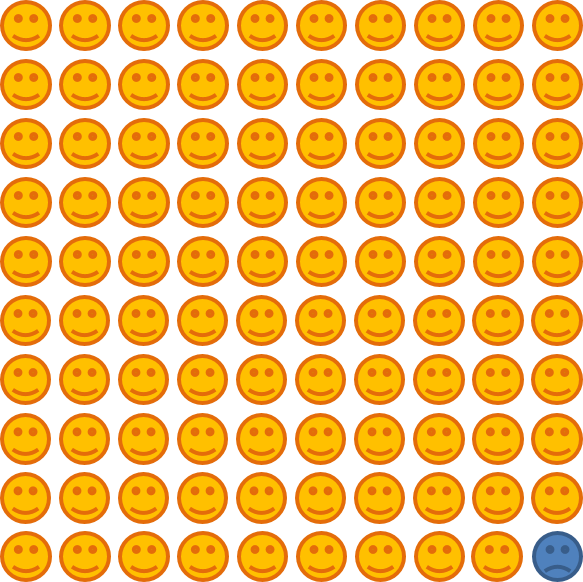
**

**CATEGORY 4**

**Risk of significant harm arising from discontinuing antibiotic treatment**

The fourth category describes the likelihood that the patient will experience significant harm from discontinuing antibiotic treatment.

This harm might be a relapse, recurrence of presenting symptoms or readmission.

There are three possible options in this category:

*Likely. In 30 cases out of every 100 like this, the patient will have a relapse, recurrence or readmission if antibiotic treatment is discontinued.*

*
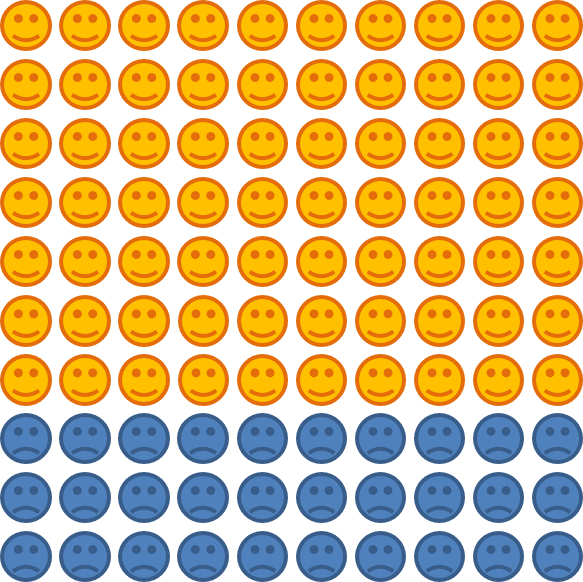
*

*Somewhat likely. In 10 cases out of every 100 like this, the patient will have a relapse, recurrence or readmission if antibiotic treatment is discontinued.*

*
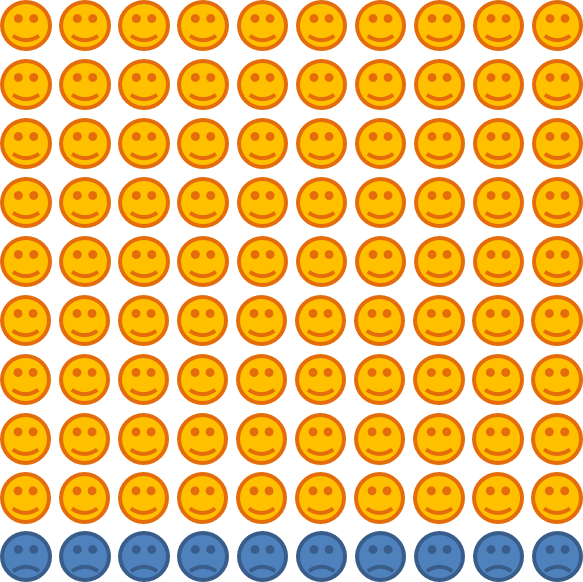
*

*Negligible. In 1 case out of every 100 like this, the patient will have a relapse, recurrence or readmission if antibiotic treatment is discontinued.*

**
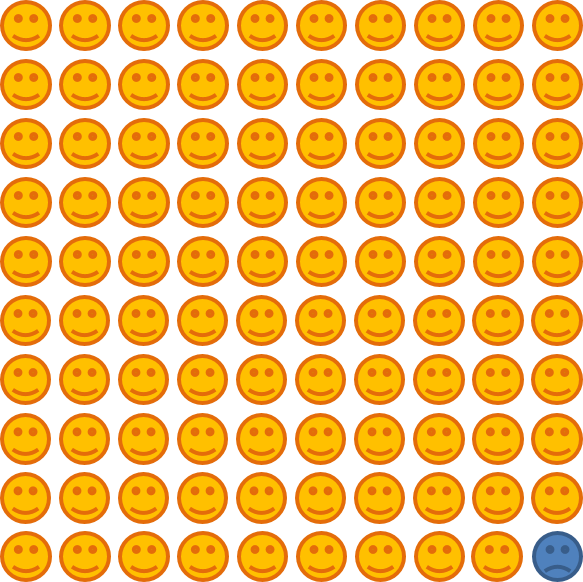
**

**CATEGORY 5**

**Premorbid condition of patient**

The fifth category describes the premorbid condition of the patient.

This category captures whether the patient was frail, or had comorbidities, prior to admission.

Possible comorbidities could include chronic lung disease, chronic cardiac disease, renal impairment, diabetes, hemiplegia, active malignancy or active rheumatological disease.

There are three possible options in this category:

*The patient has severe frailty and comorbidities.*

*The patient has moderate frailty and comorbidities.*

*The patient was previously fit and well.*

**CATEGORY 6**

**Level of external pressure to continue antibiotic treatment**

The sixth category describes the extent of external pressure to continue antibiotic treatment.

External pressure might come from bed managers, the patient’s family or other healthcare professionals.

There are three possible options in this category:

*There is no external pressure to continue antibiotic treatment.*

*There is some external pressure to continue antibiotic treatment.*

*There is heavy external pressure to continue antibiotic treatment.*

**SUMMARY OF CATEGORIES AND OPTIONS**

The following table summarises the six categories of information and the different options that may be presented to you in each scenario.

Please carefully review the information in this table before proceeding.

|  | **Category of information** | **Options** |
| --- | --- | --- |
| 1 | Patient’s presenting symptoms | Symptoms indicating a urinary tract infection, with kidney pain |
|  |  | Fever, cough and possible pulmonary infiltrates on chest X-Ray |
|  |  | Off-legs and confused. Possible urinary tract infection. Possible lower respiratory tract infection. Might have experienced a fall. |
| 2 | Whether early discontinuation of antibiotic treatment within 72 hours of treatment initiation would be in conflict with local antibiotic guidelines | Early discontinuation would strongly conflict with local antibiotic guidelines |
|  |  | Early discontinuation would somewhat conflict with local antibiotic guidelines |
|  |  | Early discontinuation would not conflict with local antibiotic guidelines |
| 3 | Risk of significant harm arising from continued antibiotic treatment | Likely. In 30 cases out of every 100 like this, the patient will experience an adverse effect from continued antibiotic treatment. |
|  |  | Somewhat likely. In 10 cases out of every 100 like this, the patient will experience an adverse effect from continued antibiotic treatment. |
|  |  | Negligible. In 1 case out of every 100 like this, the patient will experience an adverse effect from continued antibiotic treatment. |
| 4 | Risk of significant harm arising from discontinuing antibiotic treatment | Likely. In 30 cases out of every 100 like this, the patient will have a relapse, recurrence or readmission if antibiotic treatment is discontinued. |
|  |  | Somewhat likely. In 10 cases out of every 100 like this, the patient will have a relapse, recurrence or readmission if antibiotic treatment is discontinued. |
|  |  |  |
|  |  | Negligible. In 1 case out of every 100 like this, the patient will have a relapse, recurrence or readmission if antibiotic treatment is discontinued. |
| 5 | Premorbid condition of patient | The patient has severe frailty and comorbidities. |
|  |  | The patient has moderate frailty and comorbidities. |
|  |  | The patient was previously fit and well. |
| 6 | Level of external pressure to continue antibiotic treatment | There is no external pressure to continue antibiotic treatment |
|  |  | There is some external pressure to continue antibiotic treatment |
|  |  | There is heavy external pressure to continue antibiotic treatment |

**CATEGORY RANKING EXERCISE**

We would now like to know which of these categories of information are most important to you.

Please consider all six categories and then rank them below.

Double-click or drag-and-drop categories in the "Your choices" list to move them into the "Your ranking" list - your highest ranking category should be at the top of the list, moving through to your lowest ranking category at the bottom of the list.

If you would like to read the category descriptions again, please go back by clicking on the 'Previous' button.

[RANKING TABLE TO BE INSERTED]

**PRACTICE QUESTION**

Now we would like you to complete a practice choice question.

Consider the following hypothetical situation. This is the same situation that we presented to you earlier in the survey.

*You are reviewing the treatment of a patient who was admitted to hospital 72 hours ago. Antibiotic treatment was initiated in this patient within 2 hours of their admission. You now have to decide whether to continue or discontinue antibiotic treatment.*

The table below provides some information on the condition of this patient, the likely consequences of continuing and discontinuing antibiotic treatment, and the potential pressures related to this clinical decision.

Please review this information.

| **Category of information** | **Option** |
| --- | --- |
| Patient’s presenting symptoms | Off-legs and confused. Possible urinary tract infection. Possible lower respiratory tract infection. Might have experienced a fall |
| Whether early discontinuation of antibiotic treatment within 72 hours of treatment initiation would be in conflict with local antibiotic guidelines | Early discontinuation would not conflict with local antibiotic guidelines |
| Risk of significant harm arising from continued antibiotic treatment | Likely. In 30 cases out of every 100 like this, the patient will experience an adverse effect from continued antibiotic treatment.  *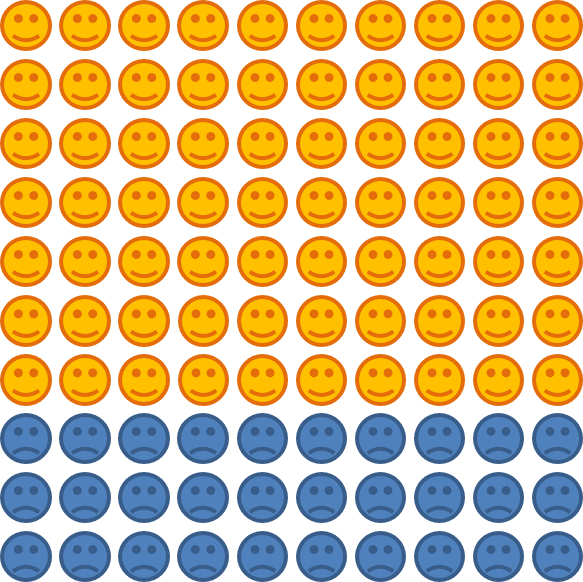* |
| Risk of significant harm arising from discontinuing antibiotic treatment | Negligible. In 1 case out of every 100 like this, the patient will have a relapse, recurrence or readmission if antibiotic treatment is discontinued.  **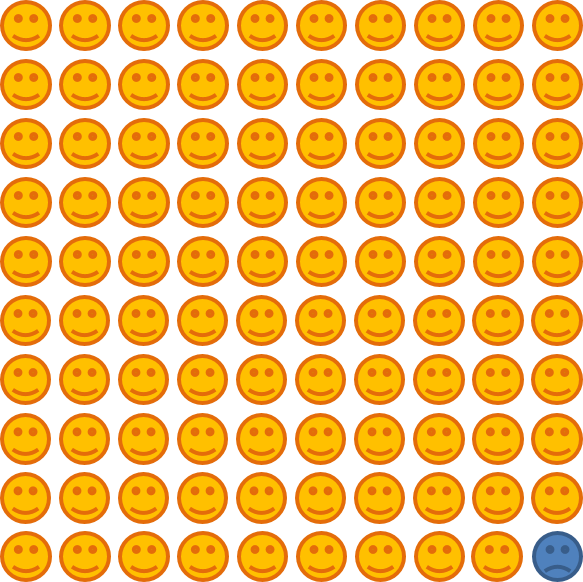** |
| Premorbid condition of patient | The patient was previously fit and well. |
| Level of external pressure to continue antibiotic treatment | There is no external pressure to continue antibiotic treatment. |

Given this information, would you choose to continue antibiotic treatment or discontinue antibiotic treatment in this patient? (tick one box only)

Continue antibiotic treatment 🞏

Discontinue antibiotic treatment 🞏

**MAIN QUESTIONS**

Thank you for completing the ranking exercise and practice question.

Now we would like you to complete the main part of the survey.

We are going to present you with 15 scenarios.

In each scenario you will be asked to consider the same situation that we presented to you in the practice question. However, the information that we provide to help you to decide whether to continue antibiotic treatment will vary from one scenario to the next.

Even if it is very difficult to make a decision, please indicate whether you would continue or discontinue antibiotic treatment in each scenario.

**QUESTION 1**

Consider the following hypothetical situation.

*You are reviewing the treatment of a patient who was admitted to hospital 72 hours ago. Antibiotic treatment was initiated in this patient within 2 hours of their admission. You now have to decide whether to continue or discontinue antibiotic treatment.*

The table below provides some information on the condition of this patient, the likely consequences of continuing and discontinuing antibiotic treatment, and the institutional and external pressures related to this clinical decision.

Please review this information.

| **Category of information** | **Option** |
| --- | --- |
| Patient’s presenting symptoms | Symptoms indicating a urinary tract infection, with kidney pain |
| Whether early discontinuation of antibiotic treatment within 72 hours of treatment initiation would be in conflict with local antibiotic guidelines | Early discontinuation would strongly conflict with local antibiotic guidelines |
| Risk of significant harm arising from continued antibiotic treatment | Likely. In 30 cases out of every 100 like this, the patient will experience an adverse effect from continued antibiotic treatment.  *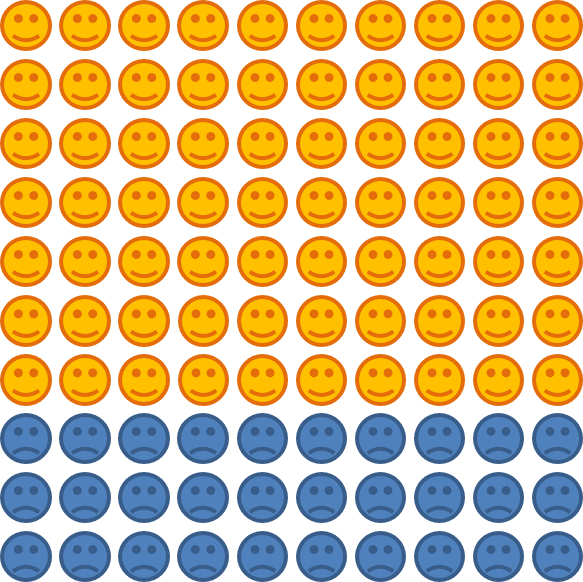* |
| Risk of significant harm arising from discontinuing antibiotic treatment | Somewhat likely. In 10 cases out of every 100 like this, the patient will have a relapse, recurrence or readmission if antibiotic treatment is discontinued.  *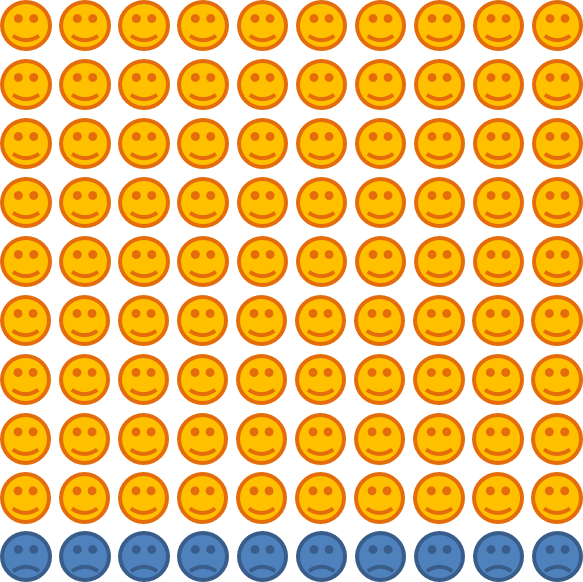* |
| Premorbid condition of patient | The patient has moderate frailty and comorbidities. |
| Level of external pressure to continue antibiotic treatment | There is heavy external pressure to continue antibiotic treatment |

Given this information, would you choose to continue antibiotic treatment or discontinue antibiotic treatment in this patient? (tick one box only)

Continue antibiotic treatment 🞏

Discontinue antibiotic treatment 🞏

**QUESTION 2**

Consider the following hypothetical situation.

*You are reviewing the treatment of a patient who was admitted to hospital 72 hours ago. Antibiotic treatment was initiated in this patient within 2 hours of their admission. You now have to decide whether to continue or discontinue antibiotic treatment.*

The table below provides some information on the condition of this patient, the likely consequences of continuing and discontinuing antibiotic treatment, and the institutional and external pressures related to this clinical decision.

Please review this information.

| **Category of information** | **Option** |
| --- | --- |
| Patient’s presenting symptoms | Fever, cough and possible pulmonary infiltrates on chest X-Ray |
| Whether early discontinuation of antibiotic treatment within 72 hours of treatment initiation would be in conflict with local antibiotic guidelines | Early discontinuation would somewhat conflict with local antibiotic guidelines |
| Risk of significant harm arising from continued antibiotic treatment | Likely. In 30 cases out of every 100 like this, the patient will experience an adverse effect from continued antibiotic treatment.  *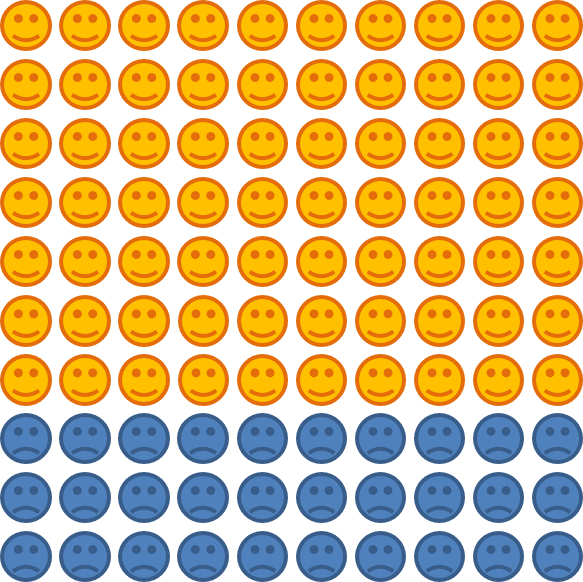* |
| Risk of significant harm arising from discontinuing antibiotic treatment | Likely. In 30 cases out of every 100 like this, the patient will have a relapse, recurrence or readmission if antibiotic treatment is discontinued.  *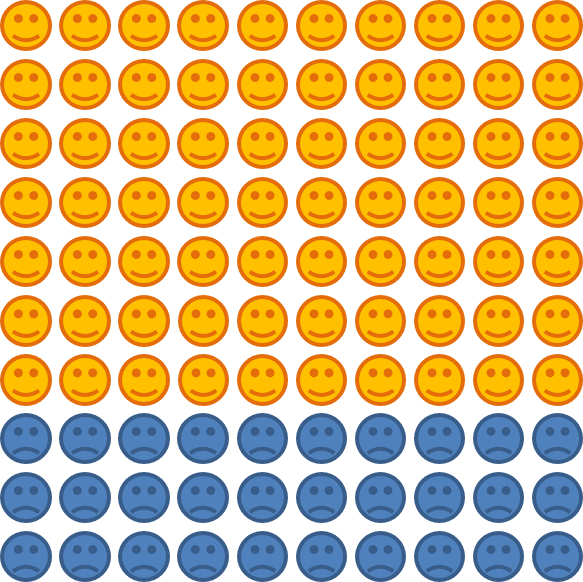* |
| Premorbid condition of patient | The patient was previously fit and well. |
| Level of external pressure to continue antibiotic treatment | There is heavy external pressure to continue antibiotic treatment |

Given this information, would you choose to continue antibiotic treatment or discontinue antibiotic treatment in this patient? (tick one box only)

Continue antibiotic treatment 🞏

Discontinue antibiotic treatment 🞏

**QUESTION 3**

Consider the following hypothetical situation.

*You are reviewing the treatment of a patient who was admitted to hospital 72 hours ago. Antibiotic treatment was initiated in this patient within 2 hours of their admission. You now have to decide whether to continue or discontinue antibiotic treatment.*

The table below provides some information on the condition of this patient, the likely consequences of continuing and discontinuing antibiotic treatment, and the institutional and external pressures related to this clinical decision.

Please review this information.

| **Category of information** | **Option** |
| --- | --- |
| Patient’s presenting symptoms | Fever, cough and possible pulmonary infiltrates on chest X-Ray |
| Whether early discontinuation of antibiotic treatment within 72 hours of treatment initiation would be in conflict with local antibiotic guidelines | Early discontinuation would not conflict with local antibiotic guidelines |
| Risk of significant harm arising from continued antibiotic treatment | Somewhat likely. In 10 cases out of every 100 like this, the patient will experience an adverse effect from continued antibiotic treatment.  *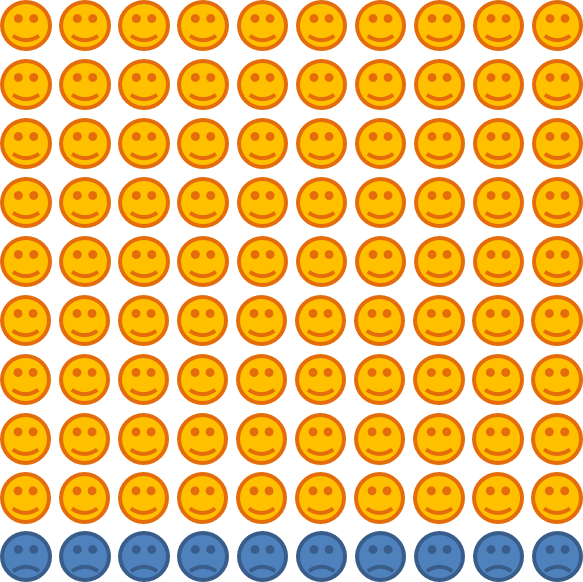* |
| Risk of significant harm arising from discontinuing antibiotic treatment | Somewhat likely. In 10 cases out of every 100 like this, the patient will have a relapse, recurrence or readmission if antibiotic treatment is discontinued.  *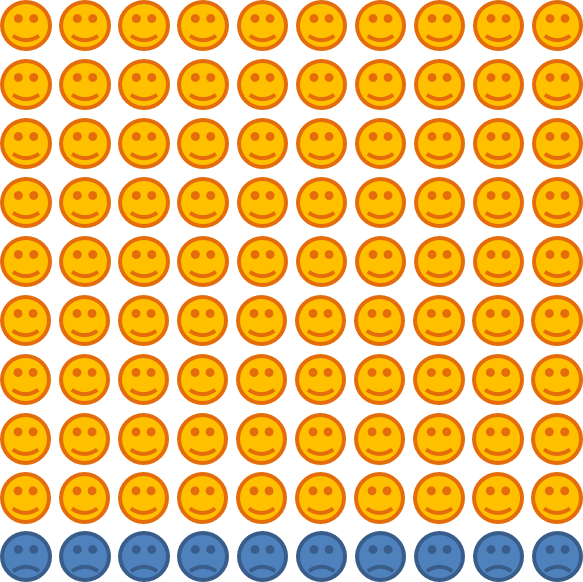* |
| Premorbid condition of patient | The patient has moderate frailty and comorbidities. |
|  |  |
| Level of external pressure to continue antibiotic treatment | There is heavy external pressure to continue antibiotic treatment |

Given this information, would you choose to continue antibiotic treatment or discontinue antibiotic treatment in this patient? (tick one box only)

Continue antibiotic treatment 🞏

Discontinue antibiotic treatment 🞏

**QUESTION 4**

Consider the following hypothetical situation.

*You are reviewing the treatment of a patient who was admitted to hospital 72 hours ago. Antibiotic treatment was initiated in this patient within 2 hours of their admission. You now have to decide whether to continue or discontinue antibiotic treatment.*

The table below provides some information on the condition of this patient, the likely consequences of continuing and discontinuing antibiotic treatment, and the institutional and external pressures related to this clinical decision.

Please review this information.

| **Category of information** | **Option** |
| --- | --- |
| Patient’s presenting symptoms | Off-legs and confused. Possible urinary tract infection. Possible lower respiratory tract infection. Might have experienced a fall. |
| Whether early discontinuation of antibiotic treatment within 72 hours of treatment initiation would be in conflict with local antibiotic guidelines | Early discontinuation would strongly conflict with local antibiotic guidelines |
| Risk of significant harm arising from continued antibiotic treatment | Negligible. In 1 case out of every 100 like this, the patient will experience an adverse effect from continued antibiotic treatment.  **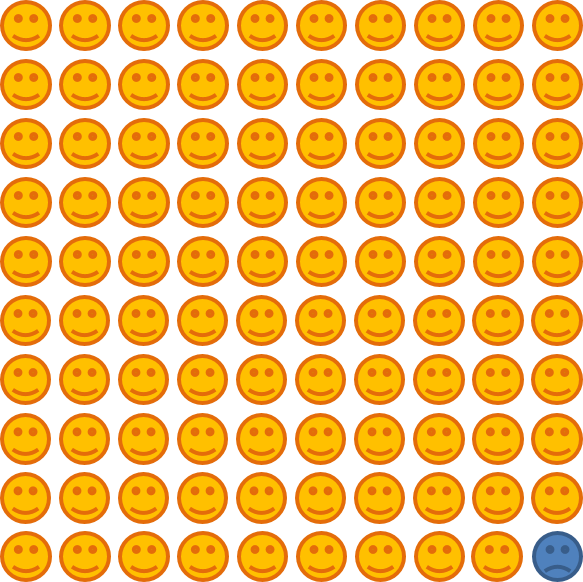** |
| Risk of significant harm arising from discontinuing antibiotic treatment | Somewhat likely. In 10 cases out of every 100 like this, the patient will have a relapse, recurrence or readmission if antibiotic treatment is discontinued.  *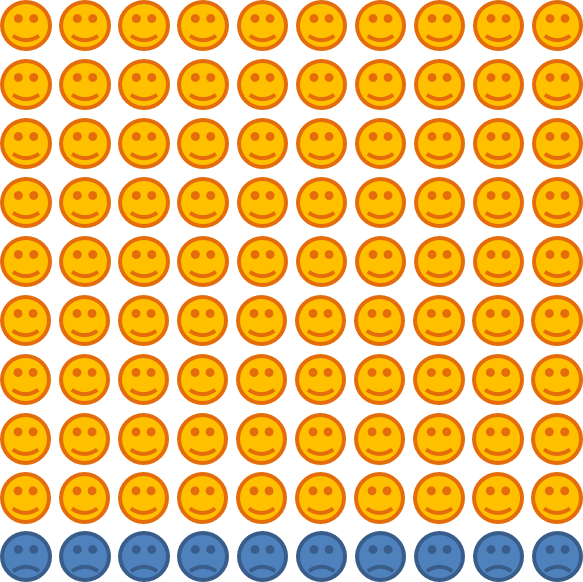* |
|  |  |
| Premorbid condition of patient | The patient has moderate frailty and comorbidities. |
| Level of external pressure to continue antibiotic treatment | There is no external pressure to continue antibiotic treatment |

Given this information, would you choose to continue antibiotic treatment or discontinue antibiotic treatment in this patient? (tick one box only)

Continue antibiotic treatment 🞏

Discontinue antibiotic treatment 🞏

**QUESTION 5**

Consider the following hypothetical situation.

*You are reviewing the treatment of a patient who was admitted to hospital 72 hours ago. Antibiotic treatment was initiated in this patient within 2 hours of their admission. You now have to decide whether to continue or discontinue antibiotic treatment.*

The table below provides some information on the condition of this patient, the likely consequences of continuing and discontinuing antibiotic treatment, and the institutional and external pressures related to this clinical decision.

Please review this information.

| **Category of information** | **Option** |
| --- | --- |
| Patient’s presenting symptoms | Fever, cough and possible pulmonary infiltrates on chest X-Ray |
| Whether early discontinuation of antibiotic treatment within 72 hours of treatment initiation would be in conflict with local antibiotic guidelines | Early discontinuation would not conflict with local antibiotic guidelines |
| Risk of significant harm arising from continued antibiotic treatment | Somewhat likely. In 10 cases out of every 100 like this, the patient will experience an adverse effect from continued antibiotic treatment.  *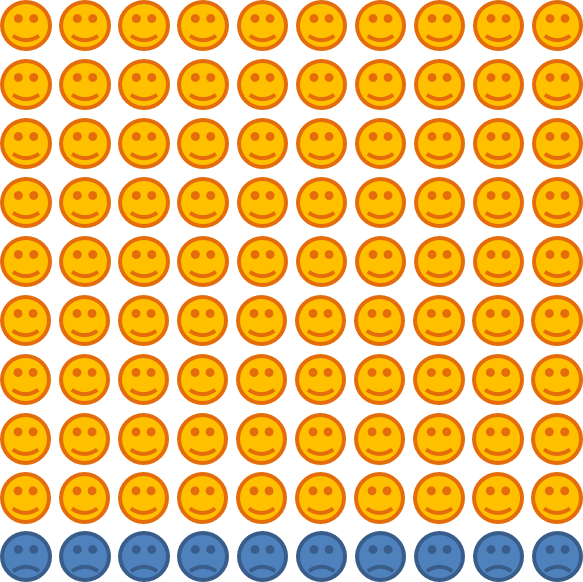* |
| Risk of significant harm arising from discontinuing antibiotic treatment | Negligible. In 1 case out of every 100 like this, the patient will have a relapse, recurrence or readmission if antibiotic treatment is discontinued.  **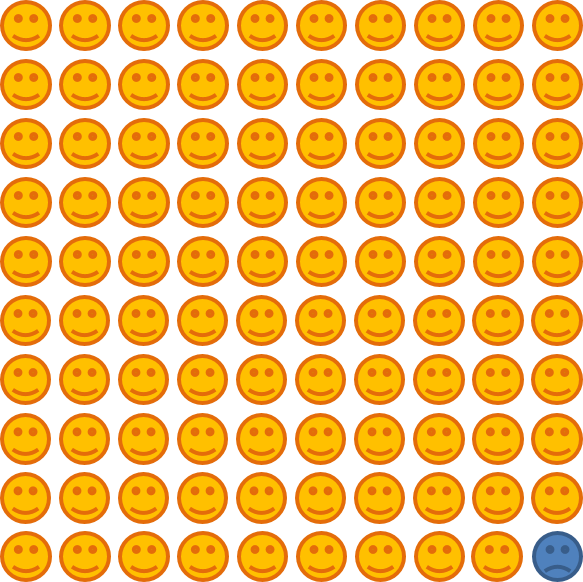** |
| Premorbid condition of patient | The patient has severe frailty and comorbidities. |
|  |  |
| Level of external pressure to continue antibiotic treatment | There is some external pressure to continue antibiotic treatment |

Given this information, would you choose to continue antibiotic treatment or discontinue antibiotic treatment in this patient? (tick one box only)

Continue antibiotic treatment 🞏

Discontinue antibiotic treatment 🞏

**QUESTION 6**

Consider the following hypothetical situation.

*You are reviewing the treatment of a patient who was admitted to hospital 72 hours ago. Antibiotic treatment was initiated in this patient within 2 hours of their admission. You now have to decide whether to continue or discontinue antibiotic treatment.*

The table below provides some information on the condition of this patient, the likely consequences of continuing and discontinuing antibiotic treatment, and the institutional and external pressures related to this clinical decision.

Please review this information.

| **Category of information** | **Option** |
| --- | --- |
| Patient’s presenting symptoms | Symptoms indicating a urinary tract infection, with kidney pain |
| Whether early discontinuation of antibiotic treatment within 72 hours of treatment initiation would be in conflict with local antibiotic guidelines | Early discontinuation would somewhat conflict with local antibiotic guidelines |
| Risk of significant harm arising from continued antibiotic treatment | Somewhat likely. In 10 cases out of every 100 like this, the patient will experience an adverse effect from continued antibiotic treatment.  *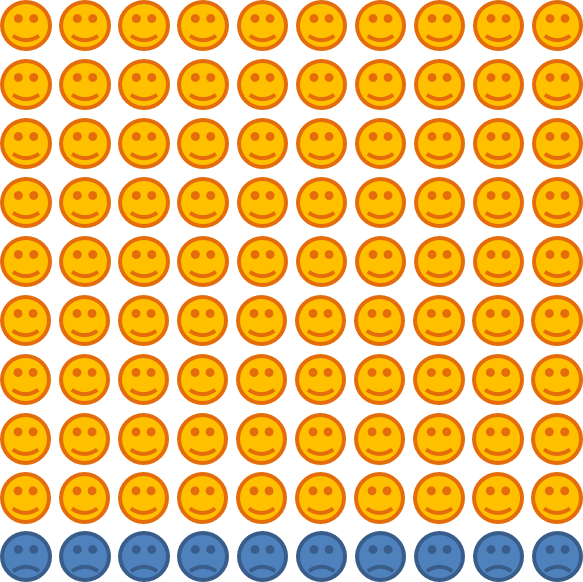* |
| Risk of significant harm arising from discontinuing antibiotic treatment | Negligible. In 1 case out of every 100 like this, the patient will have a relapse, recurrence or readmission if antibiotic treatment is discontinued.  **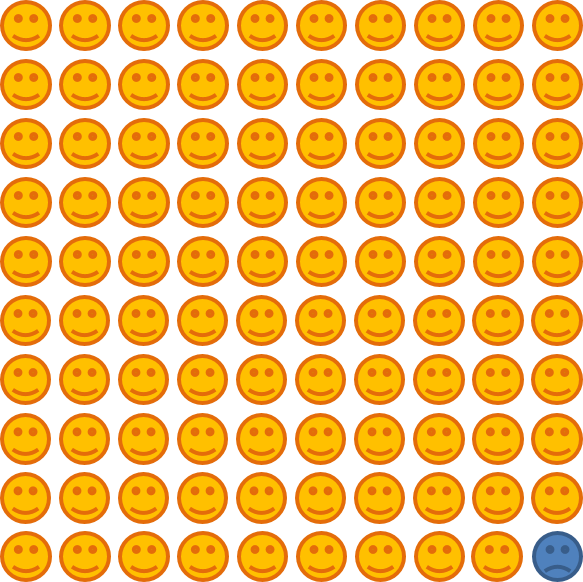** |
| Premorbid condition of patient | The patient was previously fit and well. |
| Level of external pressure to continue antibiotic treatment | There is no external pressure to continue antibiotic treatment |

Given this information, would you choose to continue antibiotic treatment or discontinue antibiotic treatment in this patient? (tick one box only)

Continue antibiotic treatment 🞏

Discontinue antibiotic treatment 🞏

**QUESTION 7**

Consider the following hypothetical situation.

*You are reviewing the treatment of a patient who was admitted to hospital 72 hours ago. Antibiotic treatment was initiated in this patient within 2 hours of their admission. You now have to decide whether to continue or discontinue antibiotic treatment.*

The table below provides some information on the condition of this patient, the likely consequences of continuing and discontinuing antibiotic treatment, and the institutional and external pressures related to this clinical decision.

Please review this information.

| **Category of information** | **Option** |
| --- | --- |
| Patient’s presenting symptoms | Fever, cough and possible pulmonary infiltrates on chest X-Ray |
| Whether early discontinuation of antibiotic treatment within 72 hours of treatment initiation would be in conflict with local antibiotic guidelines | Early discontinuation would somewhat conflict with local antibiotic guidelines |
| Risk of significant harm arising from continued antibiotic treatment | Negligible. In 1 case out of every 100 like this, the patient will experience an adverse effect from continued antibiotic treatment.  **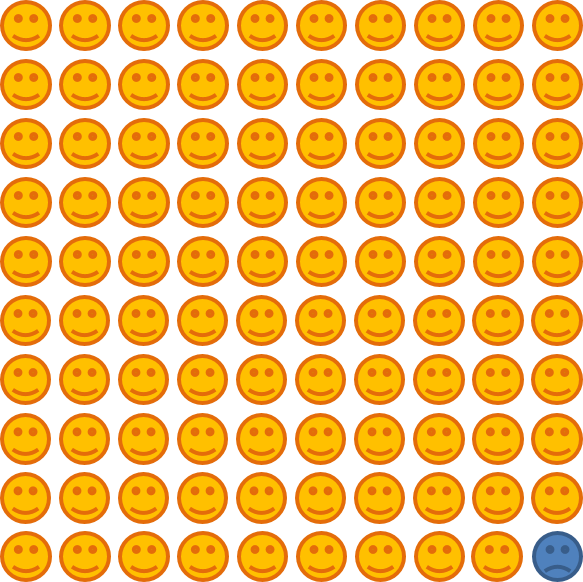** |
| Risk of significant harm arising from discontinuing antibiotic treatment | Negligible. In 1 case out of every 100 like this, the patient will have a relapse, recurrence or readmission if antibiotic treatment is discontinued.  **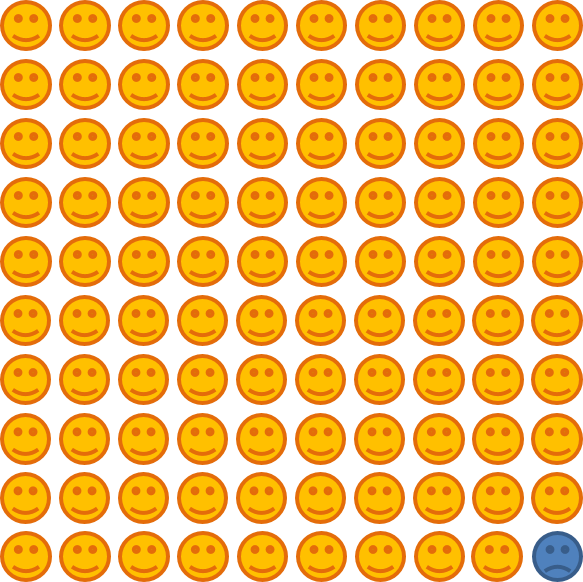** |
| Premorbid condition of patient | The patient has moderate frailty and comorbidities. |
| Level of external pressure to continue antibiotic treatment | There is no external pressure to continue antibiotic treatment |

Given this information, would you choose to continue antibiotic treatment or discontinue antibiotic treatment in this patient? (tick one box only)

Continue antibiotic treatment 🞏

Discontinue antibiotic treatment 🞏

**QUESTION 8**

Consider the following hypothetical situation.

*You are reviewing the treatment of a patient who was admitted to hospital 72 hours ago. Antibiotic treatment was initiated in this patient within 2 hours of their admission. You now have to decide whether to continue or discontinue antibiotic treatment.*

The table below provides some information on the condition of this patient, the likely consequences of continuing and discontinuing antibiotic treatment, and the institutional and external pressures related to this clinical decision.

Please review this information.

| **Category of information** | **Option** |
| --- | --- |
| Patient’s presenting symptoms | Off-legs and confused. Possible urinary tract infection. Possible lower respiratory tract infection. Might have experienced a fall. |
| Whether early discontinuation of antibiotic treatment within 72 hours of treatment initiation would be in conflict with local antibiotic guidelines | Early discontinuation would not conflict with local antibiotic guidelines |
| Risk of significant harm arising from continued antibiotic treatment | Likely. In 30 cases out of every 100 like this, the patient will experience an adverse effect from continued antibiotic treatment.  *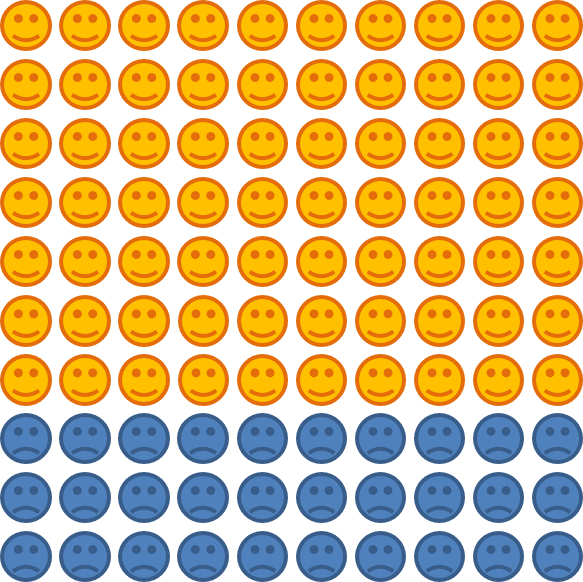* |
| Risk of significant harm arising from discontinuing antibiotic treatment | Likely. In 30 cases out of every 100 like this, the patient will have a relapse, recurrence or readmission if antibiotic treatment is discontinued.  *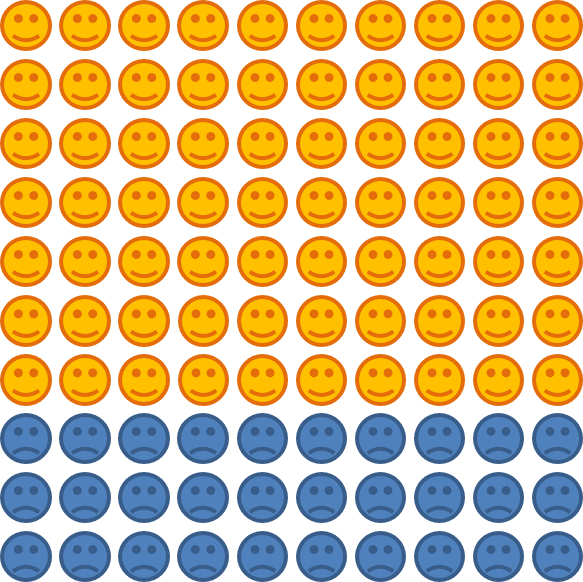* |
| Premorbid condition of patient | The patient has severe frailty and comorbidities. |
| Level of external pressure to continue antibiotic treatment | There is no external pressure to continue antibiotic treatment |

Given this information, would you choose to continue antibiotic treatment or discontinue antibiotic treatment in this patient? (tick one box only)

Continue antibiotic treatment 🞏

Discontinue antibiotic treatment 🞏

**QUESTION 9**

Consider the following hypothetical situation.

*You are reviewing the treatment of a patient who was admitted to hospital 72 hours ago. Antibiotic treatment was initiated in this patient within 2 hours of their admission. You now have to decide whether to continue or discontinue antibiotic treatment.*

The table below provides some information on the condition of this patient, the likely consequences of continuing and discontinuing antibiotic treatment, and the institutional and external pressures related to this clinical decision.

Please review this information.

| **Category of information** | **Option** |
| --- | --- |
| Patient’s presenting symptoms | Off-legs and confused. Possible urinary tract infection. Possible lower respiratory tract infection. Might have experienced a fall. |
| Whether early discontinuation of antibiotic treatment within 72 hours of treatment initiation would be in conflict with local antibiotic guidelines | Early discontinuation would somewhat conflict with local antibiotic guidelines |
| Risk of significant harm arising from continued antibiotic treatment | Negligible. In 1 case out of every 100 like this, the patient will experience an adverse effect from continued antibiotic treatment.  **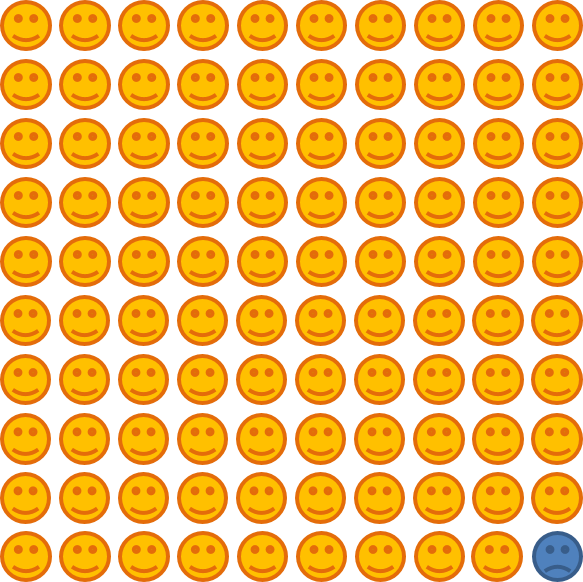** |
| Risk of significant harm arising from discontinuing antibiotic treatment | Negligible. In 1 case out of every 100 like this, the patient will have a relapse, recurrence or readmission if antibiotic treatment is discontinued.  **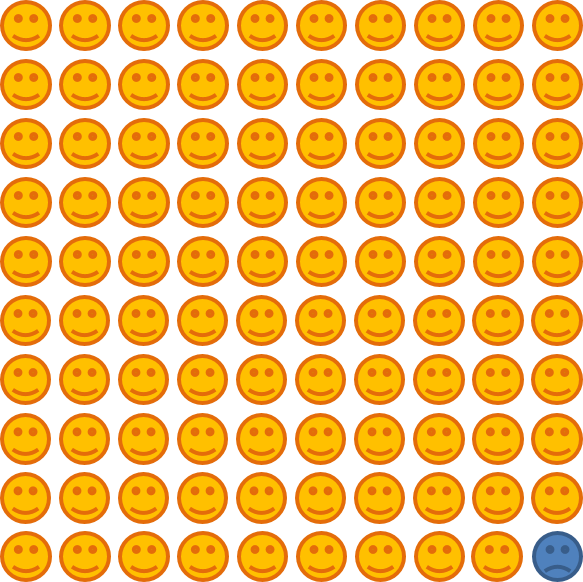** |
| Premorbid condition of patient | The patient has severe frailty and comorbidities. |
| Level of external pressure to continue antibiotic treatment | There is heavy external pressure to continue antibiotic treatment |

Given this information, would you choose to continue antibiotic treatment or discontinue antibiotic treatment in this patient? (tick one box only)

Continue antibiotic treatment 🞏

Discontinue antibiotic treatment 🞏

**QUESTION 10**

Consider the following hypothetical situation.

*You are reviewing the treatment of a patient who was admitted to hospital 72 hours ago. Antibiotic treatment was initiated in this patient within 2 hours of their admission. You now have to decide whether to continue or discontinue antibiotic treatment.*

The table below provides some information on the condition of this patient, the likely consequences of continuing and discontinuing antibiotic treatment, and the institutional and external pressures related to this clinical decision.

Please review this information.

| **Category of information** | **Option** |
| --- | --- |
| Patient’s presenting symptoms | Symptoms indicating a urinary tract infection, with kidney pain |
| Whether early discontinuation of antibiotic treatment within 72 hours of treatment initiation would be in conflict with local antibiotic guidelines | Early discontinuation would somewhat conflict with local antibiotic guidelines |
| Risk of significant harm arising from continued antibiotic treatment | Somewhat likely. In 10 cases out of every 100 like this, the patient will experience an adverse effect from continued antibiotic treatment.  *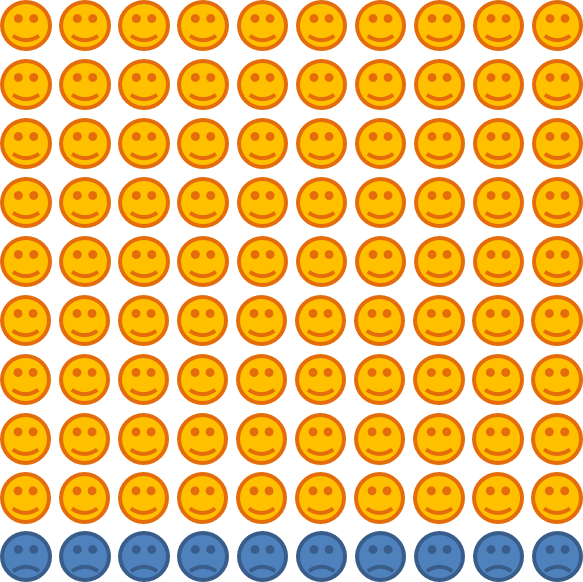* |
| Risk of significant harm arising from discontinuing antibiotic treatment | Somewhat likely. In 10 cases out of every 100 like this, the patient will have a relapse, recurrence or readmission if antibiotic treatment is discontinued.  *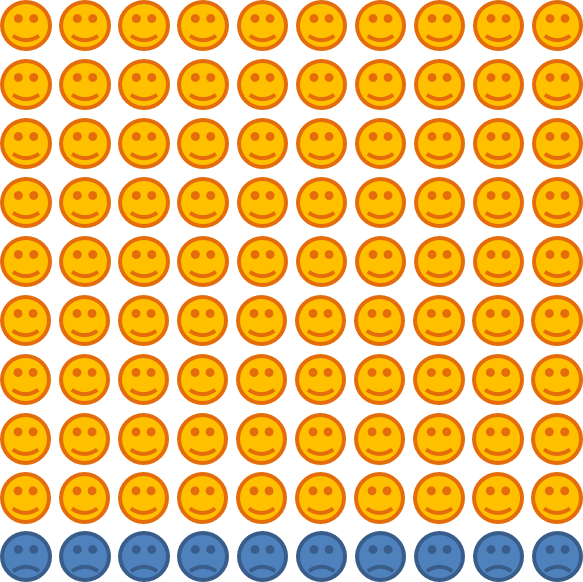* |
| Premorbid condition of patient | The patient has severe frailty and comorbidities. |
| Level of external pressure to continue antibiotic treatment | There is some external pressure to continue antibiotic treatment |

Given this information, would you choose to continue antibiotic treatment or discontinue antibiotic treatment in this patient? (tick one box only)

Continue antibiotic treatment 🞏

Discontinue antibiotic treatment 🞏

**QUESTION 11**

Consider the following hypothetical situation.

*You are reviewing the treatment of a patient who was admitted to hospital 72 hours ago. Antibiotic treatment was initiated in this patient within 2 hours of their admission. You now have to decide whether to continue or discontinue antibiotic treatment.*

The table below provides some information on the condition of this patient, the likely consequences of continuing and discontinuing antibiotic treatment, and the institutional and external pressures related to this clinical decision.

Please review this information.

| **Category of information** | **Option** |
| --- | --- |
| Patient’s presenting symptoms | Fever, cough and possible pulmonary infiltrates on chest X-Ray |
| Whether early discontinuation of antibiotic treatment within 72 hours of treatment initiation would be in conflict with local antibiotic guidelines | Early discontinuation would strongly conflict with local antibiotic guidelines |
| Risk of significant harm arising from continued antibiotic treatment | Likely. In 30 cases out of every 100 like this, the patient will experience an adverse effect from continued antibiotic treatment.  *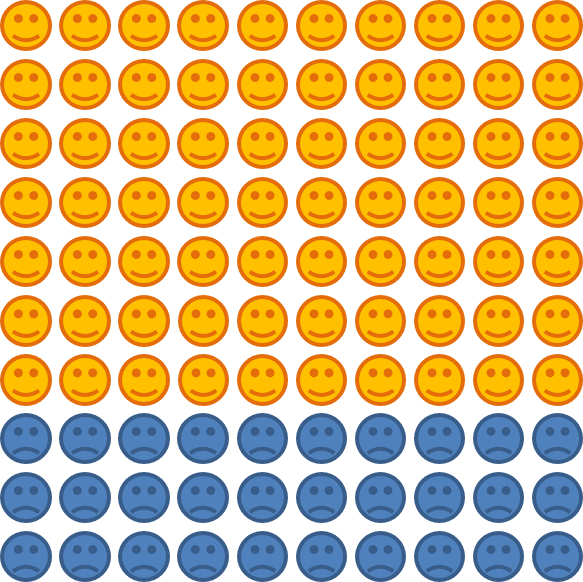* |
| Risk of significant harm arising from discontinuing antibiotic treatment | Likely. In 30 cases out of every 100 like this, the patient will have a relapse, recurrence or readmission if antibiotic treatment is discontinued.  *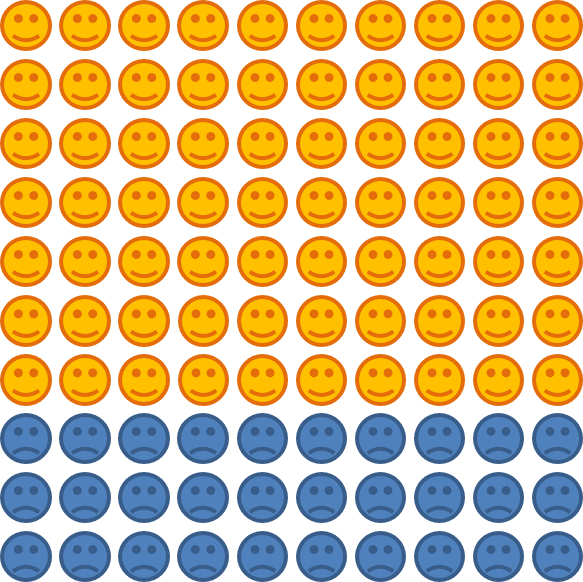* |
| Premorbid condition of patient | The patient has severe frailty and comorbidities. |
| Level of external pressure to continue antibiotic treatment | There is no external pressure to continue antibiotic treatment |

Given this information, would you choose to continue antibiotic treatment or discontinue antibiotic treatment in this patient? (tick one box only)

Continue antibiotic treatment 🞏

Discontinue antibiotic treatment 🞏

**QUESTION 12**

Consider the following hypothetical situation.

*You are reviewing the treatment of a patient who was admitted to hospital 72 hours ago. Antibiotic treatment was initiated in this patient within 2 hours of their admission. You now have to decide whether to continue or discontinue antibiotic treatment.*

The table below provides some information on the condition of this patient, the likely consequences of continuing and discontinuing antibiotic treatment, and the institutional and external pressures related to this clinical decision.

Please review this information.

| **Category of information** | **Option** |
| --- | --- |
| Patient’s presenting symptoms | Off-legs and confused. Possible urinary tract infection. Possible lower respiratory tract infection. Might have experienced a fall. |
| Whether early discontinuation of antibiotic treatment within 72 hours of treatment initiation would be in conflict with local antibiotic guidelines | Early discontinuation would not conflict with local antibiotic guidelines |
| Risk of significant harm arising from continued antibiotic treatment | Negligible. In 1 case out of every 100 like this, the patient will experience an adverse effect from continued antibiotic treatment.  **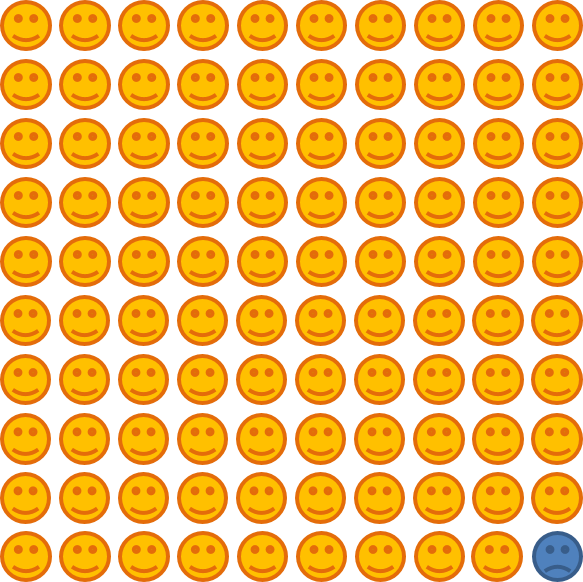** |
| Risk of significant harm arising from discontinuing antibiotic treatment | Likely. In 30 cases out of every 100 like this, the patient will have a relapse, recurrence or readmission if antibiotic treatment is discontinued.  *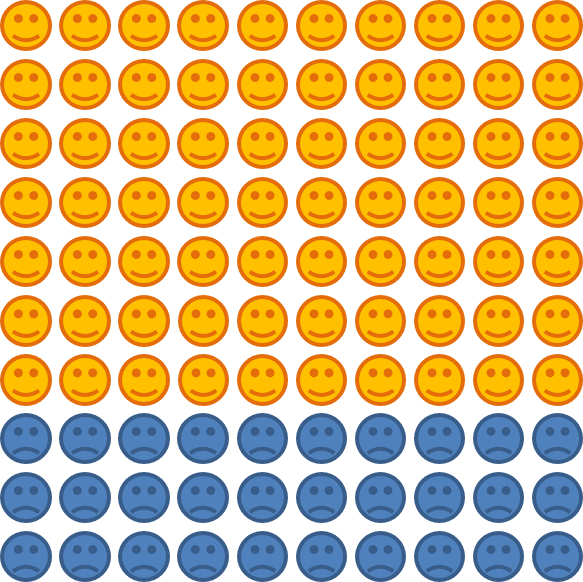* |
| Premorbid condition of patient | The patient was previously fit and well. |
| Level of external pressure to continue antibiotic treatment | There is some external pressure to continue antibiotic treatment |

Given this information, would you choose to continue antibiotic treatment or discontinue antibiotic treatment in this patient? (tick one box only)

Continue antibiotic treatment 🞏

Discontinue antibiotic treatment 🞏

**QUESTION 13**

Consider the following hypothetical situation.

*You are reviewing the treatment of a patient who was admitted to hospital 72 hours ago. Antibiotic treatment was initiated in this patient within 2 hours of their admission. You now have to decide whether to continue or discontinue antibiotic treatment.*

The table below provides some information on the condition of this patient, the likely consequences of continuing and discontinuing antibiotic treatment, and the institutional and external pressures related to this clinical decision.

Please review this information.

| **Category of information** | **Option** |
| --- | --- |
| Patient’s presenting symptoms | Off-legs and confused. Possible urinary tract infection. Possible lower respiratory tract infection. Might have experienced a fall. |
| Whether early discontinuation of antibiotic treatment within 72 hours of treatment initiation would be in conflict with local antibiotic guidelines | Early discontinuation would somewhat conflict with local antibiotic guidelines |
| Risk of significant harm arising from continued antibiotic treatment | Likely. In 30 cases out of every 100 like this, the patient will experience an adverse effect from continued antibiotic treatment.  *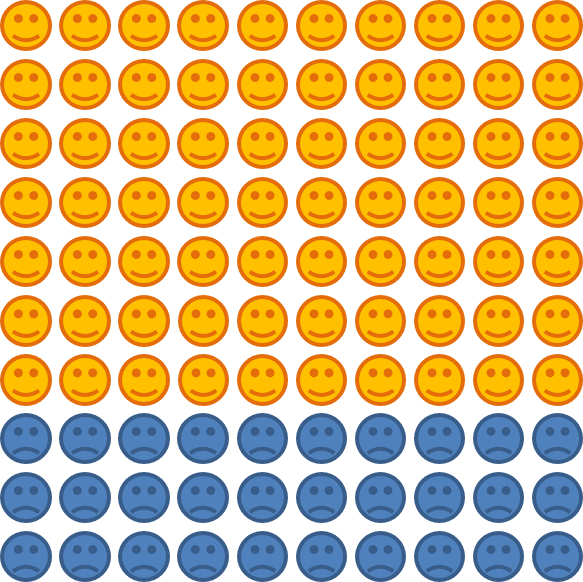* |
| Risk of significant harm arising from discontinuing antibiotic treatment | Likely. In 30 cases out of every 100 like this, the patient will have a relapse, recurrence or readmission if antibiotic treatment is discontinued.  *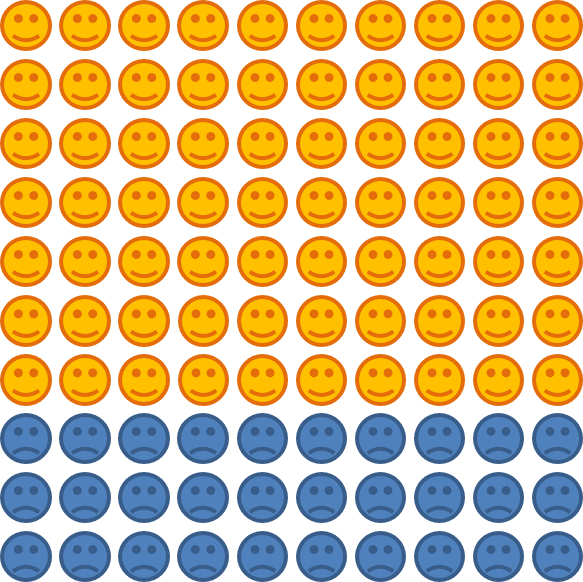* |
| Premorbid condition of patient | The patient has moderate frailty and comorbidities. |
|  |  |
| Level of external pressure to continue antibiotic treatment | There is some external pressure to continue antibiotic treatment |

Given this information, would you choose to continue antibiotic treatment or discontinue antibiotic treatment in this patient? (tick one box only)

Continue antibiotic treatment 🞏

Discontinue antibiotic treatment 🞏

**QUESTION 14**

Consider the following hypothetical situation.

*You are reviewing the treatment of a patient who was admitted to hospital 72 hours ago. Antibiotic treatment was initiated in this patient within 2 hours of their admission. You now have to decide whether to continue or discontinue antibiotic treatment.*

The table below provides some information on the condition of this patient, the likely consequences of continuing and discontinuing antibiotic treatment, and the institutional and external pressures related to this clinical decision.

Please review this information.

| **Category of information** | **Option** |
| --- | --- |
| Patient’s presenting symptoms | Symptoms indicating a urinary tract infection, with kidney pain |
| Whether early discontinuation of antibiotic treatment within 72 hours of treatment initiation would be in conflict with local antibiotic guidelines | Early discontinuation would strongly conflict with local antibiotic guidelines |
| Risk of significant harm arising from continued antibiotic treatment | Negligible. In 1 case out of every 100 like this, the patient will experience an adverse effect from continued antibiotic treatment.  **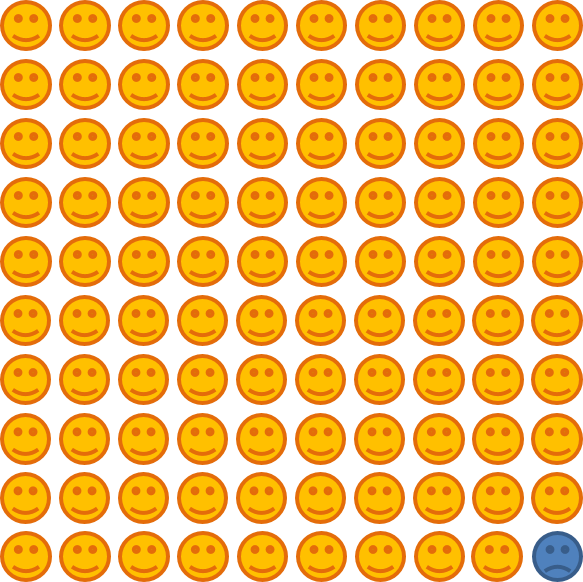** |
| Risk of significant harm arising from discontinuing antibiotic treatment | Somewhat likely. In 10 cases out of every 100 like this, the patient will have a relapse, recurrence or readmission if antibiotic treatment is discontinued.  *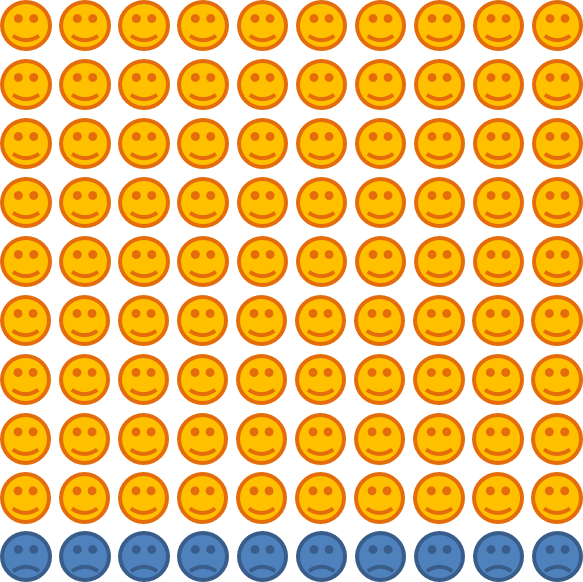* |
| Premorbid condition of patient | The patient was previously fit and well. |
| Level of external pressure to continue antibiotic treatment | There is heavy external pressure to continue antibiotic treatment |

Given this information, would you choose to continue antibiotic treatment or discontinue antibiotic treatment in this patient? (tick one box only)

Continue antibiotic treatment 🞏

Discontinue antibiotic treatment 🞏

**QUESTION 15**

Consider the following hypothetical situation.

*You are reviewing the treatment of a patient who was admitted to hospital 72 hours ago. Antibiotic treatment was initiated in this patient within 2 hours of their admission. You now have to decide whether to continue or discontinue antibiotic treatment.*

The table below provides some information on the condition of this patient, the likely consequences of continuing and discontinuing antibiotic treatment, and the institutional and external pressures related to this clinical decision.

Please review this information.

| **Category of information** | **Option** |
| --- | --- |
| Patient’s presenting symptoms | Fever, cough and possible pulmonary infiltrates on chest X-Ray |
| Whether early discontinuation of antibiotic treatment within 72 hours of treatment initiation would be in conflict with local antibiotic guidelines | Early discontinuation would strongly conflict with local antibiotic guidelines |
| Risk of significant harm arising from continued antibiotic treatment | Somewhat likely. In 10 cases out of every 100 like this, the patient will experience an adverse effect from continued antibiotic treatment.  *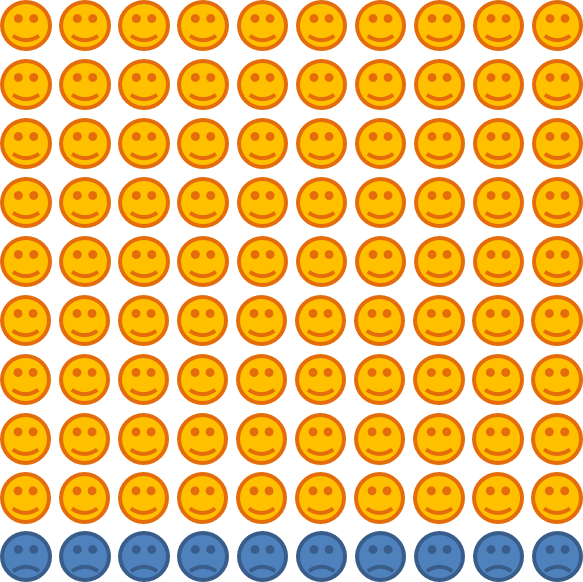* |
| Risk of significant harm arising from discontinuing antibiotic treatment | Negligible. In 1 case out of every 100 like this, the patient will have a relapse, recurrence or readmission if antibiotic treatment is discontinued.  **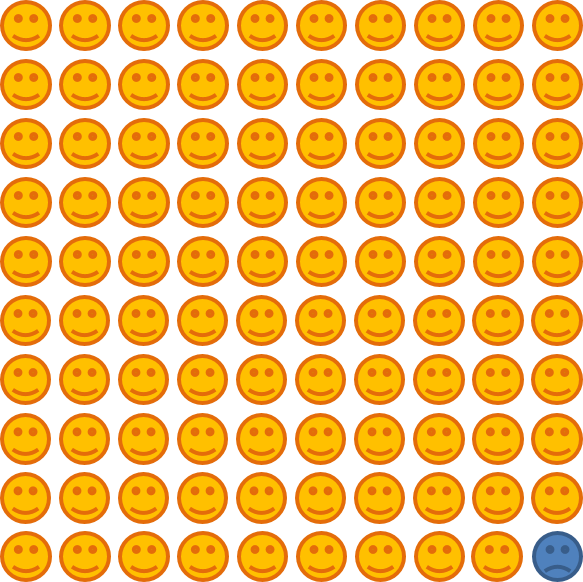** |
| Premorbid condition of patient | The patient was previously fit and well. |
| Level of external pressure to continue antibiotic treatment | There is some external pressure to continue antibiotic treatment |

Given this information, would you choose to continue antibiotic treatment or discontinue antibiotic treatment in this patient? (tick one box only)

Continue antibiotic treatment 🞏

Discontinue antibiotic treatment 🞏

**CATEGORY RANKING EXERCISE – PART TWO**

Thank you for completing the main part of the survey.

Now we would like you to complete the category ranking exercise again to see if your opinions have changed after answering these questions.

Please consider how important the six categories of information are to you and then rank them below.

Double-click or drag-and-drop categories in the "Your choices" list to move them into the "Your ranking" list - your highest ranking category should be at the top of the list, moving through to your lowest ranking category at the bottom of the list.

[RANKING TABLE TO BE INSERTED]

**RESPONDENT CHARACTERISTICS**

Now we would like to ask some questions about you.

All of the information that you provide will help us in our analysis, and all of your details will remain confidential.

If you do not wish to answer some of these questions you do not have to – you can just skip the question.

1. Approximately how many times would you normally make an antibiotic review decision in hospital in a typical month?
2. Thinking about the antibiotic review decisions that you have made in the past year, approximately what percentage of your decisions fall into each of the following categories.

Stop antibiotic treatment

Switch from IV to oral antibiotics

Change antibiotic to a drug with a narrower spectrum

Change antibiotic to a drug with a broader spectrum

Continue with the current antibiotic drug and provide a review date

Continue with the current antibiotic drug without a review date (if your Electronic Patient Record system doesn't allow this, please put 0%)

Another decision (please describe)

1. How old are you?

Under 25 years old

25-34 years old

35-44 years old

45-54 years old

55-64 years old

Over 64 years old

1. What is your gender?

Male

Female

Other

Prefer not to say

1. How many beds are there in your NHS Trust?

Fewer than 500 beds

Between 500 and 1,000 beds

More than 1,000 beds

1. Which of the following options best describes your main occupation?

Consultant

Staff Grade or Associate Specialist

Foundation Year One Doctor

Foundation Year Two Doctor

Core Medical Trainee

General Practice Specialty Registrar or Specialty Trainee/Registrar

Non-medical Prescriber (e.g. Nurse or Pharmacist)

Other (please describe)

1. What is your primary clinical specialty?

Acute or General Medicine

Microbiology or Infectious Diseases

Non-infection related medical specialty

No primary clinical specialty

Other (please describe)

1. We would like to end this survey by asking about how you see yourself. There are 10 statements below. Please indicate how well each of these statements describes your personality. (This question is not mandatory)

“I see myself as someone who is reserved.”

“I see myself as someone who is generally trusting.”

“I see myself as someone who tends to be lazy.”

“I see myself as someone who is relaxed, handles stress well.”

“I see myself as someone who has few artistic interests.”

“I see myself as someone who is outgoing, sociable.”

“I see myself as someone who tends to find fault with others.”

“I see myself as someone who does a thorough job.”

“I see myself as someone who gets nervous easily.”

“I see myself as someone who has an active imagination.”

Five options will be provided for each statement:

Agree strongly | Agree a little | Neither agree nor disagree | Disagree a little | Disagree strongly

1. Finally, are you generally a person who is fully willing to take risks or do you try to avoid taking risks? Please choose a box on the scale below, where 0 means “risk averse” and 10 means “fully prepared to take risks”.

Not at all prepared to take risks

Fully prepared to take risks


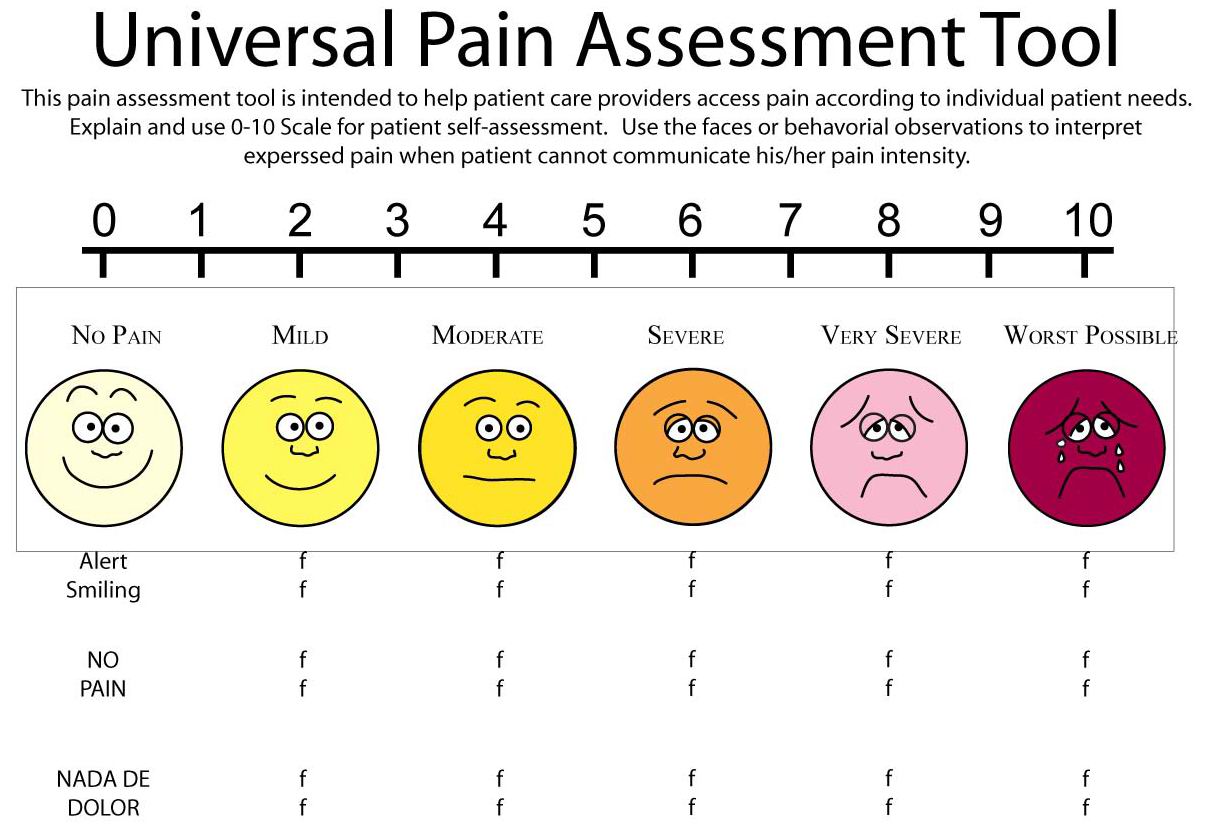


1. How easy or difficult did you find the questions in this survey?

[Likert scale 1-7, 1 = very easy, 7 = very difficult]

1. Are there any further comments that you would like to make regarding this survey?

You have reached the end of the survey. Thank you for taking the time to participate. If you have any questions about this survey, please contact Dr Laurence Roope ([laurence.roope@dph.ox.ac.uk](mailto:laurence.roope@dph.ox.ac.uk)).
