# Supplementary material for: Why do hospital prescribers continue antibiotics when it is safe to stop? Results of a choice experiment survey
Source: BMC Med. 2020 Jul 30;18:196. doi: 10.1186/s12916-020-01660-4 (PMC7391515; doi:10.1186/s12916-020-01660-4)

# Additional file 4: Recruitment email text

**Subject line:** Request to contribute to a research study about antibiotic prescribing

Dear Member [*‘Dear Trainee’ for those contacted via Deaneries*],

I am writing to ask if you would be willing to take part in a brief survey which is part of a major NIHR-funded research programme in the field of antibiotic prescribing. I appreciate how busy you will be but you will also know that this is a very important challenge for the NHS at the moment and an area where we need to change practice to protect patients.

The Antibiotic Review Kit (ARK) research programme is about supporting prescribers to make decisions to safely stop antibiotics sooner, in hospital medical practice. You can learn more about the study here if you are interested: [www.arkstudy.ox.ac.uk](http://www.arkstudy.ox.ac.uk).

This survey aims to help us understand how healthcare workers reviewing antibiotic prescriptions decide whether treatment should be continued or stopped.

You have been invited to participate in this survey as we believe you may be involved, either now or in the past, in decisions about prescribing antibiotics for acute or general medical patients in secondary care. This is in light of your [*one of the following to be inserted here as appropriate*]

membership of the Society for Acute Medicine (SAM).

engagement in postgraduate training in acute/general medicine.

We are interested in your views about what factors are important when deciding whether to continue or discontinue an antibiotic prescription. Our findings will help to develop interventions to reduce unnecessary antibiotic use. Please complete the survey only if you are, or have been, involved in prescribing antibiotics for acute or general medical patients in secondary care in the United Kingdom. [*previous sentence to be included only in invitation to members of SAM*]

This is an online survey. The survey technique it uses is called a **discrete choice experiment**. In the survey you will be asked to consider a series of hypothetical scenarios. In each, you will be asked to make a decision about whether to continue or discontinue an antibiotic prescription, 72 hours after treatment has been initiated. If you would like to participate in this survey, please click on the following link for further information:

<https://survey.msd.ox.ac.uk/index.php/858955?lang=en>

The survey should take around 20 minutes to complete.

**The deadline for completing this survey is [***date 2 weeks from date e-mail is sent to be inserted here***].**

If you have any questions about this survey, please contact me at Laurence.roope@dph.ox.ac.uk, or on 01865 617913.

Kind regards,

Dr Laurence Roope

Senior Researcher

Health Economics Research Centre

Nuffield Department of Population Health

University of Oxford

Old Road Campus

Headington

Oxford

OX3 7LF

On behalf of the ARK-Hospital Team


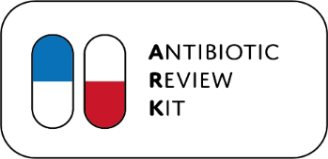

Supplement: Supplementary file 4 — Additional file 4: Recruitment email text. [file 12916_2020_1660_MOESM4_ESM.docx]
