# Supplementary material for: Why do hospital prescribers continue antibiotics when it is safe to stop? Results of a choice experiment survey
Source: BMC Med. 2020 Jul 30;18:196. doi: 10.1186/s12916-020-01660-4 (PMC7391515; doi:10.1186/s12916-020-01660-4)
Supplement: Supplementary file 5 — Additional file 5: Personality questions. Table S1. Mean scores for the five key personality traits. [file 12916_2020_1660_MOESM5_ESM.docx]

**Additional file 5: Personality questions**

**Personality questions**

Mean scores for the so-called ‘big five’ personality traits (Extraversion, Agreeableness, Conscientiousness, Agreeableness and Openness) are reported in Table S1 (n=100). These are based on the responses to ten personality questions (listed below) taken from a short-form questionnaire developed by Rammstedt and John (2007) [23], with the scores constructed using their method.

(1) “I see myself as someone who is reserved.”

(2) “I see myself as someone who is generally trusting.”

(3) “I see myself as someone who tends to be lazy.”

(4) “I see myself as someone who is relaxed, handles stress well.”

(5) “I see myself as someone who has few artistic interests.”

(6) “I see myself as someone who is outgoing, sociable.”

(7) “I see myself as someone who tends to find fault with others.”

(8) “I see myself as someone who does a thorough job.”

(9) “I see myself as someone who gets nervous easily.”

(10) “I see myself as someone who has an active imagination.”

**Table S1: Mean scores for the five key personality traits**

| **Personality trait** | **Combination of personality statements ^a^** | **Mean score ^a^** | **Standard deviation** | **N** |
| --- | --- | --- | --- | --- |
| Extraversion | (1) + (6) | 6.3 | 2.2 | 95 |
| Agreeableness | (2) + (7) | 7.2 | 1.6 | 95 |
| Conscientiousness | (3) + (8) | 8.6 | 1.4 | 94 |
| Neuroticism | (4) + (9) | 4.8 | 1.9 | 94 |
| Openness | (5) + (10) | 6.5 | 1.9 | 95 |

^a^ A higher mean score indicates that on average respondents were more agreeable, open etc.
