# Supplementary material for: Why do hospital prescribers continue antibiotics when it is safe to stop? Results of a choice experiment survey
Source: BMC Med. 2020 Jul 30;18:196. doi: 10.1186/s12916-020-01660-4 (PMC7391515; doi:10.1186/s12916-020-01660-4)
Supplement: Supplementary file 6 — Additional file 6: Attribute rankings and responses to the practice question. Table S2. Attribute rankings. [file 12916_2020_1660_MOESM6_ESM.docx]

**Additional file 6: Attribute rankings and responses to the practice question**

**Table S2** describes the average ranking (from 1-6) given to each of the attributes before and after completing the choice questions.

**Table S2: Attribute rankings**

| **Attribute** | **Before completing choice questions** | | **After completing choice questions** | |
| --- | --- | --- | --- | --- |
|  | **Mean score (SD)** | **Ranking** | **Mean score (SD)** | **Ranking** |
| Patient’s presenting symptoms | 2.2 (1.5) | 1 | 2.6 (1.6) | 2 |
| Risk of significant harm arising from discontinuing antibiotic treatment | 2.4 (1.1) | 2 | 2.5 (1.3) | 1 |
| Risk of significant harm arising from continued antibiotic treatment | 3.1 (1.3) | 3 | 3.1 (1.3) | 3 |
| Premorbid condition of patient | 3.4 (1.3) | 4 | 3.7 (1.5) | 4 |
| Whether early discontinuation of antibiotic treatment within 72 hours of treatment initiation would be in conflict with local antibiotic guidelines | 4.4 (1.4) | 5 | 3.8 (1.6) | 5 |
| Level of external pressure to continue antibiotic treatment | 5.5 (1.0) | 6 | 5.4 (1.2) | 6 |

SD = standard deviation

Before completing the survey, the most important attribute for respondents was ‘Patient’s presenting symptoms’, followed by ‘Risk of significant harm arising from discontinuing antibiotic treatment’. The least important attribute was ‘Level of external pressure to continue antibiotic treatment’. The attribute rankings remained the same after completing the survey, except that the order of the first and second most important reversed. The mean ranking score of ‘Whether early discontinuation of antibiotic treatment within 72 hours of treatment initiation would be in conflict with local antibiotic guidelines’ decreased but not enough to change its ranking from 5^th^ to 4^th^.

**Practice question**

95/100 respondents (95%) answered the practice question ‘correctly’ by choosing to discontinue antibiotics. This question was designed so that a decision to discontinue antibiotics was as likely as possible to be regarded as the best choice that respondents could make in terms of the levels selected for each attribute. However, the 5 respondents who answered this question ‘incorrectly’ were not excluded from this analysis. This is because, for example, it is possible that for these respondents even an extremely low risk from discontinuing antibiotics could be enough to persuade them to continue.
