# Supplementary material for: Why do hospital prescribers continue antibiotics when it is safe to stop? Results of a choice experiment survey
Source: BMC Med. 2020 Jul 30;18:196. doi: 10.1186/s12916-020-01660-4 (PMC7391515; doi:10.1186/s12916-020-01660-4)
Supplement: Supplementary file 7 — Additional file 7: Continue/Discontinue split across the 15 choice questions. Table S3. Continue/Discontinue split. [file 12916_2020_1660_MOESM7_ESM.docx]

**Additional file 7: Continue/Discontinue split across the 15 choice questions**

**Table S3: Continue/Discontinue split**

|  | **Continue** | **Discontinue** |
| --- | --- | --- |
| Q1 | 93 | 7 |
| Q2 | 95 | 5 |
| Q3 | 69 | 31 |
| Q4 | 79 | 21 |
| Q5 | 29 | 71 |
| Q6 | 31 | 69 |
| Q7 | 49 | 51 |
| Q8 | 45 | 55 |
| Q9 | 56 | 44 |
| Q10 | 92 | 8 |
| Q11 | 99 | 1 |
| Q12 | 78 | 22 |
| Q13 | 79 | 21 |
| Q14 | 94 | 6 |
| Q15 | 44 | 56 |
