# Supplementary material for: Why do hospital prescribers continue antibiotics when it is safe to stop? Results of a choice experiment survey
Source: BMC Med. 2020 Jul 30;18:196. doi: 10.1186/s12916-020-01660-4 (PMC7391515; doi:10.1186/s12916-020-01660-4)
Supplement: Supplementary file 8 — Additional file 8: Robustness Analysis: Mixed effects logistic regression analysis. Table S4. Mixed effects logistic regression analysis. [file 12916_2020_1660_MOESM8_ESM.docx]

**Additional file 8: Robustness Analysis: Mixed effects logistic regression analysis**

Conditional logistic models allowed for unobserved heterogeneity between respondents by including a fixed effect. A limitation of these models is that they assume that coefficients of the attributes are the same across individuals. As a robustness check, we also therefore estimated a mixed effects logistic model, which allows the coefficients of the attributes to vary across individuals – i.e. to have ‘random effects’. There were convergence problems when all attributes were allowed to have random effects. To decide which attributes to allow random effects for, the models were then re-estimated, allowing just one attribute at a time to have a random effect. The estimated variance of the random slopes was found to be very small for ‘CONTINUE RISK’, ‘STOP RISK’, ‘No pressure’ and ‘Some pressure’. A mixed logistic model was then estimated with all attribute-levels except these four assumed to have random effects.

This model successfully converged. The results (Table S4) were found to be broadly similar to those in the conditional logistic model (Table 3 in main text), with similar marginal effects.

**Table S4: Mixed effects logistic regression analysis**

| **Attribute ^a^** | **Coefficient** | **SE** | **Lower CI** | **Upper CI** | **Marginal effect** | ***P*** |
| --- | --- | --- | --- | --- | --- | --- |
| SYMPTOMS  UTI symptoms with kidney pain | 1.342 | 0.213 | 0.924 | 1.759 | 0.149 | <0.001 |
| Fever, cough and possible pulmonary infiltrates on chest X-Ray | 0.303 | 0.145 | 0.020 | 0.586 | 0.038 | 0.015 |
| Unclear symptoms **^b^** | -1.644 | - | - | - | - | - |
| CONFLICT WITH GUIDELINES  Strongly conflict | 1.568 | 0.190 | 1.195 | 1.941 | 0.170 | <0.001 |
| Somewhat conflict | 0.103 | 0.120 | -0.132 | 0.338 | 0.010 | 0.408 |
| No conflictb | -1.671 | - | - | - | - | - |
| CONTINUE RISK | -0.109 | 0.017 | -0.142 | -0.075 | -0.012 | <0.001 |
| STOP RISK | 0.217 | 0.025 | 0.169 | 0.265 | 0.024 | <0.001 |
| PREMORBID CONDITION  Severe frailty & comorbidities | 0.741 | 0.177 | 0.394 | 1.089 | 0.082 | <0.001 |
| Moderate frailty & comorbidities | 0.410 | 0.123 | 0.168 | 0.652 | 0.044 | 0.001 |
| Fit and well **^b^** | -1.152 | - | - | - | - | - |
| EXTERNAL PRESSURE  No pressure | -0.829 | 0.149 | -1.122 | -0.536 | -0.091 | <0.001 |
| Some pressure | -0.140 | 0.128 | -0.390 | 0.110 | -0.015 | 0.28 |
| Heavy pressure **^b^** | 0.969 |  |  |  |  |  |
| AIC / BIC | 1384.704/1480.342 |  |  |  |  |  |
| Log pseudo likelihood | -674.35196 |  |  |  |  |  |
| N | 1,500 |  |  |  |  |  |

NOTES: AIC = Akaike Information Criterion. BIC = Bayesian Information Criterion. CI = 95% confidence interval. *P* = p value of coefficients. SE = standard error clustered at respondent level. ^a^Attribute descriptions: SYMPTOMS = Patient’s presenting symptoms (1 = UTI & Kidney, 2 = Fever cough & funny X-Ray, 3 = Unclear [base level]); CONFLICT = Whether early discontinuation of antibiotic treatment within 72 hours of treatment initiation would be in conflict with local antibiotic guidelines (1 = Strongly conflict, 2 = Somewhat conflict, 3 = Not conflict [base level]); CONTINUE RISK = Risk of significant harm arising from continued antibiotic treatment, expressed as a percentage; STOP RISK = Risk of significant harm arising from discontinuing antibiotic treatment, expressed as a percentage; PREMORBID = Premorbid condition of patient (1 = Severe frailty & comorbidities, 2 = Moderate frailty & comorbidities, 3 = Fit and well [base level]); EXTERNAL PRESSURE = Level of external pressure to continue antibiotic treatment (1 = No pressure, 2 = Some pressure, 3 = Heavy pressure [base level]). Sample size arises from 100 respondents times 15 choice questions. ^b^These attributes were effects-coded. The coefficients of the levels that were dropped (Unclear symptoms, No conflict, Fit and well, and Heavy pressure) were calculated as the negative sum of the coefficients of the other levels.

The model in Table S4 contained all 100 respondents (100x15 responses=1500 observations), including the 2 respondents who chose ‘continue’ in all 15 choice questions and were therefore omitted in the conditional logistic regression because of this lack of variation in their responses. To facilitate comparability between the two models in terms of AIC and BIC, the model in Table S4 was also estimated on just the 98 respondents. The results were extremely similar as those in Table S4. As in the model in Table S4, the AIC (1367.347) and BIC (1462.621) in this model were much higher than those in the conditional logistic regression (AIC=976.767; BIC=1029.697), indicating that the conditional logistic model is better quality than the mixed effects logistic models in terms of the trade-off between model simplicity and goodness of fit.
