# Supplementary material for: Why do hospital prescribers continue antibiotics when it is safe to stop? Results of a choice experiment survey
Source: BMC Med. 2020 Jul 30;18:196. doi: 10.1186/s12916-020-01660-4 (PMC7391515; doi:10.1186/s12916-020-01660-4)
Supplement: Supplementary file 9 — Additional file 9: Association of choices with respondent characteristics. Table S5. Ordered Probit Models of Continuing Antibiotics. [file 12916_2020_1660_MOESM9_ESM.docx]

**Additional file 9: Association of choices with respondent characteristics**

Being a consultant was associated with being less likely to continue prescribing antibiotics, i.e. in terms of the total number of times respondents chose to continue antibiotics across the 15 choice questions (Table S5). This association remained after adjusting for personality traits and attitudes to risk. Extraversion was associated with being more likely to choose to continue antibiotics; agreeableness was associated with being less likely to continue antibiotics.

**Table S5. Ordered Probit Models of Continuing Antibiotics**

| Dependent variable: Number of times respondent chose to “continue” | | |
| --- | --- | --- |
|  | Model 1 | Model 2 |
| Male | -0.012  (0.212) | -0.172  (0.237) |
| 500-1000 Beds | -0.412  (0.274) | -0.450  (0.289) |
| >1000 Beds | -0.381  (0.298) | -0.407  (0.351) |
| Consultant | -0.974**  (0.289) | -1.330***  (0.346) |
| Core Medical Trainee | -0.206  (0.317) | -0.455  (0.355) |
| Gen Practice Specialty Registrar or Specialty Trainee/Registrar | -0.196  (0.290) | -0.617  (0.328) |
|  |  |  |
| Risk |  | -0.010  (0.082) |
| Extraversion |  | 0.162*  (0.065) |
| Agreeableness |  | -0.156*  (0.070) |
| Conscientiousness |  | 0.040 |
|  |  | (0.066) |
| Neuroticism |  | -0.029  (0.084) |
| Openness |  | -0.066  (0.059) |
|  |  |  |
| N | 100 | 93 |
| AIC | 449.495 | 420.602 |
| BIC | 493.783 | 478.852 |
| Pseudo R-Sq | 0.035 | 0.067 |

Note: 1. *=p<0.05; **=p<0.01; ***=p<0.001. 2. Personality variables measured on scale of 2-10; Risk score measured on scale of 0-10.
