# Supplementary material for: Why do hospital prescribers continue antibiotics when it is safe to stop? Results of a choice experiment survey
Source: BMC Med. 2020 Jul 30;18:196. doi: 10.1186/s12916-020-01660-4 (PMC7391515; doi:10.1186/s12916-020-01660-4)
Supplement: Supplementary file 10 — Additional file 10: Respondent comments on survey. [file 12916_2020_1660_MOESM10_ESM.docx]

**Additional file 10: Respondent comments on survey**

***Respondent 1. General Practice Specialty Registrar or Specialty Trainee/Registrar, acute/general medicine***

*Possibile infiltrates on Xray is a bit daft. It is a fairly clear cut test in most patients. Appreciate you are trying to achieve some level of ambiguity but to me at least there is a difference between 'possible UTI' which usually as little or no objective verification and someone who has more organ specific symptoms and x-ray changes. As I understand the chance of primary or secondary bacterial infection if the patient has COPD is >80% so knowledge of this would shift the decision threshold to me.*

*Finally why 72 hours, is this not being deliberately contrary? I dont really see what information my answers at least afford. I am very pro antibotic stewardship both not starting and rationalising/stopping which I think largely comes down to a combination of a willingness to make a positive decision rather than another days deference of the same decision and some common sense. Without any supportive information I'm not sure that I am able to articulate that.*

*In short while I assume there is lots of clever psychology and statistics going into this I thought it was largely complete nonsense. designed to give a publishable headline.*

***Respondent 2. Core medical trainee***

*Regarding antibiotic usage, I think secondary care in the UK contributes relatively to little to issues with antibiotics. I think main focus should be put towards excessive antibiotic usage in farm animals. And then ease of access to antibiotics in many countries outside the UK. Prescribing in primary care already seems quite monitored and guidelines in place.*

***Respondent 3. General Practice Specialty Registrar or Specialty Trainee/Registrar, acute/general medicine***

*Strange survey. There's no mention of patient response to antibiotics, or cultures. I care most about whats going on with the patient, obs, cultures, Temps, bloods, the severity of presentation and risk of deterioration. I start or stop antibiotics based on the information to hand and support my decision in the notes. No accounting for asking microbiology advice or switching to oral/ alternative when cultures/bloods come back which is a big part of the decision process?*

***Respondent 4. Consultant, acute/general medicine***

*I think that there should have been information about the clinical status of the patient at the time of the antibiotic review as well as at presentation. If the patient is still unwell or deteriorating, I would be more likely to continue with antibiotics and stop if the patient is clinically Improving. Without this information, decisions about stopping or continuing antibiotics are difficult to make.*

***Respondent 5. Consultant, acute/general medicine***

*Not great. Not clinically detailed enough. Eg if sepsis have they had temperatures, how high the inflammatory markers etc*

***Respondent 6. Consultant, acute/general medicine***

*The level of harm that is portrayed in the survey is easy to digest however the level of harm from either continuing or discontinuing antibiotics in real world clinical practice is usually extremely difficult to quantify and weigh up in prescribing decisions.*

***Respondent 7. Core medical trainee, acute/general medicine***

*Thank you for taking the time to research this important topic.*

*In short, I am happy to make bold decisions regarding anti-microbial. However, when the stakes are high (high risk of a poor outcome with either course of action) I tend to follow the guideline, or, seek a more senior opinion if I believe the guideline recommendation to be not in the patients best interest.*

***Respondent 8. Consultant, acute/general medicine***

*would have been good to have an idea of how the patient is responding - often decision to stop treatment in a frail patient is based on a failure to respond to initial 48 hours of treatment, implying that their underlying frailty / comorbidities are playing a greater part in their presentation than the infection / antibiotic therapy*

***Respondent 9. Consultant, acute/general medicine***

*Clinical scenarios very hard to make risk assessment on based on information given. Inflammatory markers, culture results, NEWS scores, clinical assessment of the patient all would influence my decision hugely.*

***Respondent 10. Consultant, acute/general medicine***

*Yes. When ranking information before the scenarios, by picking patient's symptoms and premorbid condition as top priorities, what I was trying to convey was that degree of confidence in the diagnosis and knowledge about the patient's frailty (or otherwise) are probably the most important factors when making antibiotic decisions about real patients, because it is from those pieces of information that the estimates about likely harm and benefit may be derived. What you did in your questionnaire was make these factors irrelevant because of your provision of estimates of benefit or harm that were separate from these factors. I am concerned therefore that by doing this, the situational questionnaire you have used may give you false information about how real clinicians make these decisions or rank these factors.*

***Respondent 11. Consultant, acute/general medicine***

*It's all in the history and examination for me I am afraid. I would not make decisions to stop/continue antibiotics on these cases in real life without being able to look at observations, bloods, chat with the patient, clinical signs...I know it is difficult, but this is over simplifying!*

***Respondent 12, Consultant, Microbiology/Infectious Diseases***

*I would not usually use the criteria you have offered to make these decisions about stopping antibiotics. I would want to know if the patient was clinically improving, if their parameters were improving, if there were confirmed microbiological results. It is salutary to realised how much attention I would pay to resistance about stopping antibiotics with regards to continuation. In real life I rarely come across this scenario because of the above.*

***Respondent 13, Consultant, acute/general medicine***

*Some questions, like the one on % were not that clear and relevant*

***Respondent 14, Staff Grade/Associate specialist, acute/general medicine***

*Interesting survey! I changed my ranking quite a lot, which I was surprised by, though it was apparent as I was actually doing the questions where my priorities for assessment actually lie, as opposed to where I thought they lay.*

***Respondent 15, General Practice Specialty Registrar or Specialty Trainee/Registrar, Rheumatology***

*Its hard to make a realistic answer - the risks of continuing/discontinuing are never that definite (or even known)*

***Respondent 16, Consultant, acute/general medicine***

*Difficult to make decisions re stopping/continuing when you don't have the most recent clinical picture (ie whether the symptoms/signs have changed since initial presentation, what the current obs are etc). I didn't find the info that one had to make a decision based on very helpful at all, it wouldn't be what I would base my decision on in real life.*

***Respondent 17, Foundation Year Two doctor, Respiratory***

*Option to know how patient responded to the 72hours of antibiotics. For example vital signs, bloods markers.*

***Respondent 18, Core Medical Trainee, acute/general medicine***

*If unsure, the answer on the wards is usually to continue a 5 day course of antibiotics.*

***Respondent 19, Core Medical Trainee, geriatric medicine***

*An important part of my decision about whether to continue antibiotics or not is whether the patient is improving symptomatically at the 72 hour point and whether they've been apyrexial for >24 hours or not. This information was not provided in the question.*

*Patients with symptoms clearly suggestive of pyelonephritis or a pneumonia I would want them to continue the full course of antibiotics, which would be longer than 72 hours, so I wouldn't generally stop their antibiotics at 72 hours, just convert to oral alternatives.*

*Patients who are frail and elderly who probably never had an infection in the first place would be the people I would be most likely to stop their antibiotics, if I felt there were other causes of their symptoms. Or if a source of infection had become more clear I may change their antibiotic type, rather than stop antibiotics overall.*

***Respondent 20, General Practice Specialty Registrar or Specialty Trainee/Registrar, respiratory***

*I had an issue about stopping abx after 72 hours which I would rarely do unless an alternative cause has been found hence many of my responses to continue abx.*

***Respondent 21, General Practice Specialty Registrar or Specialty Trainee/Registrar,***

***Microbiology/Infectious Diseases***

*The percentage decisions question in this survey was quite difficult to answer and may not have reliable accuracy*

***Respondent 22, General Practice Specialty Registrar or Specialty Trainee/Registrar, locum surgical/orthopaedics***

*The percentage question wasn’t clear, is that assessing antibiotics at 72 hours or all decisions over a year?*

***Respondent 23, General Practice Specialty Registrar or Specialty Trainee/Registrar, acute/general medicine***

*Future iterations may want to make sure that this survey is more mobile friendly. I was also unsure if my 'personality questions' related to my work or my life. In life I take risks, at work, a lot less for example.*

***Respondent 24, General Practice Specialty Registrar or Specialty Trainee/Registrar, non-infection related medical specialty***

*Ranking system didn’t work that well on an iPhone*

***Respondent 25, Core Medical Trainee, acute/general medicine***

*Well put together, excellent presentation of attributes - would be easier if they could all be viewed simultaneously*

*Difficult to weigh up harms and benefits as they were so clear cut. If could be presented like this in clinical practice this would be much easier to make decisions.*

***Respondent 26, General Practice Specialty Registrar or Specialty Trainee/Registrar, acute/general medicine***

*I would consider external pressure from family and team differently esp. consultant*

*If there's heavy pressure from consultant, their word is what goes, ultimately- they're responsible for the patient.*

*If heavy pressure from family, I'd explore their reasons. Where the decision about antibiotics is very obvious, influence will be minimal and is a matter for good communication. Family pressure would influence my decision more in genuine grey cases.*

***Respondent 27, Consultant, Microbiology/Infectious Diseases***

*I found the methodology very challenging to comprehend, it requires a high level of abstraction and concentration. I also felt that the scenarios were very generic and far off the clinical reality. There are so many more factors that could play a role in decision-making. The most important one: the current clinical status of the patient and how he/she responded to the initial treatment. That is a major determinant in stop/continue decisions, and is not included here (maybe purposely). I am not convinced whether the data you will get from this survey will actually give you reliable answers. But I would be very much interested to see the results nonetheless! Good luck with the work.*

***Respondent 28, Core Medical Trainee, acute/general medicine***

*The other factor I would consider is the change in patient clinical status from admission / commencement of anitbiotic therapy to the point of review/*

***Respondent 29, General Practice Specialty Registrar or Specialty Trainee/Registrar,***

***general surgery***

*Your survey is daft.*

*Pneumonia - 5 days abx*

*Pyelonephritis - 7 days at least of abx*

*Vague presentation without obvious cause - stop abx*

***Respondent 30, Consultant, Microbiology/Infectious Diseases***

*In retrospect I think the clinical vignettes may have influenced my own assessment of risks of continuing/discontinuing independent of the risk percentages presented for some of the questions. Sorry!*

***Respondent 31, Consultant, acute/general medicine***

*The current condition of the patient /response to treatment so far is often a critical factor but we didn't have this information available. By 72 hours We would normally have got more clarity and cultures back etc*

***Respondent 32, Consultant, acute/general medicine***

*Guidelines are too risk averse. Risk of recurrence of infection in many cases is if no great consequence. Eg recurrence of uti in elderly. We believe antibiotics do much more than they actually do in frailty patients.*

*Many old people are being kept alive in a miserable condition by inappropriate use of antibiotics in what are really end of life situations. We eke out these lives for a few months more with antibiotics - I certainly don’t want this for myself or my elderly relatives, but society has not prepared patients or their families for the potential misery of the last 6-12 months of their life. Wise and articulate people avoid this.*

***Respondent 33, Consultant, geriatric medicine***

*The answers were difficult because the decisions usually involves assessing how unwell people are, how well they are responding to treatment, ongoing evidence of infection, evidence of accuracy of diagnosis and evidence of collections, history of bronchiectasis etc. These factors matter more.*

***Respondent 34, Foundation Year One doctor, acute/general medicine***

*Very difficult to relate to 'moderate comorbidities' etc. was difficult to understand the context and make a decision that reflects my practice in reality.*
